# Effect of Indole-Containing Pyrazino[2,1-*b*]quinazoline-3,6-diones in the Virulence of Resistant Bacteria

**DOI:** 10.3390/antibiotics12050922

**Published:** 2023-05-17

**Authors:** Mariana C. Almeida, Nikoletta Szemerédi, Fernando Durães, Solida Long, Diana I. S. P. Resende, Paulo Martins da Costa, Madalena Pinto, Gabriella Spengler, Emília Sousa

**Affiliations:** 1 Laboratório de Química Orgânica e Farmacêutica, Faculdade de Farmácia, Universidade do Porto, Rua de Jorge Viterbo Ferreira 228, 4050-313 Porto, Portugal; mariana_c_a@hotmail.com (M.C.A.); fduraes5@gmail.com (F.D.); solidachhann@gmail.com (S.L.); madalena@ff.up.pt (M.P.); 2 CIIMAR––Centro Interdisciplinar de Investigação Marinha e Ambiental, Terminal de Cruzeiros do Porto de Leixões, 4450-208 Matosinhos, Portugal; pmcosta@icbas.up.pt; 3 Department of Medical Microbiology, Albert Szent-Gyorgyi Health Center and Albert Szent-Gyorgyi Medical School, University of Szeged, Semmelweis utca 6, 6725 Szeged, Hungary; szemeredi.nikoletta@med.u-szeged.hu (N.S.); spengler.gabriella@med.u-szeged.hu (G.S.); 4 Department of Bioengineering, Royal University of Phnom Penh, Russian Confederation Blvd, Phnom Penh 12156, Cambodia; 5 ICBAS—Instituto de Ciências Biomédicas Abel Salazar, Universidade do Porto, Rua de Jorge Viterbo Ferreira 228, 4050-313 Porto, Portugal

**Keywords:** antibacterial, efflux pump inhibition, antibiofilm, pyrazino[2,1-*b*]quinazoline-3,6-diones

## Abstract

Drug resistance is rising to alarming levels, constituting one of the major threats to global health. The overexpression of efflux pumps and the formation of biofilms constitute two of the most common resistance mechanisms, favoring the virulence of bacteria. Therefore, the research and development of effective antimicrobial agents that can also counteract resistance mechanisms are extremely important. Pyrazino[2,1-*b*]quinazoline-3,6-diones, from marine and terrestrial organisms and simpler synthetic analogues, were recently disclosed by us as having relevant antimicrobial properties. In this study, using a multi-step approach, it was possible to synthesize new pyrazino[2,1-*b*]quinazoline-3,6-diones focusing on compounds with fluorine substituents since, to the best of our knowledge, the synthesis of fluorinated fumiquinazoline derivatives had not been attempted before. The new synthesized derivatives were screened for antibacterial activity and, along with previously synthetized pyrazino[2,1-*b*]quinazoline-3,6-diones, were characterized for their antibiofilm and efflux-pump-inhibiting effects against representative bacterial species and relevant resistant clinical strains. Several compounds showed relevant antibacterial activity against the tested Gram-positive bacterial species with MIC values in the range of 12.5–77 μM. Furthermore, some derivatives showed promising results as antibiofilm agents in a crystal violet assay. The results of the ethidium bromide accumulation assay suggested that some compounds could potentially inhibit bacterial efflux pumps.

## 1. Introduction

Antimicrobial resistance has been rising and it is already considered one of the major threats to global health [1,2], with previsions reckoning a serious aggravation of this problem in the near future [3,4,5]. Without effective antimicrobials, the mortality rate caused by infectious diseases will continue to rise to very concerning levels and, considering the current connections and traveling rates of the human population, the emergence of new global pandemics with devastating effects becomes increasingly more likely [6,7]. Therefore, it is clear that the research and development of new compounds with antimicrobial properties and compounds that can decrease resistance to known antimicrobials is now more important than ever.

Microorganisms can become resistant to antibiotics through various mechanisms, such as target alterations, destruction or alteration of the antimicrobial molecule, and decreased permeability or augmented efflux to reduce the quantity of antibiotic accumulated inside the microbial cells [8,9]. Efflux systems are present in bacteria to pump toxic molecules out of the bacterial cells. Mutational events can lead to the overexpression or to an increase in the effectiveness of efflux pumps that are capable of transporting multiple different drugs [8,10]. For this reason, multidrug efflux pumps cause both intrinsic and adaptative resistance in bacteria [8]. There is also evidence that efflux pumps might be involved in biofilm formation, another major cause of antimicrobial resistance [11,12]. The AcrAB–TolC system in *Escherichia coli* is one of the most studied multidrug resistance efflux pumps [13,14]. It is constituted by the outer membrane channel TolC, the inner membrane secondary transporter AcrB, and the periplasmic AcrA that connects the other two proteins [13]. The AcrAB–TolC efflux pump is capable of transporting many compounds with a high diversity of chemical structures being involved in resistance to a variety of antibiotics [13,15,16]. It has also been suggested that indole derivatives might have potential as AcrAB–TolC inhibitors [17].

**Figure 1:**
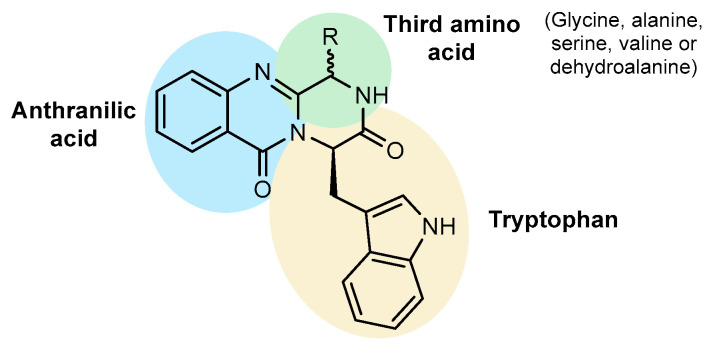
Indolylmethyl pyrazino[2,1-*b*]quinazoline-3,6-dione core structure (adapted from Resende et al. [19]).

Natural products have been used for the treatment of diseases since ancient times and they have given a major contribution to drug discovery. This is especially true when it comes to antimicrobial drugs, with more than three-quarters of the currently used antibiotics corresponding to natural products or their derivates, mostly those produced by fungi and bacteria, both terrestrial and marine [18]. Fumiquinazolines and related alkaloids, comprising a pyrazino[2,1-*b*]quinazoline-3,6-dione core linked to an indole moiety (Figure 1), are an example of natural products that show promising biological activities, including antimicrobial properties. These compounds can be found in terrestrial and marine organisms, mainly in fungi [19], and show promising antimicrobial activities, including antiviral, antifungal, and antibacterial properties [19]. Additionally, synthetic analogues reported by our group have been shown to have a neuroprotective capacity and potential as antimalarial agents [20,21,22].

**Figure 2:**
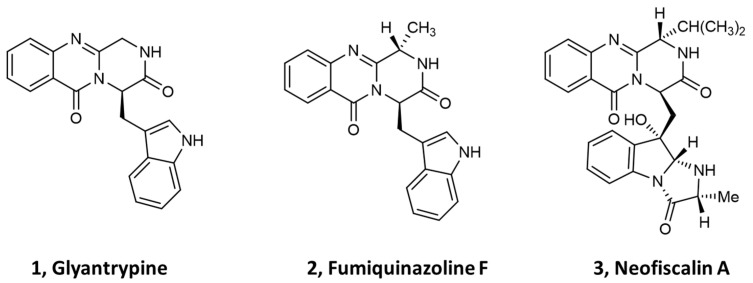
Naturally occurring fumiquinazoline-related alkaloids **1**–**3** with promising antimicrobial activities.

Regarding glycine-derived alkaloids, glyantrypine (**1**, Figure 2) is one of the simplest molecules in the fumiquinazoline family, being derived from anthranilic acid, tryptophan, and glycine. This compound has exhibited anti-viral properties against H1N1 (IC_50_ = 100–150 µM) [23] as well as antibacterial activity against aqua-pathogenic *Vibrio harveyi* [minimum inhibitory concentration (MIC) = 32 µg/mL]. Among the alanine-derived alkaloids, fumiquinazoline F (**2**, Figure 2) as well as fumiquinazolines A and G (structures not shown) have shown potential as antifungal agents, being active against several phytopathogenic fungi (MIC = 12.5–25 μg/mL) [24]. Neofiscalin A (**3**, Figure 2) has exhibited activity against Gram-positive bacteria, namely against sensitive and methicillin-resistant strains of *Staphylococcus aureus* (MIC = 8 µg/mL), as well as against sensitive and vancomycin-resistant strains of *Enterococcus faecalis* (MIC = 8 µg/mL) [25]. In addition, neofiscalin A (**3**) has also displayed an antibiofilm capacity and showed no cytotoxicity against a human brain endothelial cell line (hCMEC/D3), making this compound a promising lead for the development of new antimicrobial drugs [25].

**Figure 3:**
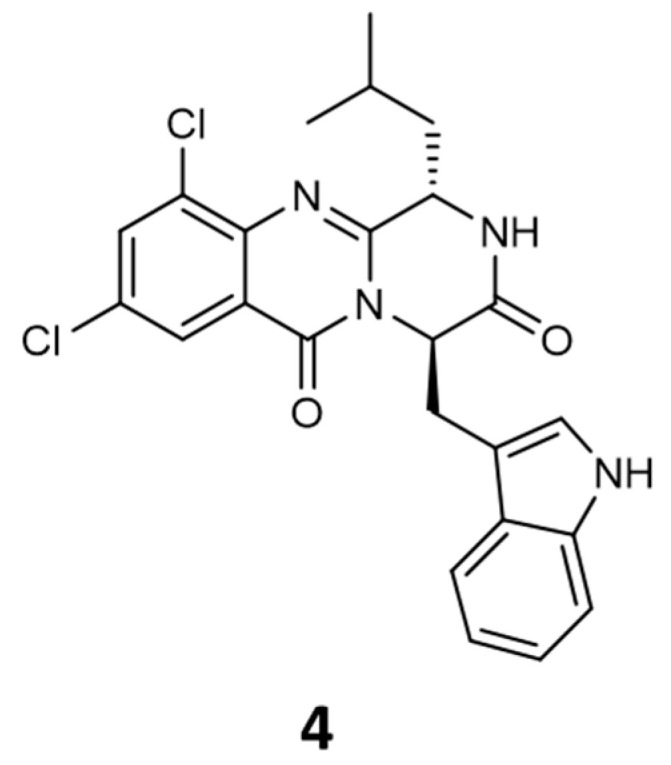
Synthetic fumiquinazoline derivative **4**.

Regarding synthetic derivatives related to fumiquinazolines, Long et al. [26] have recently reported a series of halogenated derivatives with antibacterial profiles. Dichlorinated alkaloid **4** (Figure 3) emerged as a hit compound showing promising activity against one sensitive and one resistant strain of *S. aureus* with MIC values of 4 and 8 µg/mL, respectively [26].

The main goal of this work was to expand the previously reported library of halogenated derivatives related to the fumiquinazolines [26] particularly focusing on compounds with fluorine substituents since, to the best of our knowledge, the synthesis of fluorinated fumiquinazoline derivatives has never been attempted before. We also aimed to evaluate the antibacterial, antibiofilm, and efflux pump inhibition activities of the synthesized compounds and some previously reported derivatives.

## 2. Results and Discussion

### 2.1. Synthesis and Structure Elucidation

**Scheme 1:**
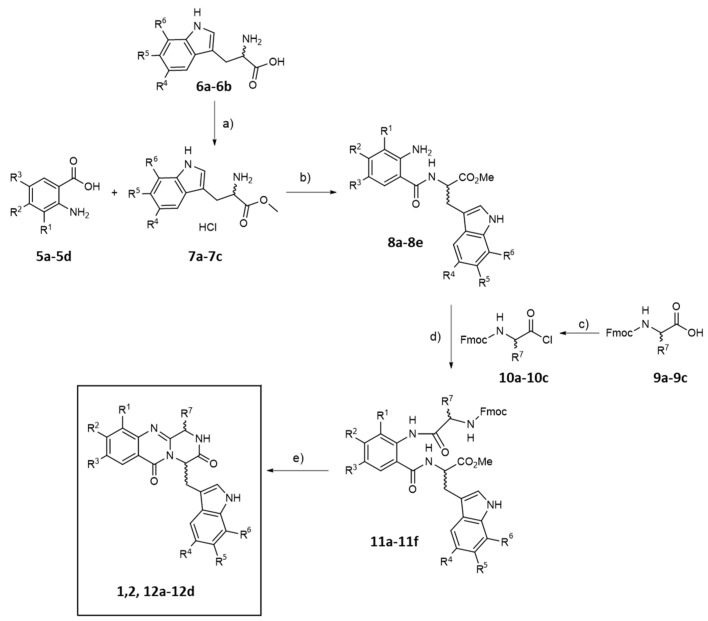
General multi-step synthetic pathway to compounds **1**, **2**, and **12a**–**12d**. (**a**) SOCl_2_, dry MeOH, overnight; (**b**) 2-(1*H*-benzotriazole-1-yl)-1,1,3,3-tetramethylaminium tetrafluoroborate (TBTU), Et_3_N, CH_3_CN, rt, 5 h; (**c**) SOCl_2_, dry CH_2_Cl_2,_ 30 min; (**d**) 1. Dry CH_2_Cl_2,_ 2. Aq Na_2_CO_3_ (1M); (**e**) 1. Ph_3_P, I_2_, *N*,*N*-diisopropylethylamine (DIPEA), CH_2_Cl_2,_ rt, for glyantrypine (**1**): 2. CH_2_Cl_2_, 20% piperidine 3. CH_3_CN, reflux, 24 h; for fumiquinazoline F (**2**), **12a**–**12d**: 2. 25% piperidine in EtOAc, 1 h; 3. Silica gel, EtOAc:MeOH (2:1), rt, overnight.

A multi-step approach, based on the Mazurkiewicz–Ganesan method [27], was employed for the synthesis of the fumiquinazoline-related derivatives (Scheme 1). This pathway concerns three main steps: (i) synthesis of a linear dipeptide **8** (ii) synthesis of a linear tripeptide **11** and (iii) cyclization of the linear tripeptide into the desired fumiquinazoline derivatives **1**, **2**, and **12a**–**12d**.

Since the tryptophan derivatives required to synthesize dipeptides **8d** and **8e** were not available in the form of methyl ester hydrochlorides (**7b** and **7c**), an initial esterification reaction catalyzed by SOCl_2_ in anhydrous methanol was performed according to a previously described procedure [28]. They were used in the following reaction without further purification to avoid yield losses.

**Figure 4:**
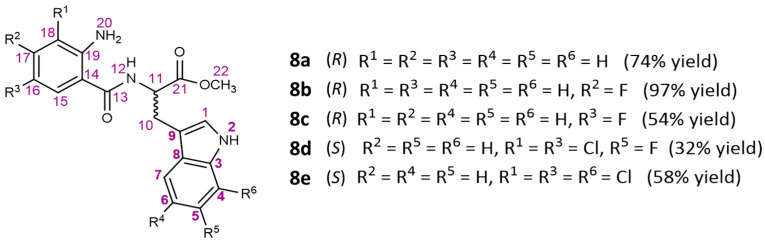
Synthesized linear dipeptides **8a**–**8e**.

The linear dipeptides **8a**–**8e** were synthesized through a coupling reaction between the appropriate anthranilic acid (**5**) and tryptophan methyl ester derivatives (**7**), in the presence of the coupling agent 2-(1*H*-benzotriazole-1-yl)-1,1,3,3-tetramethylaminium tetrafluoroborate (TBTU) and triethylamine (Et_3_N) at room temperature (rt) for 5 h (Scheme 1) [29]. It was possible to effectively obtain and characterize through nuclear magnetic resonance (NMR) methods linear dipeptide **8a**, which is a precursor for the natural products glyantrypine (**1**) and fumiquinazoline F (**2**), as well as the four halogenated dipeptides **8b**–**8e** (Figure 4).

**Figure 5:**
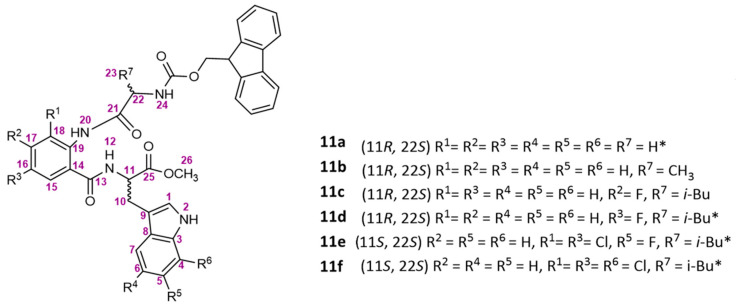
Synthesized linear tripeptides **11a**–**11f**. * Compounds not purified or characterized. i-Bu = iso-Butyl.

Then, the acid chlorides required for the synthesis of the tripeptides **11a**–**11f** were synthesized from their respective 9-fluorenylmethoxycarbonyl (Fmoc)-protected amino acids by treatment with SOCl_2_ in dry CH_2_Cl_2_ (Scheme 1) [30]. It was possible to obtain three acid chlorides: Fmoc-Gly-Cl (**10a**), Fmoc-L-Ala-Cl (**10b**), and Fmoc-L-Leu-Cl (**10c**). Afterwards, the synthesis of the linear tripeptides **11a**–**11f** (Figure 5) was achieved via coupling of the linear dipeptides **8a**–**8e** with the respective Fmoc-protected acid chlorides (**10a**–**10c**) [27]. This reaction was performed under two-phase Schotten–Baumann conditions in which an aqueous base (Na_2_CO_3_) solution was added that would act as a catalyst and shift the equilibrium towards the formation of the tripeptide while also neutralizing the HCl formed in the reaction, preventing the further protonation of the product that remained in the organic phase.

**Scheme 2:**
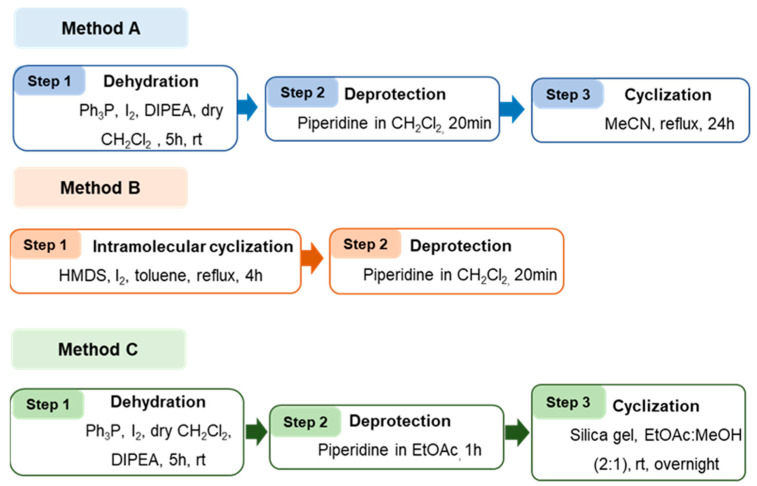
Summary of the methods used for the cyclization of the linear tripeptides.

**Figure 6:**
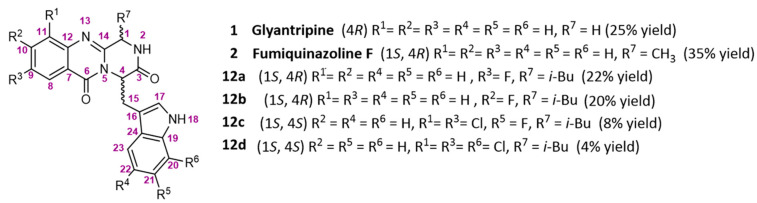
Synthesized derivatives **1**, **2**, and **12a**–**12d** (yields from three or four steps from tripeptides **11** or dipeptides **8**, respectively).

The obtained tripeptides **11b** and **11c** were isolated and it was possible to purify and characterize them. However, in the case of tripeptides **11a**, **11d**, **11e**, and **11f**, they were used without further purification to make the synthetic pathway faster and to avoid yield losses. Three different methods were employed concerning the cyclization of the linear tripeptides, which are summarized in Scheme 2. Method A [27] was employed to synthesize glyantrypine (**1**). It was possible to verify that the intermediate oxazine was not stable during the purification process, and therefore the three reactions were performed sequentially. Purification was carried out by preparative thin layer chromatography (TLC) followed by crystallization in MeOH that allowed the obtainment of glyantrypine (**1**, Figure 6) as white crystals in a 25% yield (four steps from dipeptide **8a**).

Methods B [31] and C [32] (Scheme 2) were then employed for the synthesis of fumiquinazoline F (**2**). With Method B, the ^1^H-NMR spectrum of the obtained compound suggested that it corresponded to fumiquinazoline F (**2**, Figure 6), but showed additional signals indicating that the compound was not pure. After further purification by crystallization in MeOH, only 3 mg was obtained. This method’s main advantage was that it comprised only two steps, making the synthetic pathway faster. However, the final purification of the compound was still hindered by the presence of several secondary products. Furthermore, the first step was performed in reflux in toluene and the high temperature (110 °C) might have led to the racemization of the compounds. With Method C, it was possible to synthesize fumiquinazoline F (**2**, Figure 6) in a 35% yield (from three steps). This method showed fewer secondary products and could be fully performed at room temperature, therefore it was considered a greener approach than Methods A and B. Taking this into account, the third method was applied for the synthesis of the new derivatives. In this work, four halogenated compounds **12a**–**12d** (Figure 6) were obtained (4–22% yields from three or four steps from tripeptides **11** or dipeptides **8**, respectively), that to the best of our knowledge, had never been synthesized before. The employed multi-step synthetic pathway proved to be effective for the synthesis of fumiquinazoline-related derivatives. Nevertheless, it comprised some difficulties, such as the purification of the final compounds, which was hindered by the presence of a significant amount of secondary products formed in the last step, and the instability of some of the intermediates.

The structural elucidation of the synthesized compounds was performed using NMR spectroscopy methods (Figures S1–S47, Supplementary Materials), namely ^1^H-NMR, ^13^C-NMR, heteronuclear single quantum correlation (HSQC), and heteronuclear multiple bond correlation (HMBC), and these confirmed the predicted structures. For the final derivatives, high-resolution mass spectrometry (HRMS) analysis (Figures S48–S51, Supplementary Materials) was also performed and it was in accordance with the predicted structures.

**Figure 7:**
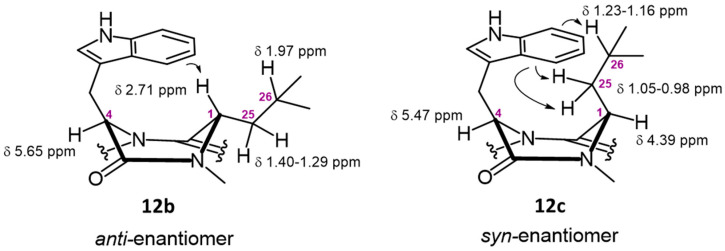
Schematic representation of the conformation of **12b** and **12c** and relevant chemical shifts.

Furthermore, the ^1^H-NMR analysis of the final derivatives also allowed for some interesting observations that are worth highlighting. The characteristic signal associated with H-4 presented a chemical shift of 5.7 ppm, which was in accordance with that described by Long et al. [29] and Hernandez et al. [33] for this proton in other piperazine rings. As explained by those authors, the chemical shift of this signal suggests a boat conformation of the piperazine ring, with H-4 occupying a quasi-equatorial position and therefore being influenced by the anisotropic effect of the coplanar carbonyl group at C-6 (Figure 7).

In addition, the signal associated with H-1 had a chemical shift of 2.71 ppm, which was coherent with the values reported in the literature for similar compounds, namely for the *anti*-enantiomers. For the *anti*-enantiomers, as was the case of **12a** and **12b**, the chemical shifts of the signal associated with H-1 (δ = 2.73 and 2.71 ppm) were lower than those obtained for the *syn*-enantiomers **12c** and **12d** (δ = 4.39 and 4.24 ppm). As is shown in Figure 7, this likely occurred due to the folding of the indolyl substituent over the piperazine ring, in which the aromatic ring of the indolyl substituent would have a shielding effect over H-1 in the *anti*-enantiomers. On the contrary, in the *syn*-enantiomers, this shielding effect was observed in the signals associated with H-25 and H-26 from the isobutyl substituent (δ 2.04–1.29 ppm for the *anti*-enantiomers and δ 1.23–0.66 ppm for the *syn*-enantiomers). This effect had already been reported by Hernandez et al. [33] in similar compounds. Therefore, the analysis of these signals indicates that the compounds did not suffer epimerization during the synthetic process. Melting points and optical rotation of the final derivatives were also measured to further characterize the compounds. The purity of all the compounds tested for biological activity was assessed by high-performance liquid chromatography with photodiode-array detection (HPLC-DAD) (Table 1) (HPLC Data—Tables S1 and S2, Supplementary Materials).

### 2.2. Evaluation of Biological Activities

**Figure 8:**
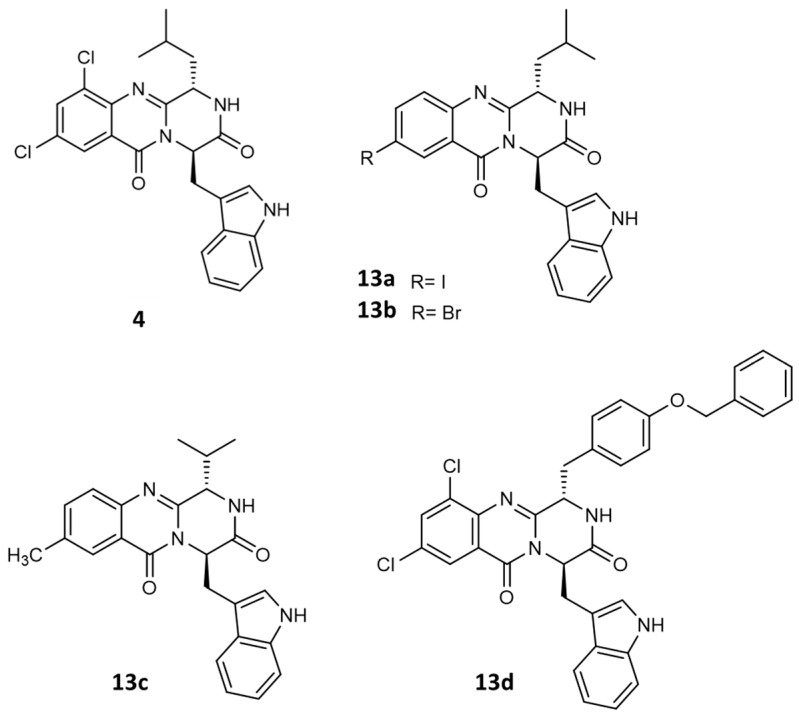
Structures of the fumiquinazoline derivatives **4** and **13a**–**13d** synthesized in previous works.

All synthesized pyrazino[2,1-*b*]quinazoline-3,6-diones (**1**, **2**, **12a**–**12d**) were screened for their antibacterial, antibiofilm, and efflux-pump-inhibiting activities. Since, to the best of our knowledge, there has been no report to date of the screening of these type of fumiquinazoline derivatives for efflux pump inhibition, we decided to extend the screening of the antibiofilm activity and efflux-pump-inhibiting capacity to the related compounds **4** and **13a**–**13d**, synthesized by our group in previous works [20,21,26] (Figure 8).

#### 2.2.1. Antibacterial Activity

The determination of the MIC values of the synthesized compounds **1**–**2** and **12a**–**12d** was performed against four representative bacterial species—*S. aureus* ATCC 29213, *E. faecalis* ATCC 29212, *E. coli* ATCC 25922, and *P. aeruginosa* ATCC 27853—as well as against clinically relevant strains of the same bacterial species, namely methicillin- and ofloxacin-resistant *S. aureus* 272123, methicillin-resistant *S. aureus* (MRSA) 66/1, *E. faecalis* B3/101, *E. coli* SA/2, *E. coli* AG100, and *E. coli* AG100A. In addition, for the compounds that showed activity against *E. faecalis*, two other vancomycin-resistant (VRE) bacterial strains were also tested: PSi/107 and PFi/2. The resistant strain *E. coli* SA/2 and *E. faecalis* B3/101 had reported MIC values of 512 μg/mL against CTX and 1024 μg/mL against vancomycin, respectively [34]. Both reference and resistant strains used in this work were employed by our group for the screening of different families of compounds, such as xanthones and thioxanthones [34,35].

Antibacterial activity results of the previously synthesized derivatives **4**, **13a**, and **13b** have already been reported before [26]. None of the synthesized derivatives **1**, **2**, and **12a**–**12d** showed activity against the Gram-negative bacterial species tested (*P. aeruginosa* and *E. coli*, MIC > 100 µM). In this work, previously synthesized derivatives **4** and **13a**–**13d** were only screened against *E. coli* AG100 and AG100A and showed no activity (MIC > 100 µM). The obtained results of the synthesized compounds **1**, **2**, and **12a**–**12d** and related derivatives **4** and **13a**–**13d** for the Gram-positive bacteria are summarized in Table 2. Compound **12d** showed the most potent activity against *S. aureus*, including the MRSA strains (MIC = 16–32 μM). The value obtained for the MRSA was comparable to that reported for the natural product neofiscalin A (**3**, Figure 2). It is interesting to note that compounds **12a** and **12b** did not show activity against the sensitive strain of *E. faecalis* nor the other two VRE strains tested (PSi/107 and PFi/2), suggesting that these compounds might be selective for this particular strain (*E. faecalis* B3/101).

Additionally, it is important to mention that similar halogenated fumiquinazoline derivatives have been previously screened for antibacterial activity against the same bacterial strains used in this work, with MIC values between 4 and >64 μg/mL for *S. aureus* ATCC 2913 and *S. aureus* 66/1 [26]. None of the previously reported halogenated derivatives showed activity against *E. faecalis* B3/101 (MIC > 64 μg/mL) [26]. Therefore, in this work, it was possible to obtain a synthetic *hit* compound (**12d**) with a broader spectrum of action than the related derivatives previously reported, which was a major advantage and an important step in the optimization of this type of compound towards the development of effective antibacterial agents.

#### 2.2.2. Efflux-Pump-Inhibiting (EPI) Capacity

In this study, the compounds were tested for their ability to inhibit the efflux pumps of Gram-negative and Gram-positive model bacterial strains using a real-time ethidium bromide accumulation assay. The EPI activity was investigated on the *E. coli* AG100 strain, expressing the AcrAB–TolC efflux pump (EP) at its basal level, and its AcrAB–TolC-deleted mutant *E. coli* AG100A, as well as on the *S. aureus* MRSA 272123 strain. The *E. coli* AG100 strain has been reported to have intrinsic efflux activity [36] and is a common strain for efflux-pump-inhibition studies with different compounds [37,38]. Regarding MRSA 272123, previous studies with potential efflux pump inhibitors have also been conducted by our group [34].

**Figure 9:**
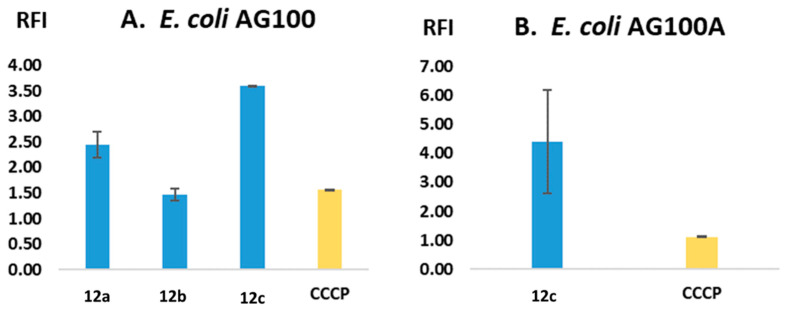

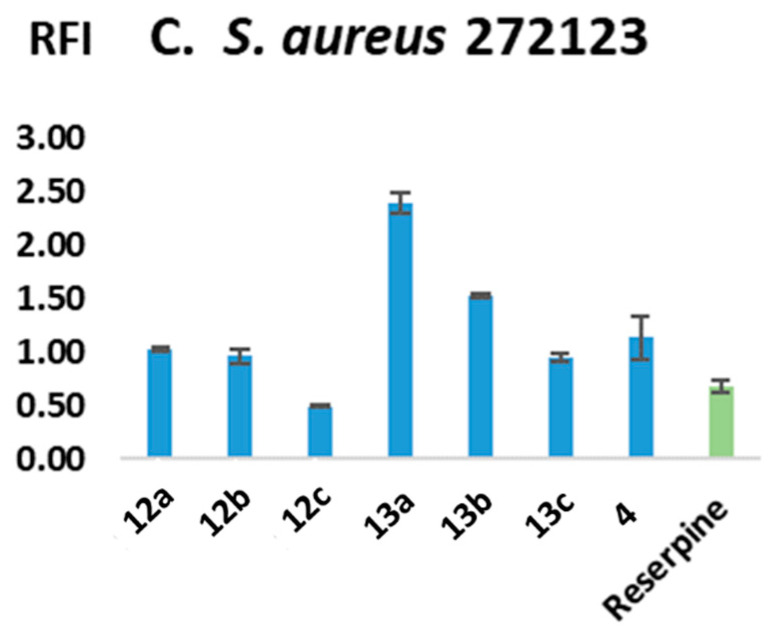
Efflux pump inhibitory activity of the most potent derivatives against *E. coli* and *S. aureus* strains. (**A**) All compounds were added at 100 µM, CCCP was added at 50 µM; (**B**) The compound was added at 100 µM, CCCP was added at 50 µM; (**C**) Compound **12a** was added at 100 μM; **12b**, **13a**–**13c**, and **4** were added at 50 µM; **12c** was added at 6.25 µM; and reserpine was added at 25 µM.

The relative fluorescence index (RFI) was calculated based on the means of relative fluorescence units, as summarized in Table 3 and Figure 9.

In general, derivatives were most effective on the Gram-negative *E. coli* strains. Against *E. coli* AG100, compounds **12a**–**12c** showed promising RFI values when tested at a 100 µM concentration, with compounds **12a** and **12c** showing more potent activities than the positive control carbonyl cyanide m-chlorophenyl hydrazone (CCCP). Regarding the *E. coli* AG100A strain, only compound **12c** showed higher RFI values than CCCP. Interestingly, compound **12c** showed a more potent activity against *E. coli* AG100A than against *E. coli* AG100, suggesting that this compound might inhibit efflux pumps other than AcrAB–TolC. Compounds **12a**–**12c**, **4**, and **13a**–**13c** showed relevant EPI activity against *S. aureus* 272123 (MRSA) when tested at a 50 µM concentration or at half of the MIC concentration. Based on these results, it can be concluded that **12c** proved to be the most potent, as it was able to exert EPI activity in three bacterial strains: on *E. coli* AG100 at 100 and 50 µM, on *E. coli* AG100A at 100 µM, and on the MRSA strain at an MIC/2 concentration (6.25 μM). Furthermore, it had more potent activity against the two *E. coli* strains tested when compared to the positive control CCCP.

#### 2.2.3. Antibiofilm Activity

**Figure 10:**
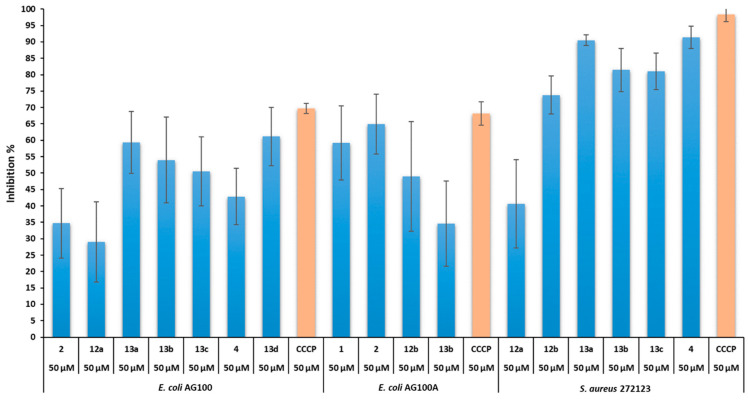
Antibiofilm activity of the derivatives with the most potent activities against *E. coli* AG100, *E. coli* AG100A, and *S. aureus* MRSA 272123 strains. This figure shows the results of three individual experiments for each bacterial strain to determine the compounds’ antibiofilm effect. CCCP was used as a positive control for each strain.

The antibiofilm effect of the tested compounds was evaluated using the crystal violet method on *E. coli* AG100, *E. coli* AG100A, and MRSA 272123. Compounds **2**, **4**, **12a**, and **13a**–**13d** reduced the amount of biofilm formed by *E. coli* AG100 when tested at a 50 μM concentration. Furthermore, **13d** was the most potent derivative, reducing the biofilm formation by 61%. In the case of *E. coli* AG100A, which lacked the AcrAB–TolC system, compounds **1**, **2**, and **12b** proved to be more effective, with compound **2** reducing biofilm formation by 65%. On the contrary, the antibiofilm activity shown by compounds **4** and **13a**–**13d** towards *E. coli* AG100 was greatly reduced towards the mutant *E. coli* AG100A strain.Regarding MRSA 272123, **13a** and **4** were the most effective derivatives, decreasing biofilm formation by 91%. The results for the compounds that showed the highest antibiofilm capacity are summarized in Figure 10. CCCP was used as a positive control.

#### 2.2.4. Preliminary Antibiotic Potentiation Study

A study was also performed to investigate possible antibiotic potentiation between the synthesized compounds **1**, **2**, and **12a**–**12d**, and a set of known antibiotics to which the isolates were resistant. The antibiotics used were vancomycin, for *E. faecalis* B3/101, oxacillin for *S. aureus* 66/1, and cefotaxime for *E. coli* SA/2. For a preliminary screening, the agar disk diffusion method, or Kirby–Bauer method, was performed according to CLSI guidelines [39]. Using this method, no antibiotic potentiation was observed between any of the tested antibiotics for any of the bacterial strains, since no significant increases were observed in the inhibition halos. However, since the agar disk diffusion method has many limitations, the compounds were also screened for synergism using the broth microdilution method. In this case, a fixed concentration of each compound was used (sub-MIC or 64 µg/mL when MIC > 64 µg/mL) in combination with a range of concentrations of the respective antibiotic. In the study of possible synergism with cefotaxime towards *E. coli* SA/2, the results showed that none of the tested compounds could reduce the MIC value of cefotaxime to at least half of its original value; therefore, it was concluded that no significant antibiotic potentiation was present. In the case of *E. faecalis* B3/101, compound **12a** reduced the MIC value obtained in this study from 516 to 256 µg/mL when tested at 16 µg/mL. Therefore, a possible antibiotic potentiation with this compound and vancomycin would have to be confirmed with further studies.

#### 2.2.5. Cytotoxicity Assays

The cytotoxicity of the compounds **1**, **2**, and **12a**–**12d** synthesized in this work was evaluated on the normal NIH/3T3 mouse fibroblast cell line (Table 4). Compounds **1** and **2** showed no toxicity (IC_50_ > 100 µM). However, compounds **12a**–**12d** showed some cytotoxicity, with IC_50_ values between 46 and 58 µM.

#### 2.2.6. Structure–Activity Relationship (SAR)

**Figure 11:**
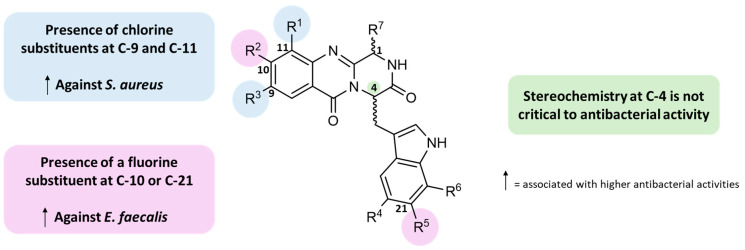
Proposed SAR regarding antibacterial activity for the synthesized derivatives.

By analyzing the biological activity screening results, it was possible to identify some characteristics from the structure–activity relationship (Figure 11).

Regarding the antibacterial activity of the synthesized compounds, when comparing the activity of fluorinated derivatives **12a** and **12b**, it was possible to observe that **12b** showed a lower MIC value against *E. faecalis* B3/101 and against *S. aureus* ATCC than **12a**. Furthermore, even though the MIC value against *S. aureus* 66/1 was the same for both compounds, it was possible to observe that **12b** led to a much more pronounced reduction in the number of colony forming units (CFUs) in the minimum bactericidal concentration (MBC) assay, suggesting that this compound had a stronger bactericidal effect. Therefore, those results indicate that the position of the fluorine substituent in the aromatic ring influences the potency of the antibacterial activity and it could also affect the mechanism of action. In the case of compounds **12a** and **12b**, the obtained MIC values for *S. aureus* were higher than those obtained for the derivatives with the chlorine substituents tested in this study (**12c** and **12d**), as well as higher than the chlorinated derivative (**4**, Figure 3) reported by Long et al. [26]. Therefore, chlorine substituents at C-18 and C-16 were considered more beneficial in terms of their activity against *S. aureus*. On the contrary, a fluorine substituent at C-17 or C-23, present in compounds **12b** and **12c**, was associated with lower MIC values against *E. faecalis* B3/101.

By comparing the obtained results with those reported by Long et al. [26] for similar compounds, it was also possible to further extend the structure–activity relationship study. Compounds **12c** and **12d** differ from compound **4** (Figure 3) in two main aspects: the stereochemistry at C-4 and the substituents in the indole moiety. By comparing the obtained results, it was possible to observe that the change in stereochemistry at C-4 did not lead to a complete loss of activity. However, the MIC values obtained for the sensitive strain of *S. aureus* ATCC 29213 were higher than those reported for compound **4** [26]. This decrease in the activity could either be related to the stereochemistry or to the halogen substituents in the indole moiety. Nonetheless, **12c** and **12d** were active against *E. faecalis*, particularly against VRE, while compound **4** (Figure 3) was not [26]. Therefore, the action spectrum of these compounds is broader than that of compound **4**, which is an advantage.

Regarding EPI activity, an isobutyl substituent at C-1 also seems to be associated with more potent activity, since the derivatives that had the most potent activities (**4**, **12a**–**12c**, **13a**–**13c**) all comprise this substituent. However, contrarily to the tendency observed for antibacterial activity, the presence of the two chlorine atoms at C-9 and C-11 does not seem to be required for EPI activity. Regarding the activity against *E. coli* AG100A, the presence of a fluorine atom at C-21 (compound **12c**) was associated with a higher RFI value, suggesting that the presence of this substituent might be relevant for the inhibition of efflux pumps in *E. coli*, which are different from the AcrAB–TolC system.

Finally, concerning antibiofilm capacity, it is interesting to note that compounds **12c** and **12d** did not show any relevant activity. These derivatives were the only compounds with an *S* configuration both at C-4 and at C-10, suggesting that the stereochemistry in these locations might affect antibiofilm activity. However, this influence would have to be confirmed with further studies.

#### 2.2.7. Docking Studies

The library of 11 derivatives (**1**, **2**, **4**, **12a**–**12d**, and **13a**–**13d**) was investigated in order to find their predicted binding affinities against relevant bacterial efflux pumps, the AcrAB–TolC efflux system from the RND family [40], and the MFS pump NorA [41]; their predicted affinity against these targets was ranked. NorA did not have a crystal structure deposited in the Protein Data Bank, and a homology model needed to be built. Reference compounds described as efflux pump inhibitors were used as positive controls.

Docking studies were performed based on the crystal structures of the AcrB (4DX5), AcrA (2F1M), and TolC (1EK9) portions of the AcrAB–TolC efflux system. For AcrB and AcrA, these studies were performed in two different sites: the substrate-binding site (SBS) and the hydrophobic trap (HT) for AcrB [42], and the helical hairpin (HH) and the lipoyl domain (LD) for AcrA [40]. For TolC, only the lysine residues that interact with the 3,3′-dithiobis(sulfosuccinimidyl propionate) (DTSSP) bifunctional crosslinker [40] were considered. For the NorA homology model, the sites used for docking of the compounds were the binding core region (BCR) and the cytoplasmic side (CS), as described in [43].

From the docking scores obtained (Table S3, Supplementary Materials), it can be noted that these compounds are predicted to present the most affinity towards AcrB. This affinity was comparable or higher than that of doxorubicin, a known AcrAB–TolC inhibitor, especially in the SBS. With regard to NorA, the compounds appeared to present a better predicted affinity towards the binding site present in the CS, with most of them being predicted to have a better binding affinity than reserpine.

**Figure 12:**
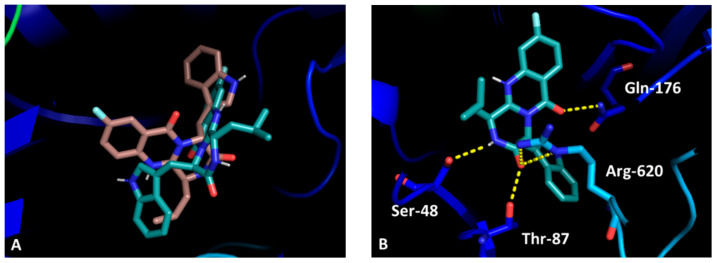

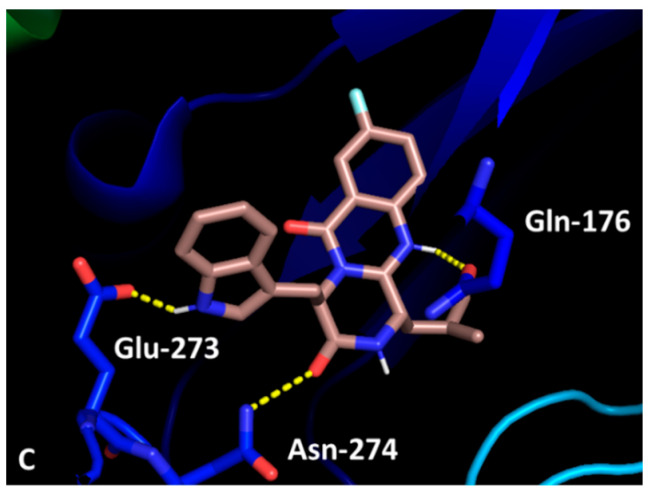
Molecular visualization of derivatives **12a** and **12b** in the SBS of AcrB. (**A**) General view of compounds **12a** (blue) and **12b** (pink); (**B**) Interaction between compound **12a** and the SBS; (**C**) Interaction between compound **12b** and the SBS.

Molecular visualization was performed for the compounds that were more effective against the efflux of ethidium bromide (Figure 9). In the case of the Gram-negative model, compounds **12a** (Figure 6) and **12b** (Figure 6) are likely to inhibit the AcrAB–TolC efflux system, whereas **12c** (Figure 6) probably inhibits efflux systems, other than the aforementioned. As such, the latter was not visualized.

Upon first glance, it could be noted that the most favorable poses of the compounds were opposite one another, as can be seen in Figure 12A. Compound **12a** was predicted to establish hydrogen interactions with the polar amino acids Ser-48, Thr-87, Gln-176, and the positively charged Arg-620 (Figure 12B). On the other hand, compound **12b** appeared to establish interactions with the polar Gln-176 and Asn-274, and the negatively charged Glu-273. These interactions were in accordance with what was predicted for other compounds with a tricyclic scaffold, such as xanthones and thioxanthones [34,44]. Additionally, docking studies with known AcrB inhibitors, such as minocycline and doxorubicin, have shown predicted interactions with Gln-176, suggesting the importance of this residue in the interaction with inhibitors [42].

Molecular visualization of derivatives **4**, **12b**, **12c**, **13a**, and **13b** can be found in the Supplementary Information (Figure S52).

## 3. Materials and Methods

### 3.1. Chemistry

#### 3.1.1. Materials and Methods

All reagents and solvents used were purchased from Sigma-Aldrich (Sigma-Aldrich Co. Ltd., Gillinghan, UK), TCI (Tokyo, Japan), and Fluorochem (Hadfield, UK) and they did not undergo any further purification process. Solvents were evaporated with a rotary evaporator under reduced pressure using a Buchi Waterbath B-480. TLC was used to monitor all reactions and was carried out on precoated plates of 0.2 mm thickness using Merck silica gel 60 (GF_254_). UV light at 254 and 365 nm, and in some cases, a solution of ferric (III) chloride and a solution of green bromocresol as well as an iodine chamber, were used to visualize the chromatograms. The purification processes of the synthesized compounds were performed using flash column chromatography using Merck silica gel 60 (0.040–0.063 mm, Merck, Darmstadt, Germany) and preparative TLC using Merck silica gel HPLC60 RP-18 (GF_254_) purchased from Merck (Germany). In some cases, the compounds were not purified to avoid a loss of yield and they were used immediately in the next reaction. Therefore, in those cases the yield could not be calculated. Purity of the synthesized compounds was assessed by Doctor Sara Cravo at LQOF by HPLC-DAD. The HPLC system consisted of a Thermo Scientific SpectraSystem P4000 pump equipped with a degasser, a Thermo Scientific SpectraSystem AS3000 autosampler fitted with a maximum volume 100 µL loop, and a Thermo Scientific SpectraSystem UV8000 DAD detector (Waltham, MA, USA). Data acquisition was performed using ChromQuest 5.0 software, version 3.2.1. The column used in this study was ACE–C18 (150 × 4.6 mm I.D., particle size 5 µm) manufactured by Advanced Chromatography Technologies Ltd. (Aberdeen, Scotland, UK). The mobile phase composition was water and acetonitrile (55:45 *v*/*v*; 0.1% CH_3_CO_2_H) and all solvents were HPLC-grade obtained from Merck Life Science S.L.U. (Darmstadt, Germany). The flow rate was 1.0 mL/min and the UV detection wavelength was set at 254 nm. Analyses were performed at room temperature in an isocratic mode in a 30 min run. The peak purity index was determined by total peak UV–Vis spectra between 210 and 800 nm with a step of 4 nm. Melting points (mp) were measured using a Köfler microscope (Wagner and Munz, Munich, Germany) and the values shown correspond to the mean value obtained from three independent experiments. For the characterization of the synthesized compounds, NMR spectra were performed by the Department of Chemistry of the University of Aveiro, at room temperature, utilizing a Bruker Advance 300 spectrometer (300.13 MHz for 1 h and 75.47 MHz for ^13^C, Bruker Biosciences Corporation, Billerica, MA, USA). Carbons were assigned using HSQC and HMBC spectra. Optical rotation was measured at 25 °C using the ADP 410 polarimeter (Bellingham + Stanley Ltd., Tunbridge Wells, Kent, UK), using the emission wavelength of a sodium lamp. Concentrations are given in g per 100 mL. HRESIMS analyses were performed at CIIMAR, Matosinhos, and they were obtained on a Q Exactive Focus Hybrid Quadrupole Orbitrap Mass Spectrometer (Thermo Fisher Scientific), controlled by Q Exactive Focus (Exactive Series) 2.9 and Thermo Scientific Xcalibur 4.1.31.9 software. HRESIMS data were obtained in full scan positive and negative mode with a scan range of *m*/*z* 100–1500, with a capillary voltage of HESI set to −3.5 kV and the capillary temperature to 263 °C. The sheath gas flow rate was set to 50 units. Enantiomeric excess analysis was performed with an HPLC system consisting of a Shimadzu LC-20AD pump, equipped with a Shimadzu DGV-20A5 degasser, a Rheodyne 7725i injector fitted with a 20 µL loop, and a SPD-M20A DAD detector (Kyoto, Japan). Data were acquired using Shimadzu LCMS Lab Solutions software, version 3.50 SP2 (Kyoto, Japan). The column used in this study was the Phenomenex–Lux^®^ 5 µm Amylose-1 (250 × 4.6 mm I.D., particle size 5 µm) manufactured by Phenomenex, Inc. (Torrance, CA, USA). The mobile phase composition was n-hexane and ethanol (7:3 *v/v*); all were HPLC-grade solvents obtained from Carlo Erba, Reagents S.r.l. (Val de Reuil, France). The flow rate was 1.0 mL/min and the UV detection wavelength was 254 nm. Analyses were performed at room temperature in an isocratic mode in a 20 to 75 min run.

#### 3.1.2. Synthesis of Tryptophan Methyl Esters Hydrochlorides **7b**–**7c**

##### General Procedure

The appropriate tryptophan derivative (1 equiv.) was dissolved in dry MeOH (1.4 mL) and SOCl_2_ (1.75 equiv.) was added dropwise at 0 °C. The mixture was stirred at room temperature overnight under a nitrogen atmosphere, and then the solvent was evaporated under reduced pressure. Residue was washed with EtOAc and the resulting solid was filtered under vacuum. Compounds were used in the next reaction without further purification.

##### Synthesis of Methyl (*S*)-2-Amino-3-(6-fluoro-1*H*-indol-3-yl)propanoate (**7b**)

(*S*)-2-amino-3-(6-fluoro-1*H*-indol-3-yl)propanoic acid (1 equiv., 100 mg, 0.454 mmol) was dissolved in dry MeOH (1.4 mL) and reacted with thionyl chloride (1.75 equiv., 58 µL, 0.795 mmol) under a nitrogen atmosphere to yield **7b** as an off-white solid.

##### Synthesis of Methyl (*S*)-2-Amino-3-(7-chloro-1*H*-indol-3-yl)propanoate (**7c**)

(*S*)-2-amino-3-(7-chloro-1*H*-indol-3-yl)propanoic acid (1 equiv., 125 mg, 0.523 mmol) was dissolved in dry MeOH (1.7 mL) and reacted with thionyl chloride (1.75 equiv., 66 µL, 0.917 mmol) under a nitrogen atmosphere to yield **7c** as an off-white solid.

#### 3.1.3. Synthesis of Linear Dipeptides **8a**–**8e**

##### General Procedure

To a solution of the appropriate anthranilic acid derivative (1 equiv., 14.58 mmol) and TBTU (1.2 equiv.) in acetonitrile, Et_3_N (2 equiv.) and the appropriate tryptophan methyl ester (1.14 equiv.) were added at room temperature. The reaction mixture was stirred for 5 h and monitored with TLC. The solvent was evaporated under reduced pressure. The residue was dissolved in CH_2_Cl_2_ and washed with HCl (1M, 1×) and water (1×). The organic phase was collected and the aqueous phase was extracted with CH_2_Cl_2_ (3×). The combined organic phases were dried over anhydrous Na_2_SO_4_, filtered, and concentrated under reduced pressure.

##### Synthesis of Methyl (2-Aminobenzoyl)-D-tryptophanate (**8a**)

Anthranilic acid (1 equiv., 2.00 g, 14.58 mmol) and D-tryptophan methyl ester (1.14 equiv., 3.63 g, 16 mmol) were coupled in the presence of TBTU (1.2 equiv., 6.41 g, 19.96 mmol) and Et_3_N (2.9 equiv., 5.80 mL, 41.6 mmol) in acetonitrile (140 mL) for 5 h.

Purification was performed by flash column chromatography using a mixture of 1% MeOH in CH_2_Cl_2_ as the mobile phase. Dipeptide **8a** was obtained as a white solid in a 74% yield.

Methyl (2-aminobenzoyl)-D-tryptophanate (**8a**): White solid (3.7 g, 74% yield); ^1^H NMR (300 MHz, CDCl_3_): δ = 8.17 (br s, 1H, NH indole), 7.57 (d, *J* = 7.9 Hz, 1H, H-7), 7.35 (d, *J* = 8.0 Hz, 1H, H-15), 7.22–7.16 (m, 3H, H-4, H-5, H-17), 7.10 (ddd, *J* = 8.0, 7.0, 1.1 Hz, 1H, H-6), 7.00 (d, *J* = 2.4 Hz, 1H, H-1), 6.65 (dd, *J* = 8.7, 1.2 Hz, 1H, H-18), 6.61–6.53 (m, 2H, H-16, NH amide), 5.50 (br s, 2H, NH_2_), 5.08 (dt, *J* = 7.5, 5.3 Hz, 1H, H-11), 3.72 (s, 3H, OCH_3_), 3.43 (d, *J* = 5.4 Hz, 2H, H-10, H-10′). ^13^C NMR (75 MHz, CDCl3) δ 172.66 (C-21), 168.90 (C-13), 148.89 (C-19), 136.23 (C-3), 132.68 (C-17), 127.66 (C-8), 122.96 (C-1), 122.43 (C-5), 119.87 (C-6), 118.78 (C-7), 117.42 (C-16), 116.77 (C-18), 115.42 (C-14), 111.41 (C-4), 110.20 (C-9), 53.22 (C-11), 52.58 (OCH_3_), 27.79 (C-10).(According to the literature [27,29]).

##### Synthesis of Dipeptide Methyl (2-Amino-4-fluorobenzoyl)-D-tryptophanate (**8b**)

2-Amino-4-fluorobenzoic acid (1 equiv., 250 mg, 1.61 mmol) and D-tryptophan methyl ester (1.14 equiv., 401 mg, 1.84 mmol) were coupled in the presence of TBTU (1.20 equiv., 621 mg, 1.93 mmol) and Et_3_N (2 equiv., 449 µL, 3.22 mmol) in acetonitrile (15.5 mL). Purification by flash column chromatography with silica gel using MeOH:CH_2_Cl_2_ gradient (0:100 to 4:96) as the mobile phase allowed dipeptide **8b** to be obtained as a white solid in a 97% yield. In a second attempt at this reaction, it was also possible to purify the compound by insolubilization in MeOH.

Methyl (2-amino-4-fluorobenzoyl)-D-tryptophanate (**8b**): White solid (554 mg, 97% yield), ^1^H-NMR (300 MHz, CDCl_3_) δ 8.15 (br s, 1H, NH indole), 7.55 (dd, *J* = 8.1, 1.1 Hz, 1H, H-7), 7.36 (dt, *J* = 8.2, 1.0 Hz, 1H, H-4), 7.20 (ddd, *J* = 8.1, 7.0, 1.2 Hz, 1H, H-5), 7.15–7.11 (m, 1H, H-15), 7.10–7.07 (m, 1H, H-6), 7.01 (d, *J* = 2.4 Hz, 1H, H-1), 6.48 (d, *J* = 7.5 Hz, 1H, NH amide), 6.31 (dd, *J* = 10.9, 2.5 Hz, 1H, H-18), 6.27–6.20 (m, 1H, H-16), 5.71 (s, 2H, NH_2_), 5.06 (dt, *J* = 7.5, 5.3 Hz, 1H, H-11), 3.73 (s, 3H, OCH_3_), 3.49–3.35 (m, 2H, H-10, H-10′). ^13^C-NMR (75 MHz, CDCl_3_) δ 172.50 (C-21), 168.09 (C-13), 165.45 (d, *J* = 249.7 Hz, C-17), 151.22 (d, *J* = 12.0 Hz, C-19), 136.11 (C-3), 129.77 (d, *J* = 11.25 Hz, C-15), 127.58 (C-8), 122.77 (C-1), 122.39 (C-5), 119.81 (C-6), 118.63 (C-7), 111.53 (C-14), 111.33 (C-4), 110.08 (C-9), 104.01 (d, *J* = 22.7 Hz, C-16), 102.90 (d, *J* = 24.2 Hz, C-18), 53.15 (C-11), 52.49 (OCH_3_), 27.61 (C-10).

##### Synthesis of Dipeptide Methyl (2-Amino-5-fluorobenzoyl)-D-tryptophanate (**8c**)

2-Amino-5-fluorobenzoic acid (1 equiv., 250 mg, 1.61 mmol) and D-tryptophan methyl ester (1.14 equiv., 401 mg, 1.84 mmol) were coupled in the presence of TBTU (1.20 equiv., 621 mg, 1.93 mmol) and Et_3_N (2 equiv., 449 µL, 3.22 mmol) in acetonitrile (15.5 mL). Purification by flash column chromatography with silica gel using MeOH:CH_2_Cl_2_ gradient (0:100 to 4:96) as the mobile phase allowed dipeptide **8c** to be obtained as a white solid in a 54% yield. In a second attempt at this reaction, it was also possible to purify the compound by insolubilization in MeOH.

Methyl (2-amino-5-fluorobenzoyl)-D-tryptophanate (**8c**): White solid (309 mg, 54% yield) ^1^H NMR (300 MHz, CDCl_3_) δ 8.15 (s, 1H, NH indole), 7.56 (d, *J* = 7.9 Hz, 1H, H-7), 7.37 (d, *J* = 8.1 Hz, 1H, H-4), 7.20 (ddd, 1H, *J* = 8.2, 7.0, 1.2 Hz, H-5), 7.11 (ddd, *J* = 8.0, 7.0, 1.1 Hz, 1H, H-6), 7.02 (d, *J* = 2.2 Hz, 1H, H-1), 6.94 (ddd, *J* = 8.9, 7.8, 2.9 Hz, 1H, H-17), 6.88 (dd, *J* = 9.2, 2.9 Hz, 1H, H-15), 6.63 (dd, *J* = 8.9, 4.6 Hz, 1H, H-18), 6.56 (br d, *J* = 7.4 Hz, 1H, NH amide), 5.06 (dt, *J* = 7.6, 5.3 Hz, 1H, H-11), 3.73 (s, 3H, OCH_3_), 3.44 (dd, *J* = 3.5, 12.9 Hz, 1H, H-10), 3.40 (dd, *J* = 13.3, 3.9 Hz, 1H, H-10′). ^13^C NMR (75 MHz, CDCl_3_) δ 172.33 (C-21), 167.68 (C-13), 154.51 (d, *J* = 236.4 Hz, C-16), 144.57 (d, *J* = 5.1 Hz, C-19), 136.12 (C-3), 127.52 (C-8), 122.76 (C-1), 122.47 (C-5), 119.88 (C-6), 119.93 (d, *J* = 22.9 Hz, C-17), 118.69 (d, *J* = 2.3 Hz, C-18), 118.55 (C-7), 116.00 (d, *J* = 2.7 Hz, C-14) 113.39 (d, *J* = 23.10 Hz, C-15), 111.37 (C-4), 109.98 (C-9), 53.20 (C-11), 52.54 (OCH_3_), 27.56 (C-10).

##### Synthesis of Dipeptide Methyl (*S*)-2-(2-Amino-3,5-dichlorobenzamido)-3-(6-fluoro-1*H*-indol-3-yl)propanoate (**8d**)

2-Amino-3,5-dichlorobenzoic acid (1 equiv., 81 mg, 0.394 mmol) and (*S*)-2-amino-3-(6-fluoro-1*H*-indol-3-yl)propanoate (**7b**) (1.14 equiv., 106 mg, 0.450 mmol) were coupled in the presence of TBTU (1.20 equiv., 152 mg, 0.472 mmol) and Et_3_N (2 equiv., 109 µL, 0.787 mmol) in acetonitrile (3.3 mL). Purification by preparative TLC using silica plates and CH_2_Cl_2_:EtOAc (80:20) as the mobile phase allowed dipeptide **8d** to be obtained in a 32% yield as a light-yellow solid.

(*S*)-2-(2-amino-3,5-dichlorobenzamido)-3-(6-fluoro-1*H*-indol-3-yl)propanoate (**8d**): Off-white solid (53 mg, 32% yield) ^1^H NMR (300 MHz, DMSO-d_6_) δ 10.95 (d, *J* = 2.2 Hz, 1H, NH indole), 8.92 (d, *J* = 7.4 Hz, 1H, NH amide), 7.57 (d, *J* = 2.5 Hz, 1H, H-15), 7.54 (d, *J* = 2.4 Hz, 1H, H-17), 7.20 (d, *J* = 2.3 Hz, 1H, H-1), 7.13 (d, *J* = 2.3 Hz, 1H, H-7), 7.10 (d, *J* = 2.3 Hz, 1H, H-4), 6.86 (ddd, *J* = 9.8, 8.6, 2.4 Hz, 1H, H-6), 6.56 (s, 1H, NH_2_), 4.67–4.58 (m, 1H, H-11), 3.64 (s, 1H, OCH_3_), 3.30–3.12 (m, 2H, H-10, H-10′). ^13^C NMR (75 MHz, DMSO-d_6_) δ 172.70 (C-21), 167.64 (C-13), 159.28, (d, *J* = 233.6 Hz, C-5), 144.73 (C-19), 136.33 (d, *J* = 12.7 Hz, C-3), 131.68 (C-17),127.47 (C-15), 124.67 (C-1), 124.37 (C-8), 120.13 (C-18), 119.49, (d, *J* = 10.1 Hz, C-7), 118.20 (C-16), 116.61 (C-14), 110.62 (C-9), 107.41 (d, *J* = 24.1 Hz, C-6), 97.91 (d, *J* = 24.9 Hz, C-4), 54.16 (C-11), 52.50 (OCH_3_), 26.75 (C-10).

##### Synthesis of Dipeptide Methyl (*S*)-2-(2-Amino-3,5-dichlorobenzamido)-3-(7-chloro-1*H*-indol-3-yl)propanoate (**8e**)

2-Amino-3,5-dichlorobenzoic acid (1 equiv., 94.4 mg, 0.458 mmol) and methyl (*S*)-2-amino-3-(7-chloro-1*H*-indol-3-yl)propanoate (**7c**) (1.14 equiv., 132 mg, 0.524 mmol) were coupled in the presence of TBTU (1.20 equiv., 176.55 mg, 0.550 mmol) and Et_3_N (2 equiv., 128 µL, 0.916 mmol) in acetonitrile (3.9 mL). Purification by preparative TLC using silica plates and CH_2_Cl_2_:EtOAc (80:20) as the mobile phase allowed dipeptide **8e** to be obtained in a 58% yield as a light-yellow solid.

Methyl (*S*)-2-(2-amino-3,5-dichlorobenzamido)-3-(7-chloro-1*H*-indol-3-yl)propanoate (**8e**): White solid (116 mg, 58% yield) ^1^H NMR (300 MHz, DMSO-d_6_) δ 11.27 (d, *J* = 2.5 Hz, 1H, NH indole), 8.94 (d, *J* = 7.4 Hz, 1H, NH amide), 7.60–7.50 (m, 3H, H-15, H-17, H-7), 7.29 (d, *J* = 2.4 Hz, 1H, H-1), 7.16 (d, *J* = 7.5 Hz, 1H, H-5), 7.01 (t, *J* = 7.7 Hz, 1H, H-6), 6.57 (s, 2H, NH_2_), 4.72–4.57 (m, 1H, H-11), 3.64 (s, 3H, OCH_3_), 3.29 (dd, *J* = 9.4, 5.7 Hz, 2H, H-10, H-10′). ^13^C NMR (75 MHz, DMSO-d_6_) 172.59 (C-21), 167.62 (C-13), 144.79 (C-19), 133.30 (C-3), 131.70 (C-17), 129.51 (C-8), 127.46 (C-15), 125.48 (C-1), 121.04 (C-5), 120.14 (C-18), 120.01 (C-6), 118.17 (C-16), 117.66 (C-7), 116.54 (C-14), 116.36 (C-4), 111.82 (C-9), 54.07 (C-11), 52.50 (OCH_3_), 26.76 (C-10).

#### 3.1.4. Synthesis of Acid Chlorides **10a**–**10c**

##### General Procedure

The appropriate Fmoc-protected amino acid (1 equiv.) was suspended in dry CH_2_Cl_2_ and SOCl_2_ (13.8 equiv.) was slowly added. The mixture was sonicated at room temperature for 30 min under a nitrogen atmosphere. Excess SOCl_2_ was removed and acid chlorides **10a**–**10c** were used immediately in the next reaction without further purification.

##### Synthesis of Fmoc-Gly-Cl (**10a**)

Fmoc-Gly-OH (1 equiv., 500 mg, 1.68 mmol) was treated with SOCl_2_ (13.8 equiv., 1.7 mL, 23.21 mmol) in dry CH_2_Cl_2_ (8 mL) to yield Fmoc-Gly-Cl as a white solid.

##### Synthesis of Fmoc-L-Ala-Cl (**10b**)

Fmoc-L-Ala-OH (1 equiv., 500 mg, 1.61 mmol) was treated with SOCl_2_ (13.8 equiv., 1.6 mL, 22.16 mmol) in dry CH_2_Cl_2_ (8 mL) to yield Fmoc-L-Ala-Cl as a white solid.

##### Synthesis of Fmoc-L-Leu-Cl (**10c**)

Fmoc-L-Leu-OH (1 equiv., 300 mg, 0.849 mmol) was treated with SOCl_2_ (13.8 equiv., 0.855 mL, 11.71 mmol) in dry CH_2_Cl_2_ (4 mL) to yield Fmoc-L-Leu-Cl as a light-yellow oil.

#### 3.1.5. Synthesis of Linear Tripeptides **11a**–**11f**

##### General Procedure

A solution of the appropriate dipeptide (1 equiv.) and the appropriate acid chloride (1.2 equiv.) in dry CH_2_Cl_2_ was stirred at room temperature under a nitrogen atmosphere for 15–30 min. Then, a solution of Na_2_CO_3_ (1M) was added and the reaction mixture was stirred at room temperature and monitored with TLC. Afterwards, the mixture was extracted with CH_2_Cl_2_ (4×) and the organic phase was washed with brine (1×), dried over anhydrous Na_2_SO_4_, filtered, and the solvent was evaporated under reduced pressure.

##### Synthesis of Methyl (2-(2-((((9*H*-Fluoren-9-yl)methoxy)carbonyl)amino) acetamido)benzoyl)-D-tryptophanate (**11a**)

Dipeptide **8a** (1 equiv., 437 mg, 1.30 mmol) was coupled with Fmoc-Gly-Cl (**10a**, 1.2 equiv., 490 mg, 1.55 mmol) in dry CH_2_Cl_2_ (30 mL) for 15 min. Afterwards, a solution of Na_2_CO_3_ (1M, 20 mL) was added and mixture was stirred for 12 h. Work-up of the reaction was carried out according to the general procedure. Tripeptide **11a** was obtained as a white solid and used in the next reaction without further purification.

##### Synthesis of Methyl (2-((S)-2-((((9*H*-Fluoren-9-yl)methoxy)carbonyl)amino)propanamido)benzoyl)-D-tryptophanate (**11b**)

Dipeptide **8a** (1 equiv., 426 mg, 1.26 mmol) was coupled with Fmoc-L-Ala-Cl (**10b**, 1.2 equiv., 500 mg, 1.52 mmol) in dry CH_2_Cl_2_ (30 mL). Afterwards, a solution of Na_2_CO_3_ (1M, 20 mL) was added and mixture was stirred for 1 h. Work-up of the reaction was carried out according to the described general procedure. Purification by flash column chromatography using MeOH:CH_2_Cl_2_ gradient (0:100 to 10:90) as the mobile phase allowed tripeptide **11b** to be obtained as a white solid in a 69% yield.

Methyl (2-((*S*)-2-((((9*H*-fluoren-9-yl)methoxy)carbonyl)amino)propanamido)-benzoyl)-D-tryptophanate (**11b**): White solid (550 mg, 69% yield) ^1^H NMR (300 MHz, CDCl_3_): δ = 11.22 (s, 1H, H, NH-20), 8.54 (d, *J* = 8.4 Hz, 1H, H-Ar), 8.43 (br s, 1H, NH indole), 7.77 (d, *J* = 7.4 Hz, 2H, H-Ar), 7.67 (d, *J* = 7.4 Hz, 1H, H-Ar), 7.62 (d, *J* = 7.4 Hz, 1H, H-Ar), 7.52–7.27 (m, 8H, H-Ar), 7.15 (t, *J* = 7.5 Hz, 1H, H-Ar), 7.06 (d, *J* = 7.2 Hz, 1H, H-Ar), 7.01 (d, *J* = 8.2 Hz, 1H, H-Ar), 6.97 (br s, 1H, NH-12), 6.71 (d, *J* = 8.0 Hz, 1H, H-1), 5.53 (d, *J* = 7.3 Hz, 1H, NH-Fmoc), 5.04 (q, *J* = 6.4 Hz, 1H, H-11), 4.54–4.20 (m, 3H, Fmoc-CH, Fmoc-CH_2_), 4.26 (t, *J* = 7.0 Hz, 1H, H-22), 3.71 (s, 3H, OCH_3_), 3.43 (dd, *J* = 14.8, 4.8 Hz, 1H, H-10), 3.24 (dd, *J* = 14.8, 6.4 Hz, 1H, H-10′), 1.48 (d, *J* = 7.1 Hz, 3H, CH_3_). (According to the literature [27]).

##### Synthesis of Methyl (2-((*S*)-2-((((9*H*-Fluoren-9-yl)methoxy)carbonyl)amino)-4-methylpentanamido)-4-fluorobenzoyl)-D-tryptophanate (**11c**)

Dipeptide **8b** (1 equiv., 180 mg, 0.506 mmol) was coupled with Fmoc-L-Leu-Cl (**10c**, 1.20 equiv., 226 mg, 0.608 mmol), in dry CH_2_Cl_2_ (12 mL) for 15 min. Afterwards a solution of Na_2_CO_3_ (1M, 8 mL) was added and the mixture was stirred for 1 h. Work-up of the reaction was carried out according to the described general procedure. Purification by preparative TLC using silica plates and CH_2_Cl_2_:EtOAc (80:20) as the mobile phase allowed **11c** to be obtained as a white solid in a 53% yield.

Methyl (2-((*S*)-2-((((9*H*-fluoren-9-yl)methoxy)carbonyl)amino)-4-methylpentanamido)-4-fluorobenzoyl)-D-tryptophanate (**11c**): White solid (185 mg, 53% yield), ^1^H-NMR (300 MHz, CDCl_3_) δ 11.47 (s, 1H, NH indole), 8.47 (s, 1H, NH amide (H-2)), 8.41 (dd, *J* = 11.6, 2.6 Hz, 1H, H-Ar), 7.78 (d, *J* = 7.5 Hz, 2H, H-Ar), 7.68 (d, *J* = 7.5 Hz, 1H, H-Ar), 7.62 (d, *J* = 7.4 Hz, 1H, H-Ar), 7.48 (d, *J* = 7.9 Hz, 1H, H-Ar), 7.44–7.20 (m, 6H H-Ar), 7.15 (t, *J* = 7.5 Hz, 1H, H-Ar), 7.04 (ddd, *J* = 8.0, 7.0, 1.1 Hz, 1H, H-Ar), 6.96 (d, *J* = 2.4 Hz, 1H, H-1), 6.67 (ddd, *J* = 8.8, 7.5, 2.6 Hz, 1H, H-Ar), 6.59 (d, *J* = 8.1 Hz, 1H, NH amide (H-12)), 5.33 (d, *J* = 8.2 Hz, 1H, NH Fmoc), 5.09–4.96 (m, 1H, H-11), 4.50 (dd, *J* = 10.6, 6.9 Hz, 1H, Fmoc-CH_2_), 4.40 (dd, *J* = 10.4, 7.3 Hz, 1H, Fmoc-CH_2_), 4.36–4.22 (m, 2H, Fmoc-CH, H-22), 3.70 (s, 3H, OCH_3_), 3.41 (dd, *J* = 14.9, 4.8 Hz, 1H, H-10), 3.20 (dd, *J* = 14.8, 6.5 Hz, 1H, H-10′), 1.73 (ddd, *J* = 15.0, 11.3, 5.5 Hz, 2H, H-23, H-23′), 1.58 (d, *J* = 8.4 Hz, 1H, H-24), 0.97 (d, *J* = 6.0 Hz, 6H, CH_3_, CH_3_′). ^13^C NMR (75 MHz, CDCl_3_) δ 172.02 (C-28), 171.46 (C-21), 167.47 (C-13), 165.36 (d, *J* = 206.4 Hz, C-17), 156.26 (Fmoc C=O), 143.87 (d, *J* = 33.1 Hz, C-Ar) 141.29 (C-Ar), 136.13 (C-3), 127.72 (C-Ar), 127.50 (C-Ar), 127.08 (C-Ar), 125.22 (d, *J* = 8.0 Hz, C-Ar), 122.79 (C-1), 122.31 (C-Ar), 119.96 (C-Ar), 119.70 (C-Ar), 118.34 (C-Ar), 116.10 (d, *J* = 5.0 Hz, C-Ar), 111.41 (C-Ar), 110.19 (C-9), 109.72 (C-8), 108.52 (d, *J* = 27.9 Hz, C-Ar), 67.25 (Fmoc-CH_2_), 54.94 (C-22), 53.15 (C-11), 52.58 (OCH_3_), 47.26 (Fmoc-CH), 41.98 (C-23), 28.05 (C-10), 24.89 (C-24), 22.96 (CH_3_), 21.89 (CH_3_′).

##### Synthesis of Methyl (2-((*S*)-2-((((9*H*-Fluoren-9-yl)methoxy)carbonyl)amino)-4-methylpentanamido)-5-fluorobenzoyl)-D-tryptophanate (**11d**)

Dipeptide **8c** (1 equiv., 175 mg, 0.492 mmol) was coupled with Fmoc-L-Leu-Cl (**10c**, 1.20 equiv., 220 mg, 0.591 mmol), in dry CH_2_Cl_2_ (11.7 mL) for 15 min. Afterwards a solution of Na_2_CO_3_ (1M, 8 mL) was added and the mixture was stirred for 1 h. Work-up of the reaction was carried out according to the described general procedure. The compound was used in the next reaction without further purification.

##### Synthesis of Methyl (*S*)-2-(2-((*S*)-2-((((9*H*-Fluoren-9-yl)methoxy)carbonyl)amino)-4-methylpentanamido)-3,5-dichlorobenzamido)-3-(6-fluoro-1*H*-indol-3-yl)propanoate (**11e**)

Dipeptide **8d** (1 equiv., 50 mg, 0.118 mmol) was coupled with Fmoc-L-Leu-Cl (**10c**, 1.20 equiv., 52.6 mg, 0.141 mmol), in dry CH_2_Cl_2_ (2.8 mL) for 30 min. The reaction was neutralized with Na_2_CO_3_ (1M) and the work-up was carried out according to the described general procedure. The obtained compound was used in the next reaction without further purification.

##### Synthesis of Tripeptide Methyl (*S*)-2-(2-((*S*)-2-((((9*H*-Fluoren-9-yl)methoxy)carbonyl)amino)-4-methylpentanamido)-3,5-dichlorobenzamido)-3-(7-chloro-1H-indol-3-yl)propanoate (**11f**)

Dipeptide **8e** (1 equiv., 116 mg, 0.263 mmol) was coupled with Fmoc-L-Leu-Cl (**10c**, 1.20 equiv., 117 mg, 0.316 mmol), in dry CH_2_Cl_2_ (6.2 mL) for 30 min. The reaction was neutralized with Na_2_CO_3_ (1M) and the work-up was carried out according to the described general procedure. The obtained compound was used in the next reaction without further purification.

#### 3.1.6. Synthesis of Derivatives **1**, **2** and **12a**–**12d**

##### Synthesis of (*R*)-4-((1*H*-Indol-3-yl)methyl)-1,2-dihydro-6*H*-pyrazino[2,1-*b*]quinazoline-3,6(4*H*)-dione (**1**, **Glyantrypine**)––Method A

To a solution of tripeptide **11a** (1 equiv., 390 mg, 0.601 mmol), Ph_3_P (5 equiv., 829 mg, 3.16 mmol) and I_2_ (4.9 equiv., 785 mg, 3.10 mmol) in dry CH_2_Cl_2_ (40 mL) *N*,*N*-diisopropylethylamine (10.1 equiv., 1.06 mL, 6.08 mmol) were added. The reaction mixture was stirred at room temperature for 5 h under a nitrogen atmosphere. Then, an aqueous solution of Na_2_CO_3_ (1M, 80 mL) was added to quench the reaction. The aqueous phase was extracted with CH_2_Cl_2_ (3 × 100 mL) and the organic phase was washed with brine (1 × 100 mL). The organic phase was dried over Na_2_SO_4_, filtered, and concentrated under reduced pressure. Hexane (3 × 200 mL) was added to remove excess Ph_3_P. After filtration, the residue was stirred in a solution of CH_2_Cl_2_:piperidine (8:1.6 mL) for 20 min to remove the Fmoc protecting group. The solvent was evaporated under reduced pressure and the residue was subsequently stirred in reflux in acetonitrile (10 mL) overnight. Purification was carried out by preparative TLC using EtOAc:CH_2_Cl_2_:MeOH (50:45:5) as the mobile phase. A yellow oil was obtained that was further purified by crystallization in methanol to yield glyantrypine (**1**) as small white crystals (25% yield).

(*R*)-4-((1*H*-indol-3-yl)methyl)-1,2-dihydro-6*H*-pyrazino[2,1-*b*]quinazoline-3,6(4*H*)-dione (**1**, **Glyantrypine**): White crystals (52 mg, 25% yield). m.p. 159.7–162.3 °C (lit. 159–161 °C [27]). [α_D_^25^] = −390 (*c* 0.033, THF) (lit. [α_D_^30^] −522 (c 0.24, CHCl_3_) [27]). ^1^H NMR (500 MHz, DMSO-*d*_6_) δ 10.95 (br s, 1H, NH indole), 8.34 (d, *J* = 4.3 Hz, 1H, NH Amide), 8.22 (d, *J* = 7.9 Hz, 1H, H-8), 7.84 (td, *J* = 7.6, 1.5 Hz, 1H, H-10), 7.57 (ddd, *J* = 9.3, 7.9, 1.6 Hz, 2H, H-11, H-9), 7.32 (d, *J* = 8.1 Hz, 1H, H-23), 7.27 (d, *J* = 7.9 Hz, 1H, H-20), 7.02 (t, *J* = 7.5 Hz, 1H, H-21), 6.88 (d, *J* = 2.2 Hz, 1H, H-17), 6.79 (t, *J* = 7.5 Hz, 1H, H-22), 5.28 (t, *J* = 4.8 Hz, 1H, H-4), 3.82 (dd, *J* = 17.0, 4.5 Hz, 1H, H-1), 3.49–3.40 (m, 2H, H-15, H-15′), 3.08 (d, *J* = 17.0 Hz, 1H, H-1′) ^13^C NMR (126 MHz, DMSO-d_6_) δ 167.64 (C-3), 159.97 (C-6), 149.33 (C-14), 147.03 (C-12), 136.03 (C-19), 134.77 (C-10), 127.19 (C-24), 126.77 (C-9), 126.65 (C-11), 126.34 (C-8), 124.45 (C-17), 121.25 (C-21), 119.91 (C-7), 118.66 (C-22), 117.79 (C-23), 111.46 (C-20), 107.80 (C-16), 56.49 (C-4), 43.82 (C-1), 39.52 (C-15). (According to the literature [27]) HPLC-DAD: Peak purity = 97.8%; Chromatographic Purity = 100%.

##### General Procedure––Method C

To a solution of the appropriate tripeptide (1 equiv.), Ph_3_P (5 equiv.) and I_2_ (4.9 equiv.) in dry CH_2_Cl_2_ (40 mL) *N*,*N*-diisopropylethylamine (10.1 equiv.) were added. The reaction mixture was stirred at room temperature for 5 h under a nitrogen atmosphere. Then, an aqueous solution of Na_2_CO_3_ (1M) was added to quench the reaction. The aqueous phase was extracted with CH_2_Cl_2_ (3×) and the organic phase was washed with brine (1×). The organic phase was dried over Na_2_SO_4_, filtered, and concentrated under reduced pressure. Hexane (3×) was added to remove excess Ph_3_P. The precipitate was filtered under vacuum and dissolved in EtOAc. Piperidine (25.84 equiv.) was added and the mixture was stirred at room temperature for 1 h. The solvent was evaporated under reduced pressure. The residue was dissolved in EtOAc:MeOH and stirred with Acros Organics silica gel 60 (0.2–0.5 mm) at room temperature overnight.

##### Synthesis of (1*S*,4*R*)-4-((1*H*-Indol-3-yl)methyl)-1-methyl-1,2-dihydro-6*H*-pyrazino[2,1-*b*]quinazoline-3,6(4*H*)-dione (**2**, **Fumiquinazoline F**)

Tripeptide **11b** (1 equiv., 420 mg, 0.647 mmol) was dehydrated for 5 h with Ph_3_P (5 equiv., 849 mg, 3.24 mmol), I_2_ (4.9 equiv., 805 mg, 3.17 mmol), and *N*,*N*-diisopropylethylamine (10.1 equiv., 1.14 mL, 6.54 mmol) in dry CH_2_Cl_2_ (40 mL). Deprotection with piperidine (25.84 equiv., 1.7 mL, 17.2 mmol) in EtOAc (6.7 mL) for 1 h, was followed by stirring with silica gel (3.82 g) overnight in EtOAc:MeOH (2:1, 15 mL). After filtration, the compound was purified by preparative TLC using EtOAc as the mobile phase. The compound was further purified by crystallization in MeOH and fumiquinazoline F (**2**) was obtained as a white solid in a 35% yield.

(1*S*,4*R*)-4-((1*H*-indol-3-yl)methyl)-1-methyl-1,2-dihydro-6*H*-pyrazino[2,1-*b*]quinazoline-3,6(4*H*)-dione (**2**, **Fumiquinazoline F**): White solid (81 mg, 35% yield). m.p. 138.5–140.5 °C (lit. mp 137 °C (foam) [27]), [α_D_^25^] = −334 (*c* 0.030, THF) (lit. [α_D_^30^] −516 (c 0.74, CHCl_3_) [27]). ^1^H NMR (300 MHz, CDCl_3_) δ 8.36 (d, *J* = 8.0 Hz, 1H, H-8), 8.25 (br s, 1H, NH Indole), 7.80–7.73 (m, 1H, H-10), 7.59 (d, *J* = 8.0 Hz, 1H, H-9), 7.53 (t, *J* = 7.5 Hz, 1H, H-11), 7.39 (d, *J* = 8.0 Hz, 1H, H-23), 7.29 (d, *J* = 8.2 Hz, 1H, H-20), 7.12 (t, *J* = 7.5 Hz, 1H, H-21), 6.91 (t, *J* = 7.5 Hz, 1H, H.22), 6.70 (d, *J* = 2.3 Hz, 1H, H-17), 6.46 (br s, 1H, NH amide), 5.68 (t, *J* = 4.4 Hz, 1H, H-4), 3.67 (t, *J* = 4.9 Hz, 2H, H-15, H-15′), 3.11 (q, *J* = 6.6 Hz, 1H, H-1), 1.35 (d, *J* = 6.6 Hz, 3H, CH_3_). ^13^C NMR (75 MHz, CDCl_3_) δ 169.24 (C-3), 160.79 (C-6), 151.61 (C-14), 147.06 (C-12), 135.92 (C-19), 134.69 (C-10), 127.29 (C-24), 127.14 (C-9), 127.11 (C-11), 126.85 (C-8), 123.54 (C-17), 122.54 (C-21), 120.22 (C-7), 120.02 (C-22), 118.47 (C-23), 111.18 (C-20), 109.38 (C-16), 57.54 (C-4), 49.15 (C-1), 27.04 (C-15), 19.13 (CH_3_). (According to the literature [27]). HPLC-DAD: Peak purity = 95.3%; Chromatographic Purity = 100%.

##### Synthesis of (1*S*,4*R*)-4-((1*H*-Indol-3-yl)methyl)-8-fluoro-1-isobutyl-1,2-dihydro-6*H*-pyrazino[2,1-*b*]quinazoline-3,6(4*H*)-dione (**12a**)

Tripeptide **11c** (1 equiv., 300 mg, 0.434 mmol) was dehydrated with Ph_3_P (5 equiv., 570 mg, 2.17 mmol) and I_2_ (4.9 equiv., 540 mg, 2.13 mmol) in the presence of *N,N*-diisopropylethylamine (10.10 equiv., 764 µL, 4.39 mmol) in dry CH_2_Cl_2_ (26.1 mL). Deprotection with piperidine (25.84 equiv., 1.1 mL, 11.18 mmol) in EtOAc (4.42 mL) for 1 h, was followed by stirring with silica gel (2.5 g) overnight in EtOAc:MeOH (2:1, 10 mL). Purification by preparative TLC with silica plates and EtOAc:CH_2_Cl_2_ (6:4) as the mobile phase gave **12a** as a very-light-pink solid in a 22% yield.

(1*S*,4*R*)-4-((1*H*-indol-3-yl)methyl)-8-fluoro-1-isobutyl-1,2-dihydro-6*H*-pyrazino[2,1-*b*]quinazoline-3,6(4*H*)-dione (**12a**): Light-pink solid (40 mg, 22% yield). m.p. 107.3–111.7 °C. [α_D_^25^] = −464 (*c* 0.037, THF). ^1^H NMR (300 MHz, CDCl_3_) δ 8.11 (s, 1H, NH indole), 7.99 (dd, *J* = 8.3, 2.9 Hz, 1H, H-8), 7.60 (dd, *J* = 9.0, 4.8 Hz, 1H, H-11), 7.52–7.44 (m, 2H, H-10, H-23), 7.29 (dt, *J* = 8.2, 0.9 Hz, 1H, H-20), 7.13 (ddd, *J* = 8.2, 7.0, 1.2 Hz, 1H, H-21), 6.98 (ddd, *J* = 8.0, 7.0, 1.0 Hz, 1H, H-22), 6.66 (d, *J* = 2.5 Hz, 1H, H-17), 5.75 (s, 1H, NH amide), 5.65 (dd, *J* = 5.3, 2.9 Hz, 1H, H-4), 3.77 (dd, *J* = 14.9, 2.9 Hz, 1H, H-15), 3.65 (dd, *J* = 15.0, 5.3 Hz, 1H, H-15′), 2.73 (dd, *J* = 9.6, 3.4 Hz, 1H, H-1), 2.04–1.92 (m, 1H, H-26), 1.41–1.29 (m, 2H, H-25, H-25′), 0.77 (d, *J* = 6.3 Hz, 3H, CH_3_), 0.29 (d, *J* = 6.4 Hz, 3H, CH_3_). ^13^C NMR (75 MHz, CDCl_3_) δ 169.20 (C-3), 160.97 (C-9, d, *J =* 248.7 Hz), 160.17 (C-6, d, *J =* 3.6 Hz), 150.85 (C-14), 143.67 (C-12), 136.11 (C-19), 129.81 (C-11, d, *J* = 8.2 Hz), 127.11 (C-24), 123.48 (C-17), 123.32 (C-10, d, *J* = 23.8 Hz), 122.85 (C-21), 121.40 (C-7, d, *J =* 8.8 Hz) 120.35 (C-22), 118.72 (C-23), 111.66 (C-8, d, J = 23.8 Hz), 111.15 (C-20), 109.65 (C-16), 57.49 (C-4), 50.69 (C-1), 40.15 (C-25), 27.06 (C-15), 24.14 (C-26), 23.30 (CH3), 19.71 (CH3). HRMS (ESI^−^): *m*/*z* [C_24_H_22_FN_4_O_2_-H]^−^ calcd. for [C_24_H_22_FN_4_O_2_]: 417.17213; found 417.17342. Peak purity = 99.9%; Chromatographic Purity = 97.9%

##### Synthesis of Alkaloid (1*S*,4*R*)-4-((1*H*-Indol-3-yl)methyl)-9-fluoro-1-isobutyl-1,2-dihydro-6*H*-pyrazino[2,1-*b*]quinazoline-3,6(4*H*)-dione (**12b**)

Tripeptide **11d** (1 equiv., 170 mg, 0.246 mmol) was dehydrated with Ph_3_P (5 equiv., 323 mg, 1.23 mmol) and I_2_ (4.9 equiv., 306 mg, 1.21 mmol) in the presence of *N,N*-diisopropylethylamine (10.10 equiv., 433 µL, 2.49 mmol) in dry CH_2_Cl_2_ (14.7 mL), followed by deprotection with piperidine (25.84 equiv., 0.607 mL, 6.15 mmol) in EtOAc (2.43 mL) for 1 h, and by stirring with silica gel (1.38 g) overnight in EtOAc:MeOH (2:1, 5.5 mL). Purification by preparative TLC with silica plates and EtOAc:CH_2_Cl_2_ (6:4) as the mobile phase gave **12b** as a light-yellow solid in a 20% yield.

(1*S*,4*R*)-4-((1*H*-indol-3-yl)methyl)-9-fluoro-1-isobutyl-1,2-dihydro-6*H*-pyrazino[2,1-*b*]quinazoline-3,6(4*H*)-dione (**12b**): Light-yellow solid (20 mg, 20% yield). m.p. 112.0–114.7 °C. [α_D_^25^] = −273 (*c* 0.037, THF). ^1^H-NMR (300 MHz, CDCl_3_) δ 8.37 (dd, *J* = 9.5, 6.1 Hz, 1H, H-8), 8.16 (s, 1H, NH indole), 7.46 (d, *J* = 8.0, 1H, H-23), 7.30 (dt, *J* = 8.2, 0.9 Hz, 1H, H-20), 7.30–7.21 (m, 1H, H-9), 7.22 (d, *J* = 2.4 Hz, 1H, H-11), 7.14 (ddd, *J* = 8.3, 7.0, 1.2 Hz, 1H, H-21), 6.98 (ddd, *J* = 8.1, 7.0, 1.0 Hz, 1H, H-22), 6.66 (d, *J* = 2.4 Hz, 1H, H-17), 5.80 (s, 1H, NH amide), 5.65 (dd, *J* = 5.3, 2.9 Hz, 1H, H-4), 3.76 (dd, *J* = 15.1, 2.9 Hz, 1H, H-15), 3.63 (dd, *J* = 15.0, 5.3 Hz, 1H, H-15′), 2.71 (dd, *J* = 9.5, 3.4 Hz, 1H, H-1), 1.97 (ddd, *J* = 15.1, 11.2, 2.9 Hz, 1H, H-25), 1.40–1.29 (m, 2H, H-25′, H-26), 0.76 (d, *J* = 6.3 Hz, 3H, CH_3_), 0.29 (d, *J* = 6.4 Hz, 3H, CH_3_). ^13^C NMR (75 MHz, CDCl_3_) δ 169.22 (C-3), 166.65 (C-10, d, *J* = 254.9 Hz), 160.10 (C-6), 152.95 (C-14), 149.14 (C-12, d, *J* = 13.1 Hz), 136.12 (C-19), 129.64 (C-8, d, *J* = 10.8 Hz), 127.10 (C-24), 123.52 (C-17), 122.84 (C-21), 120.33 (C-22), 118.68 (C-23), 116.94 (C-7, d, *J* = 1.9 Hz), 115.97 (C-9, d, *J* = 23.7 Hz), 112.62 (C-11, d, *J* = 21.8 Hz), 111.18 (C-20), 109.58 (C-16), 57.30 (C-4), 50.86 (C-1), 40.17 (C-25), 27.13 (C-15), 24.14 (C-26), 23.26 (CH_3_), 19.74 (CH_3_). HRMS (ESI^−^): *m*/*z* [C_24_H_22_FN_4_O_2_-H]^−^ calcd. for [C_24_H_22_FN_4_O_2_]: 417.17213; found 417.17347. HPLC-DAD: Peak purity = 99.9%; Chromatographic Purity = 94.27%

##### Synthesis of Alkaloid ((1*S*,4*S*)-8,10-Dichloro-4-((6-fluoro-1*H*-indol-3-yl)methyl)-1-isobutyl-1,2-dihydro-6*H*-pyrazino[2,1-*b*]quinazoline-3,6(4*H*)-dione (**12c**)

Tripeptide **11e** (1 equiv., 89.5 mg, 0.118 mmol) was dehydrated with Ph_3_P (5 equiv., 154.5 mg, 0.589 mmol) and I_2_ (4.9 equiv., 147, 0.577 mmol) in the presence of *N,N*-diisopropylethylamine (10.10 equiv., 208 µL, 1.19 mmol) in dry CH_2_Cl_2_ (7.16 mL), followed by deprotection with piperidine (25.84 equiv., 0.291 mL, 2.95 mmol) in EtOAc (1.17 mL) for 1 h, and by stirring with silica gel (0.661 g) overnight in EtOAc:MeOH (2:1, 2.6 mL). Purification by preparative TLC with silica plates using EtOAc:CH_2_Cl_2_ (6:4) as the mobile phase gave **12c** as a light-yellow solid in a 8.2% yield.

((1*S*,4*S*)-8,10-dichloro-4-((6-fluoro-1*H*-indol-3-yl)methyl)-1-isobutyl-1,2-dihydro-6*H*-pyrazino[2,1-*b*]quinazoline-3,6(4*H*)-dione (**12c**): Light-yellow solid (4.7 mg, 8.2% yield). m.p. 163.0–168 °C. [α_D_^25^] = +450 (*c* 0.04, THF). ^1^H-NMR (300 MHz, CDCl_3_) δ 8.24 (d, *J* = 2.4 Hz, 1H, H-8), 8.19 (s, 1H, NH indole), 7.83 (d, *J* = 2.4 Hz, 1H, H-10), 7.33 (dd, *J* = 8.8, 5.2 Hz, 1H, H-23), 6.97 (dd, *J* = 9.5, 2.3 Hz, 1H, H-20), 6.74 (td, *J* = 9.2, 2.3 Hz, 1H, H-22), 6.65 (d, *J* = 2.3 Hz, 1H, H-17), 6.58 (s, 1H, NH amide), 5.47 (t, *J* = 4.3 Hz, 1H, H-4), 4.39 (dt, *J* = 10.2, 4.2 Hz, 1H, H-1), 3.75–3.70 (m, 2H, H-15, H-15′), 1.23–1.16 (m, 1H, H-26), 1.05–0.98 (m, 1H, H-25, H-25′), 0.74 (d, *J* = 6.5 Hz, 3H, CH_3_), 0.60 (d, *J* = 6.4 Hz, 3H, CH_3′_). ^13^C NMR (75 MHz, CDCl_3_) δ 166.95 (C-3), 159.91, (d, *J* = 164.3 Hz, C-21), 159.57 (C-6), 151.99 (C-14), 142.68 (C-12), 135.73 (d, *J* = 12.8 Hz, C-19), 135.11 (C-10), 132.20 (C-9), 132.16 (C-11), 124.99 (C-8), 124.38 (C-24), 123.86 (d, *J* = 3.7 Hz, C-17), 121.98 (C-7), 119.80 (d, *J* = 10.2 Hz, C-23), 109.32 (d, *J* = 18.0 Hz, C-22), 108.88 (C-16), 97.51 (d, *J* = 26.1 Hz, C-20), 57.00 (C-4), 54.25 (C-1), 45.88 (C-25), 24.10 (C-26), 22.61 (CH_3_), 20.75 (CH_3′_). HRMS (ESI^−^): *m*/*z* [C_24_H_20_Cl_2_FN_4_O_2_-H]^−^ calcd. for [C_24_H_20_Cl_2_FN_4_O_2_]: 485.09419; found 485. 09547. Peak purity = 94.2%; Chromatographic Purity = 100%

##### Synthesis of Alkaloid (1*S*,4*S*)-8,10-Dichloro-4-((7-chloro-1*H*-indol-3-yl)methyl)-1-isobutyl-1,2-dihydro-6*H*-pyrazino[2,1-*b*]quinazoline-3,6(4*H*)-dione (**12d**)

Tripeptide **11f** (1 equiv., 204 mg, 0.262 mmol) was dehydrated with Ph_3_P (5 equiv., 345 mg, 1.31 mmol) and I_2_ (4.9 equiv., 327 mg, 1.29 mmol) in the presence of *N,N*-diisopropylethylamine (10.10 equiv., 462 µL, 2.65 mmol) in dry CH_2_Cl_2_ (15.9 mL), followed by deprotection with piperidine (25.84 equiv., 0.646 mL, 6.55 mmol) in EtOAc (2.59 mL) for 1 h, and stirring with silica gel (1.49 g) overnight in EtOAc:MeOH (2:1, 6 mL). Purification by preparative TLC with silica plates and EtOAc:CH_2_Cl_2_ (6:4) as the mobile phase gave **12d** as a light-yellow solid in a 4.2% yield.

(1*S*,4*S*)-8,10-dichloro-4-((7-chloro-1*H*-indol-3-yl)methyl)-1-isobutyl-1,2-dihydro-6*H*-pyrazino[2,1-*b*]quinazoline-3,6(4*H*)-dione (**12d**): Light-yellow solid (5.5 mg, 4.2% yield) m.p. 196.0–199.3 °C. [α_D_^25^] = +510 (*c* 0.033, THF). ^1^H-NMR (500 MHz, DMSO-d_6_) δ 10.21 (s, 1H, NH indole), 8.13 (d, *J* = 2.4 Hz, 1H, H-8), 7.85 (d, *J* = 2.7 Hz, 1H, NH amide), 7.75 (d, *J* = 2.4 Hz, 1H, H-10), 7.21 (d, *J* = 8.0 Hz, 1H, H-23), 7.00 (d, *J* = 7.6 Hz, 1H, H-21), 6.75 (t, *J* = 7.8 Hz, 1H, H-22), 6.69 (d, *J* = 2.5 Hz, 1H, H-17), 5.31 (dd, *J* = 5.2, 3.6 Hz, 1H, H-4), 4.24 (ddd, *J* = 9.2, 5.3, 3.4 Hz, 1H, H-1), 3.64 (dd, *J* = 15.1, 5.3 Hz, 1H, H-15), 3.60 (dd, *J* = 15.1, 3.7 Hz, 1H, H-15′), 1.24–1.18 (m, 1H, H-26), 0.73–0.66 (m, 2H, H-25, H-25′), 0.62 (d, *J* = 6.6 Hz, 3H, CH_3_), 0.50 (d, *J* = 6.6 Hz, 3H, CH_3_). ^13^C NMR (126 MHz, DMSO-d_6_) δ 166.32 (C-3), 159.49 (C-6), 152.66 (C-14), 142.66 (C-12), 134.68 (C-10), 133.06 (C-19), 132.49 (C-11), 131.59 (C-9), 129.45 (C-24), 125.03 (C-17), 124.71 (C-8), 121.80 (C-7), 121.14 (C-21), 119.99 (C-22), 117.22 (C-23), 116.64 (C-20), 109.51 (C-16), 57.06 (C-4), 53.91 (C-1), 46.34 (C-25), 26.41 (C-15), 23.71 (C-26), 22.23 (CH_3_), 20.98 (CH_3′_). HRMS (ESI^−^): *m*/*z* [C_24_H_20_Cl_3_N_4_O_2_-H]^−^ calcd. for [C_24_H_20_Cl_3_N_4_O_2_]: 501.06464; found 501.06607. Peak purity = 97.4%; Chromatographic Purity = 100%

### 3.2. Microbiology

#### 3.2.1. Reagents and Media

The reagents and media used in the microbiological assays were as follows: ethidium bromide (EB), CCCP, crystal violet (CV), DMSO, Luria-Bertani (LB) broth, and LB agar, tryptic soy broth (TSB), and tryptic soy agar (TSA) purchased from Sigma-Aldrich Chemie GmbH (Steinheim, Germany); phosphate-buffered saline (PBS; pH 7.4), reserpine (RES), and ethanol purchased from Sigma-Aldrich Chemie GmbH (Steinheim, Germany); Mueller–Hinton (MH) agar purchased from Sigma-Aldrich Chemie GmbH (Steinheim, Germany) and from BioKar Diagnostics (Allone, France); dimethylsulfoxide (DMSO) purchased from Sigma-Aldrich Chemie GmbH (Steinheim, Germany) and Alfa Aesar, (Kandel, Germany); Dulbecco’s modified Eagle’s medium (DMEM; Sigma-Aldrich, St. Louis, MO, USA), containing 4.5 g/L glucose, supplemented with 10% heat-inactivated fetal bovine serum (FBS); and doxorubicin (Teva Pharmaceuticals, Debrecen, Hungary).

#### 3.2.2. Bacterial Strains

The microbiological assays were performed using two Gram-positive reference bacterial species from the American Type Culture Collection (ATCC): *Staphylococcus aureus* ATCC 29213, and *Enterococcus faecalis* ATCC 29212; and Gram-negative: *Escherichia coli* ATCC 25922 and *Pseudomonas aeruginosa* ATCC 27853, as well as clinically relevant strains, which included methicillin-resistant *S. aureus* (MRSA; isolate #66/1, obtained from public buses [45]), methicillin- and ofloxacin-resistant *S. aureus* (clinical isolate #272123), vancomycin-resistant *Enterococcus* (VRE; *E. faecalis* #B3/101 isolated from river water [46]), a cefotaxime resistant strain of *E. coli* (#SA/2 isolated from a patient in a hospital) and a wild-type *E. coli* K-12 AG100 strain [argE3 thi-1 rpsL xyl mtl Δ(gal-uvrB) supE44], expressing the AcrAB–TolC efflux pump (EP) at its basal level and the AcrAB–TolC-deleted mutant *E. coli* AG100 A. For the compounds that showed relevant activity against *E. faecalis* B3/101, two VRE strains (#PSi/107 and #PFi/2) isolated from raw sewage were also tested [47].

Frozen stocks of all strains were grown on Mueller–Hinton agar at 37 °C for 24 h. All bacterial strains were sub-cultured on MH agar and incubated overnight at 37 °C before each assay to obtain fresh cultures.

Stock solutions of each compound (10 mg/mL or 10 mM) were prepared in DMSO. In all of the experiments, the final in-test concentration of DMSO was kept below 1%, as recommended by the CLSI [48].

#### 3.2.3. MIC Determination

The MIC of each compound was determined by the broth microdilution method, in accordance with the recommendations of the CLSI [48]. Two-fold serial dilutions of the compounds were prepared in Mueller–Hinton Broth 2 (MHB2–Sigma-Aldrich, St. Louis, MO, USA). Sterility and growth controls were included in each assay. Quality controls were performed with erythromycin for the Gram-positive bacterial strains and with colistin for the Gram-negative bacterial strains. Purity check and colony counts of the inoculum suspensions were also performed to ensure that the final inoculum density closely approximated 5 × 10^5^ CFU/mL. The MIC was determined as the lowest concentration at which no visible growth was observed. The MBC was assessed by spreading 10 µL of culture collected from wells showing no visible growth on MH agar plates. The MBC was determined as the lowest concentration at which no colonies grew after 18–24 h incubation at 37 °C. The reported MIC results arose from at least three independent experiments.

#### 3.2.4. Real-Time Ethidium Bromide Accumulation Assay

The efflux-pump-inhibiting activity of the compounds was tested on *S. aureus* ATCC 25923, *S. aureus* MRSA 272123, *E. coli* AG100, and *E. coli* AG100A strains by real-time fluorimetry monitoring the intracellular accumulation of the efflux pump substrate EB using a CLARIOstar Plus plate reader (BMG Labtech, Ortenberg, Germany). RES was applied at 25 µM and CCCP was applied at 50 µM as the positive controls; the solvent DMSO was applied at 1 *v*/*v*%. The examined bacterial strains were cultured at 37 °C in a shaking incubator until they reached an optical density (OD) of 0.6 at 600 nm. The bacterial cells were then washed with 500 µL PBS and centrifuged at 13,000× *g* for 2 min; then, the pellet was re-suspended in 1 mL PBS. The compounds were applied at an MIC/2 concentration and where the MIC was >100 µM, the tested concentration was 50 µM or 100 µM in PBS supplemented with a non-toxic concentration of EB (2 µg/mL). Afterwards, the solutions were pipetted into a 96-well black microtiter plate (Greiner Bio-One Hungary Kft, Hungary) in 50 µL, and 50 µL of the bacterial suspension (OD_600_ 0.6) was pipetted to the wells. Then, the plates were measured with the CLARIOstar plate reader, and the fluorescence was recorded at excitation and emission wavelengths of 530 nm and 600 nm, respectively, every minute for 1 h. From the real-time data, the relative fluorescence index (RFI) of the last time point (minute 60) was calculated according to the subsequent equation:

RFI = (RF_treated_ − RF_untreated_)/RF_untreated_

where RF_treated_ is the relative fluorescence (RF) at the last time point of EB retention curve in the presence of an inhibitor and RF_untreated_ is the RF at the last time point of the EB retention curve of the untreated control having the solvent control (DMSO).

#### 3.2.5. Measuring Biofilm Formation Using Crystal Violet

The biofilm-forming ability of the *S. aureus* strains was studied in 96-well microtiter plates using TSB broth in the presence of compounds. The biofilm-forming ability of the *E. coli* strains was studied in 96-well microtiter plates using LB broth in the presence of the compounds. The overnight cultures of the bacteria were diluted to an OD of 0.1 at 600 nm and then added to each well, with the exception of the medium control wells, and compounds were added at an MIC/2 concentration (where the MIC was >100 µM, the tested concentration was 50 µM or 100 µM). The final volume was 200 μL in each well. The positive control was CCCP at 50 µM. Plates were incubated for 48 h at 30 °C with gentle shaking at 100 rpm. After the incubation period, the medium was removed and the plate was washed with water to remove unattached cells. A total of 200 μL CV (0.1% [*v*/*v*]) was added to each wells and incubated for 15 min at room temperature. CV was discarded from the wells and the plate was washed with water again. Then, 200 μL of 70% ethanol was added to each well and the biofilm formation was determined by measuring the OD at 600 nm using a Multiscan EX ELISA plate reader (Thermo Labsystems, Cheshire, WA, USA). The antibiofilm effect of the compounds was expressed as the percentage (%) decrease in biofilm formation.

#### 3.2.6. Antibiotic Potentiation Assay

To evaluate the combined effect of the compounds and clinically relevant antimicrobial drugs, initial screening was conducted using the Kirby–Bauer disk diffusion method, as recommended by the CLSI [39]. A set of antibiotic disks (Oxoid, Basingstoke, England), to which the isolates were resistant, was elected: cefotaxime (CTX, 30 mg) for extended-spectrum beta-lactamase-producer *E. coli* SA/2, oxacillin (OX, 1 mg) for *S. aureus* 66/1, and vancomycin (VA, 30 mg) for *E. faecalis* B3/101. Antibiotic disks alone (controls) and antibiotic disks impregnated with 15 mg of each compound were also placed on the MH agar plates. Sterile 6 mm blank papers impregnated with 15 mg of each compound alone were also tested. A blank disk with DMSO was used as a negative control. MH inoculated plates were incubated for 18–20 h at 37 °C. Potential synergism was recorded when the halo of an antibiotic disk, impregnated with a compound, was greater than the halo of the antibiotic- or compound-impregnated blank disk alone. The most relevant compounds were also further tested for synergism using the broth microdilution method, using half of the MIC concentration of each compound and varied concentrations of each antibiotic, following the same procedure previously described for the MIC determination. Potential synergism was recorded when the MIC of the antibiotic was reduced to at least half the original value.

#### 3.2.7. Cytotoxicity Assay

The effects of increasing concentrations of compounds on cell growth were tested in 96-well flat-bottomed microtiter plates. The compounds were diluted in a volume of 100 μL of medium. Afterwards, 1 × 10^4^ cells in 100 μL of medium were added to each well, with the exception of the medium control. In the case of NIH/3T3 cells, the compounds were added after seeding the cells at 37 °C overnight. The plates were incubated at 37 °C for 24 h; after the incubation period, 20 μL of MTT solution (from a 5 mg/mL stock) was added to each well. After 4 h of incubation at 37 °C, 100 μL of SDS solution (10% in 0.01 M HCl) was added to each well and the plates were incubated at 37 °C overnight. Cell growth was determined by measuring the optical density (OD) at 540 nm (ref. 630 nm) with a Multiscan EX ELISA reader. Inhibition of cell growth was determined as IC_50_ values, defined as the inhibitory dose that reduced the growth of the cells exposed to the tested compounds by 50%. IC_50_ values and the SD of triplicate experiments were calculated by using GraphPad Prism software version 5.00 for Windows with a nonlinear regression curve fit (GraphPad Software, San Diego, CA, USA; www.graphpad.com, accessed on 14 July 2022). Doxorubicin (from a 2 mg/mL stock solution, Teva Pharmaceuticals) was used as the positive control. The solvent (DMSO) did not affect the cell growth in the tested concentration (2%).

#### 3.2.8. Docking Studies

The crystal structure of the AcrB (PDB: 2DRD) [49], AcrA (PDB: 2F1M) [50], and TolC (PDB: 1EK9) [51] portions of the AcrAB–TolC bacterial efflux system, downloaded from the Protein Data Bank (PDB) [52], were used for this study. The tested compounds were drawn with ChemDraw (PerkinElmer Informatics) and minimized using ArgusLab. Docking was carried out using AutoDock Vina (Scripps, CA, USA) [53] in the sites described in [40,54]. The NorA efflux pump did not have an available crystal structure, and a homology model was prepared. The model was generated using the Swiss Model server [55] and the sequence deposited in Uniprot (Q5HHX4) [56], using the EmrD pump from *E. coli* (PDB: 2GFP) as the homolog, as described in [43]. The sequence similarity was 0.28, the coverage was 0.91, and the sequence identity was 17.33%. The site analyzed was that described in [57]. The top nine poses were collected for each molecule and the lowest docking score value was associated with the most favorable binding conformation.

## 4. Conclusions

The development of new antimicrobial agents is extremely important in the current society, given the rising levels of drug resistance. In the current study, a multi-step synthetic pathway was followed to achieve the synthesis of natural products glyantrypine (**1**) and fumiquinazoline F (**2**) as well as of four new halogenated derivatives (**12a**–**12d**). To the best of our knowledge, derivatives **12a**–**12d** were synthesized in this study for the first time.

Among the results of the antibacterial screening, it is worth highlighting the relevant antibacterial activities of the synthesized compounds **12a**–**12d** towards Gram-positive resistant bacterial strains, with compound **12d** being active against three strains of *S. aureus*, including MRSA (MIC = 16–32 μM) and two strains of *E. faecalis* including VRE (MIC = 64 μM). Compound **12d** showed comparable activity to neofiscalin A (**3**) and the previously reported *hit* compound **4** against MRSA (*S. aureus* 66/1) [26].

Concerning EPI activity, several compounds showed higher RFI values than the positive controls in the ethidium bromide accumulation assay, suggesting that they might show potential as efflux pump inhibitors. In this assay, compound **12c** seemed to be the most potent, showing promising RFI values in all three bacterial strains tested. Moreover, compounds **4** and **13a**–**13c**, for example, were very effective at reducing the formation of MRSA biofilms (81–91% inhibition rates).

In conclusion, this work allowed the synthesis of a small library of new indole-containing pyrazino[2,1-*b*]quinazoline-3,6-diones and reinforces the idea that halogenated fumiquinazoline-related derivatives constitute a novel and relevant approach in the development of new antibacterial agents as well as in the development of compounds capable of fighting common resistance mechanisms. This study also allowed a more thorough study of the structure–activity relationship of the fumiquinazoline derivatives, that can provide useful insight for future optimization of the antibacterial potential of these compounds.

Future work will involve further studies to evaluate the biological activities of the synthesized compounds. Studies to investigate the mechanism of action of the compounds would also be interesting. Furthermore, molecular modifications to reduce the cytotoxicity of some of the most potent compounds might be required in the future.

## Figures and Tables

**Table 1:** Purity percentages, optical rotation, and enantiomeric excess of the synthesized derivatives. THF = Tetrahydrofuran.

Compound	Peak Purity	Chromatographic Purity	[α_D_^25^]	Enantiomeric Excess (*ee*)
**1**	97.8%	100%	−390 (*c* 0.033, THF)	>99%
**2**	95.3%	100%	−334 (*c* 0.030, THF)	>99%
**12a**	99.9%	97.9%	−464 (*c* 0.037, THF)	>99%
**12b**	99.9%	94.3%	−273 (*c* 0.037, THF)	>99%
**12c**	94.2%	100%	+450 (*c* 0.04, THF)	>99%
**12d**	97.4%	100%	+510 (*c* 0.033, THF)	>99%

**Table 2:** MIC values from each compound µM (µg/mL) against a panel of Gram-positive bacteria.

	*S. aureus* Strains			*E. faecalis* Strains			
Compound	A	B	C	D	E	F	G
**1**	>100	>100	>100	>100	>100	n.t	n.t
**2**	>100	>100	>100	>100	>100	n.t	n.t
**12a**	>100	77 (32)	>100	>100	77 (32)	>100	>100
**12b**	77 (32)	77 (32)	100 (41.8)	>100	38 (16)	>100	>100
**12c**	33 (16)	33(16)	12.50 (6.1)	33–66 (16–32)	33 (16)	>100	>100
**12d**	32 (16)	16 (8)	25 (12.6)	64 (32)	64 (32)	>100	>100
**4**	n.t	n.t	>100	n.t	n.t	n.t	n.t
**13a**	n.t	n.t	>100	n.t	n.t	n.t	n.t
**13b**	n.t	n.t	>100	n.t	n.t	n.t	n.t
**13c**	n.t	n.t	100 (40.05)	n.t	n.t	n.t	n.t
**13d**	n.t	n.t	>100	n.t	n.t	n.t	n.t

A = *S. aureus* ATCC 29213; B = *S. aureus* 66/1 (MRSA) C = *S. aureus* MRSA 272123; D = *E. faecalis* ATCC 29212; E = *E. faecalis* B3/101 (VRE); F = *E. faecalis* PSi/107; G = *E. faecalis* PFi/2; n.t = not tested in this work.

**Table 3:** Relative fluorescence index (RFI) of the tested derivatives **1**, **2**, **12a**–**12d**, **4**, and **13a**–**13d**.

	*E. coli* AG100		*E. coli* AG100A		*S. aureus* 272123	
Compound	Concentration (μM)	RFI ± SD	Concentration (μM)	RFI ± SD	Concentration (μM)	RFI ± SD
**1**	100 50	−0.04 ± 0.10 −0.24 ± 0.05	100	0.12 ± 0.01	100	−0.01 ± 0.04
					50	0.23 ± 0.02
**2**	100 50	0.17 ± 0.10 −0.31 ± 0.02	100	0.26 ± 0.10	100	0.05 ±0.02
					50	−0.01 ± 0.02
**12a**	100 50	2.45 ± 0.26 −0.17 ± 0.02	100	0.59 ± 0.03	100	1.03 ± 0.02
					50	0.63 ± 0.04
**12b**	100 50	1.47 ± 0.12 −0.07 ± 0.06	100	0.77 ± 0.17	50	0.96 ± 0.07
**12c**	100	3.60 ± 0.01	100	4.40 ± 1.78	6.25	0.49 ± 0.01
	50	0.24 ± 0.16				
**12d**	100	0.32 ± 0.10	100	0.23 ± 0.05	12.5	0.34 ± 0.01
**4**	50	0.01 ± 0.02	50	0.26 ± 0.07	50	1.13 ± 0.20
**13a**	50	0.38 ± 0.01	50	0.48 ± 0.58	50	2.39 ± 0.09
**13b**	50	0.52 ± 0.18	50	0.12 ± 0.05	50	1.53 ± 0.02
**13c**	50	0.17 ± 0.02	50	0.13 ± 0.05	50	0.94 ± 0.04
**13d**	50	−0.01 ± 0.03	50	0.55 ± 0.29	50	0.19 ± 0.08
**CCCP**	50	1.56 ± 0.01	50	1.13 ± 0.02	-	-
**RES**	-	-	-	-	25	0.68 ± 0.05

RFI: relative fluorescence index; SD: standard deviation.

**Table 4:** Cytotoxic activity of the tested compounds on normal NIH/3T3 cell line. Doxorubicin (DOXO) was used as a positive control. Dimethyl sulfoxide (DMSO) was applied as the solvent control.

Cytotoxicity on NIH/3T3	
Compounds	IC_50_ ± SD (µM)
**1**	>100
**2**	>100
**12a**	52.20 ± 0.75
**12b**	58.04 ± 1.58
**12c**	46.15 ± 0.66
**12d**	47.23 ± 1.55
**DOXO**	3.72 ± 0.15
**DMSO**	>2%

## Data Availability

The data presented in this study are available in the Supplementary Materials.

## Supplementary Materials

The following supporting information can be downloaded at: https://www.mdpi.com/article/10.3390/antibiotics12050922/s1: NMR data (Figures S1–S47), HRMS data (Figures S47–S51), HPLC-DAD Purity Data (Table S1), Enantiomeric Excess data (Table S2), and Docking Data (Table S3 and Figure S52).
