# Supplementary material for: Effect of Indole-Containing Pyrazino[2,1-b]quinazoline-3,6-diones in the Virulence of Resistant Bacteria
Source: Antibiotics (Basel). 2023 May 17;12(5):922. doi: 10.3390/antibiotics12050922 (PMC10215404; doi:10.3390/antibiotics12050922)
Supplement: Supplementary file 1 [file antibiotics-12-00922-s001.zip › antibiotics-2396549-supplementary.pdf]

Supporting Information

Effect of Indole-Containing Pyrazino[2,1-b]quinazoline-3,6-diones in the Virulence of Resistant Bacteria

Mariana C. Almeida[1,2], Nikoletta Szemerédi[3], Fernando Durães[1,2], Solida Long[1,4], Diana I. S. P. Resende[1,2*], Paulo Martins da Costa[2,5], Madalena Pinto[1,2], Gabriella Spengler[3], Emília Sousa[1,2*]

[1] Laboratório de Química Orgânica e Farmacêutica, Faculdade de Farmácia, Universidade do Porto, Rua de Jorge Viterbo Ferreira 228, 4050-313 Porto, Portugal;
[2] CIIMAR - Centro Interdisciplinar de Investigação Marinha e Ambiental, Terminal de Cruzeiros do Porto de Leixões, 4450-208 Matosinhos, Portugal
[3] Department of Medical Microbiology, Albert Szent-Gyorgyi Health Center and Albert Szent-Gyorgyi Medical School, University of Szeged, Hungary.
[4] Department of Bioengineering, Royal University of Phnom Penh, Russian Confederation Blvd, 12156 Phnom Penh, Cambodia
[5] ICBAS – Instituto de Ciências Biomédicas Abel Salazar, Universidade do Porto, Rua de Jorge Viterbo Ferreira 228, 4050-313 Porto, Portugal

***Correspondence: D. I. S. P. R. – dresende@ff.up.pt, E. S. – esousa@ff.up.pt**

Table of Contents

1. NMR Data 2
2. HRMS Data 26
3. HPLC-DAD Purity Data 28
4. Enantiomeric Excess Data 31

1. NMR Data

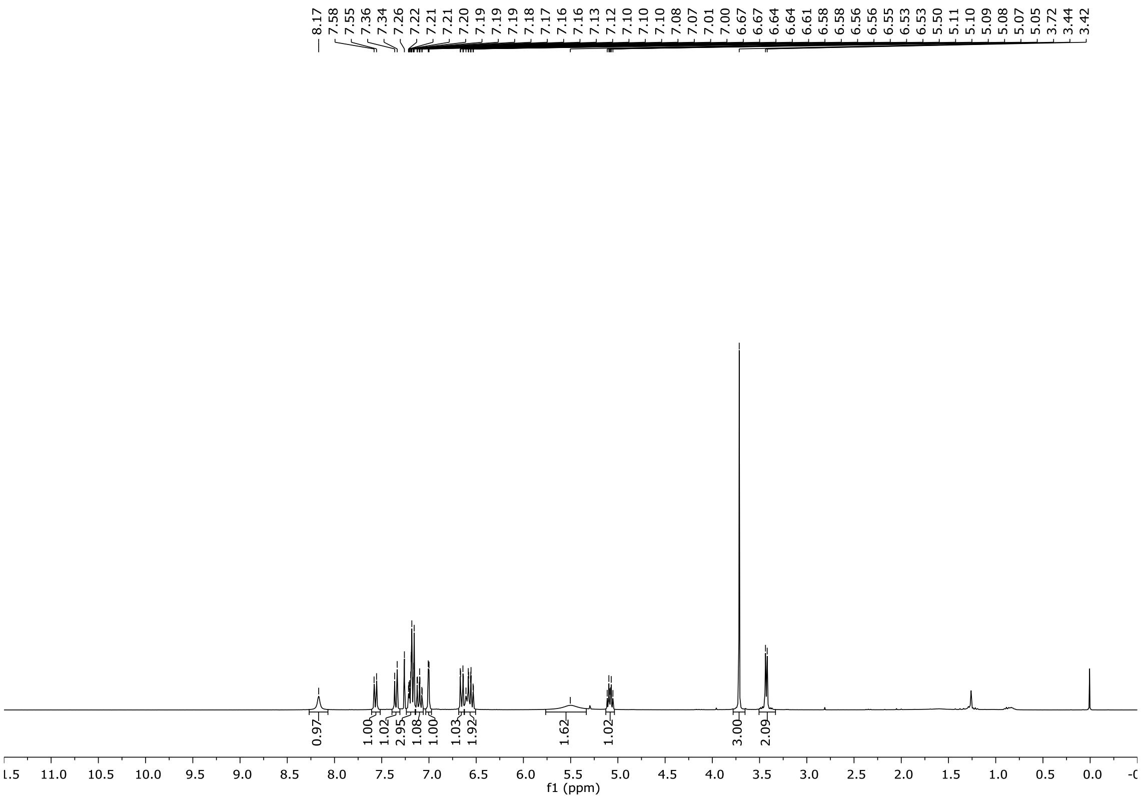

Figure S1 ^{1}H-NMR (300 MHz, $CDCl_3$) spectrum of methyl (2-aminobenzoyl)-D-tryptophanate (**8a**).

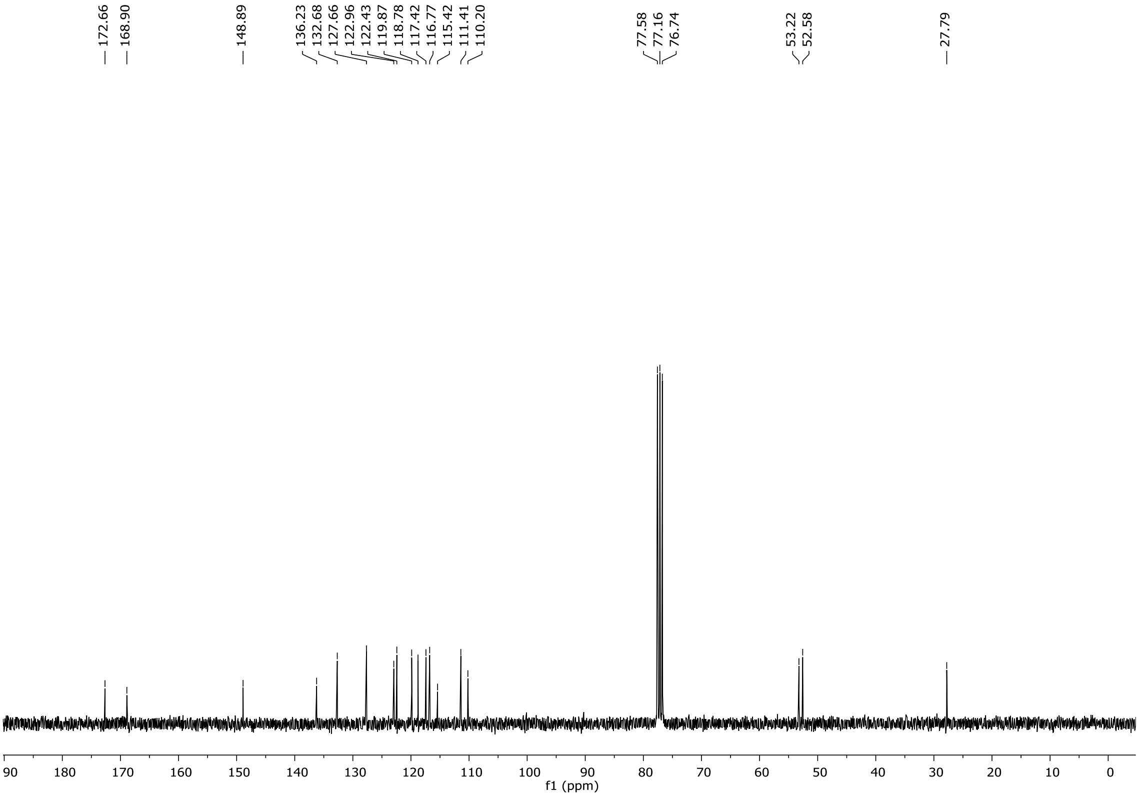

Figure S2 ^{13}C-NMR (75 MHz, $CDCl_3$) spectrum for methyl (2-aminobenzoyl)-D-tryptophanate (**8a**).

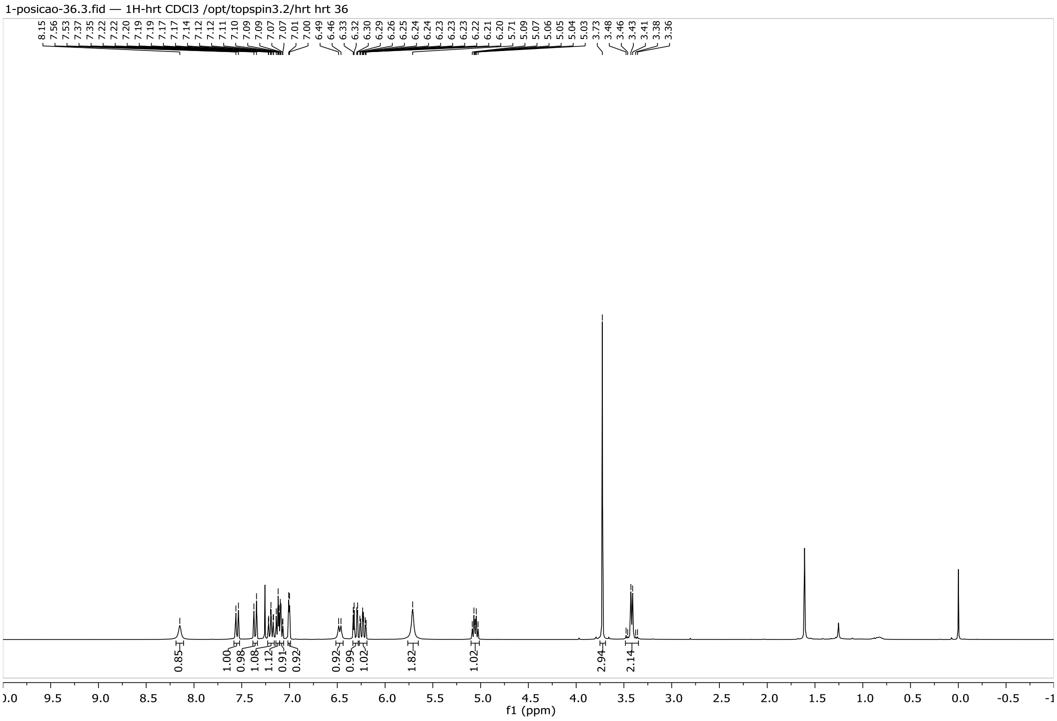

Figure S3 ^{1}H-NMR (300 MHz, $CDCl_3$) spectrum for methyl (2-amino-4-fluorobenzoyl)-D-tryptophanate (**8b**).

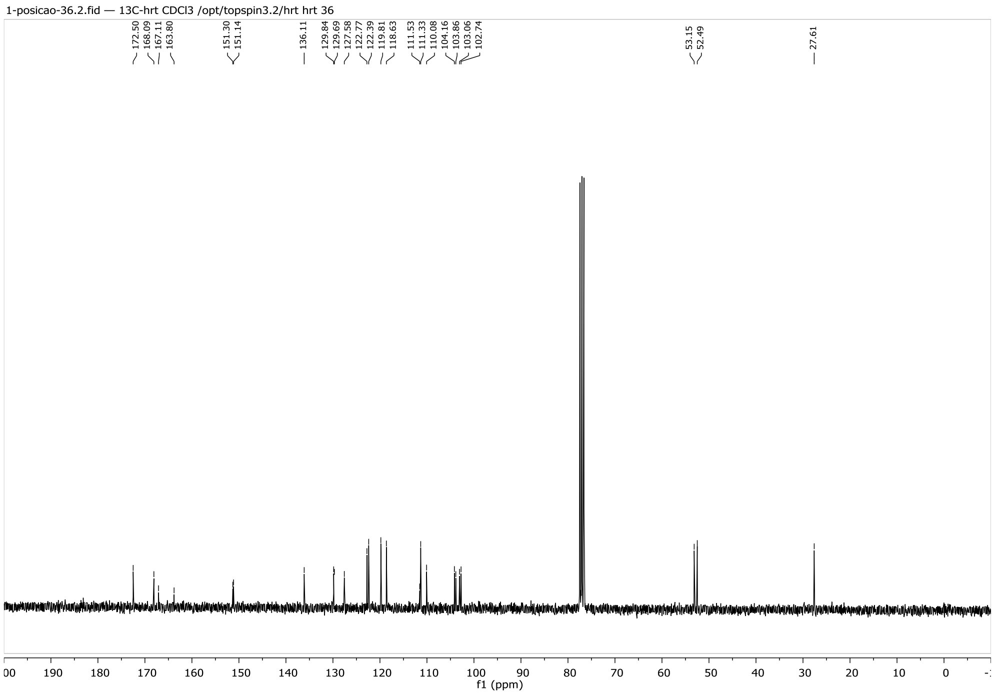

Figure S4 ^{13}C-NMR (75 MHz, $CDCl_3$) spectrum for methyl (2-amino-4-fluorobenzoyl)-D-tryptophanate (8**b**).

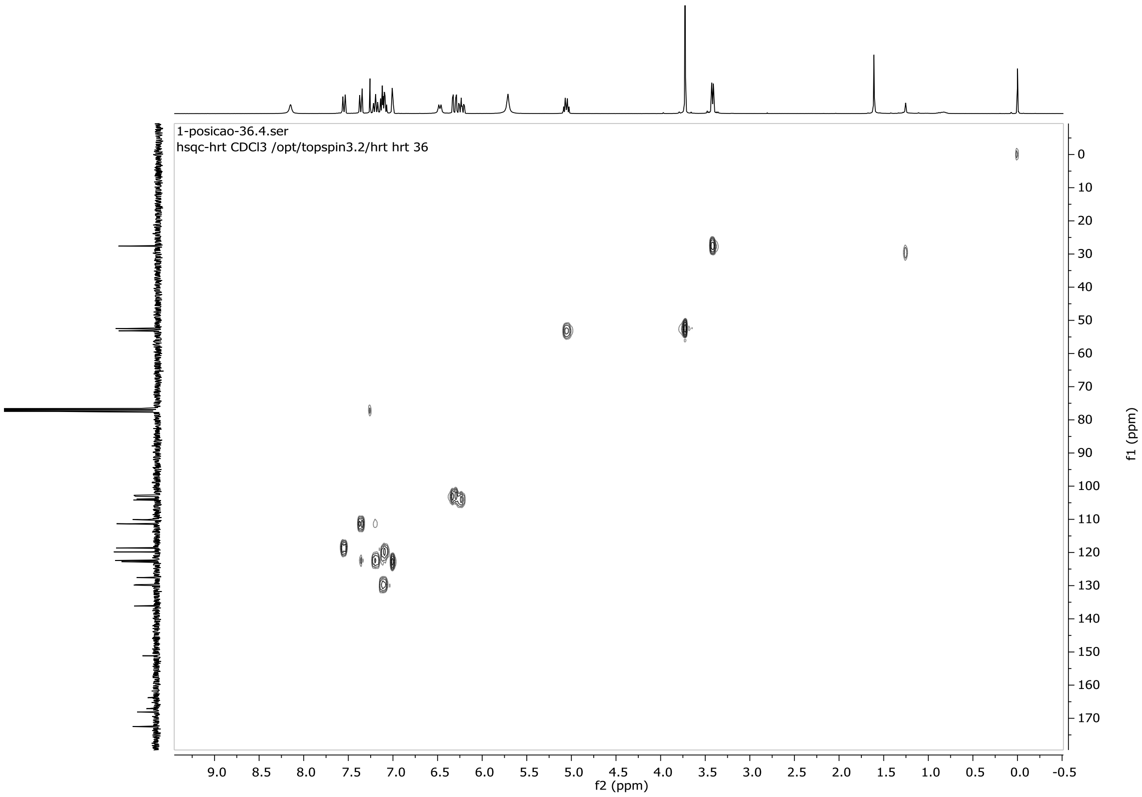

Figure S5 HSQC spectrum for methyl (2-amino-4-fluorobenzoyl)-D-tryptophanate (**8b**).

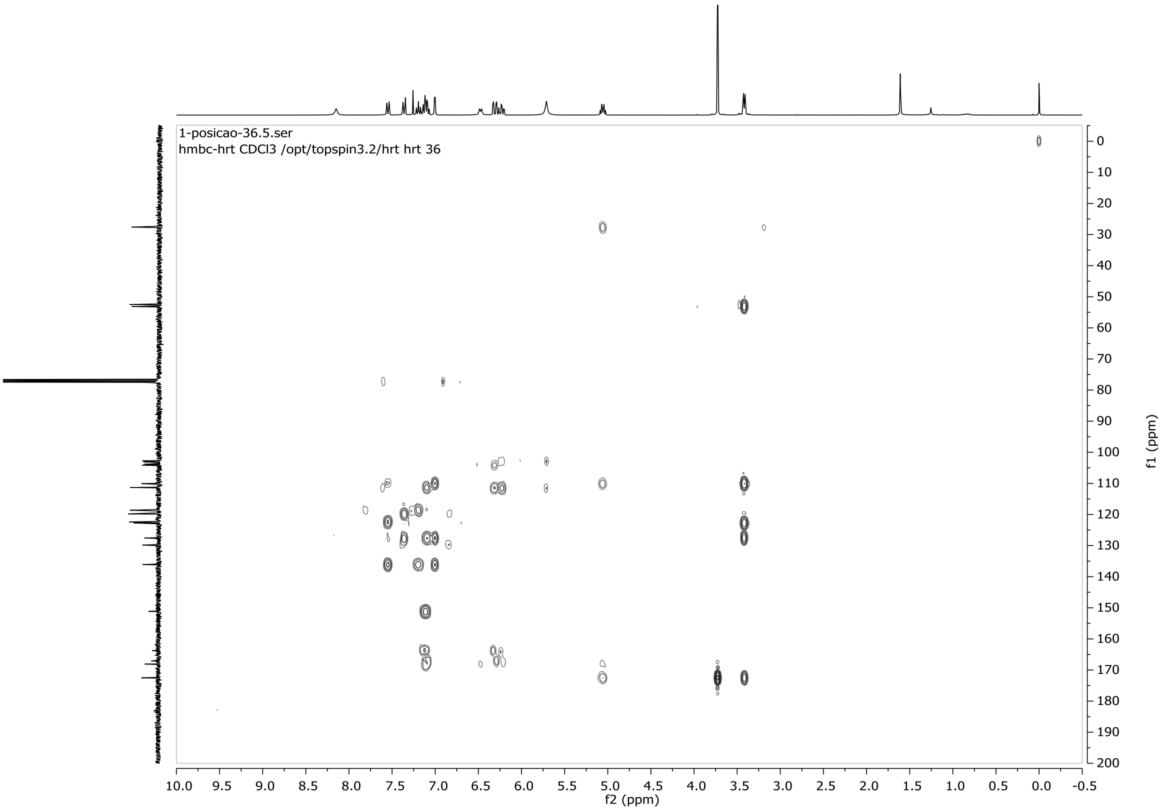

Figure S6 HMBC spectrum for methyl (2-amino-4-fluorobenzoyl)-D-tryptophanate (**8b**).

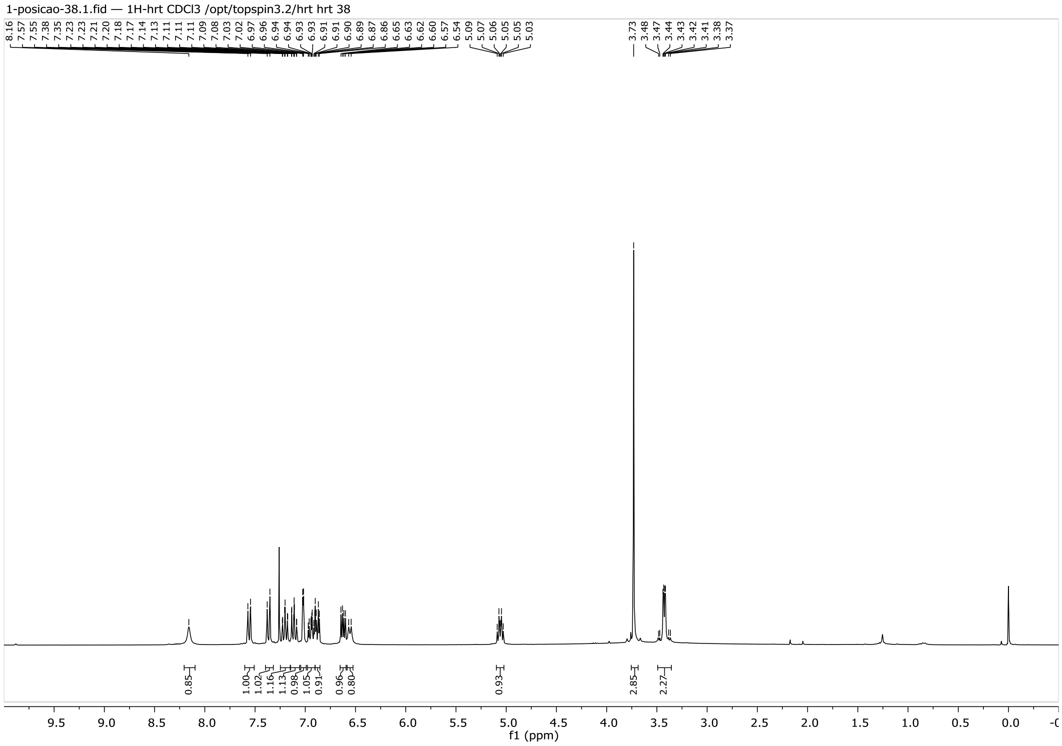

Figure S7 ^{1}H-NMR (300 MHz, $CDCl_3$) spectrum for methyl (2-amino-5-fluorobenzoyl)-D-tryptophanate (**8c**).

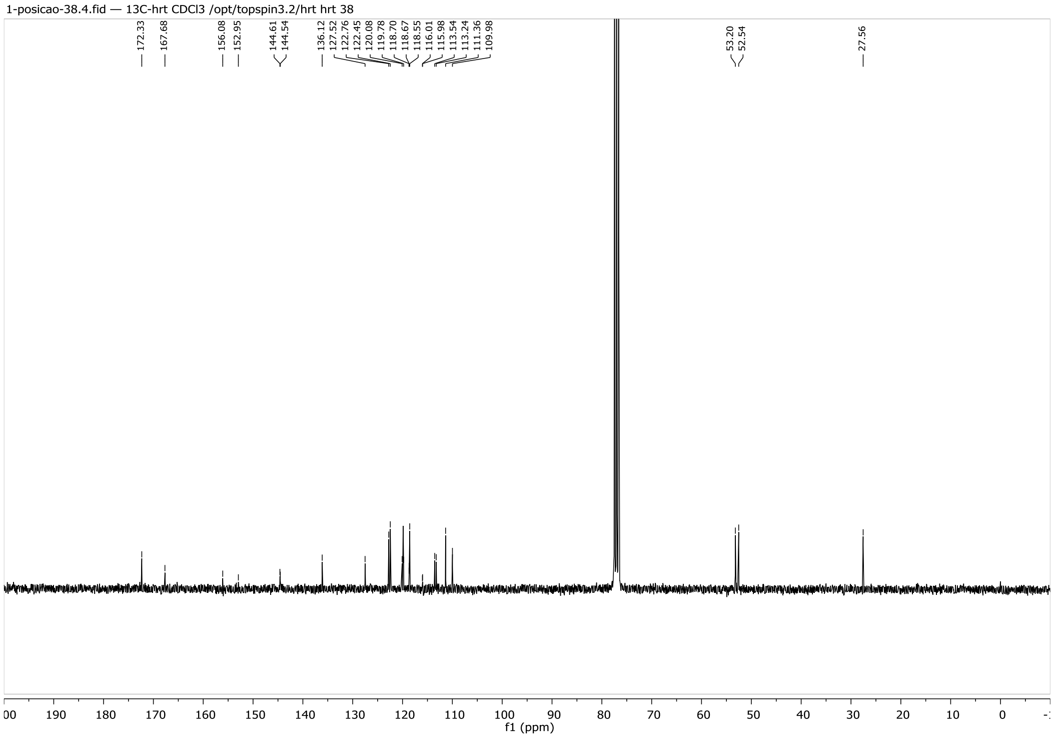

Figure S8 ^{13}C-NMR (75 MHz, $CDCl_3$) spectrum for methyl (2-amino-5-fluorobenzoyl)-D-tryptophanate (**8c**).

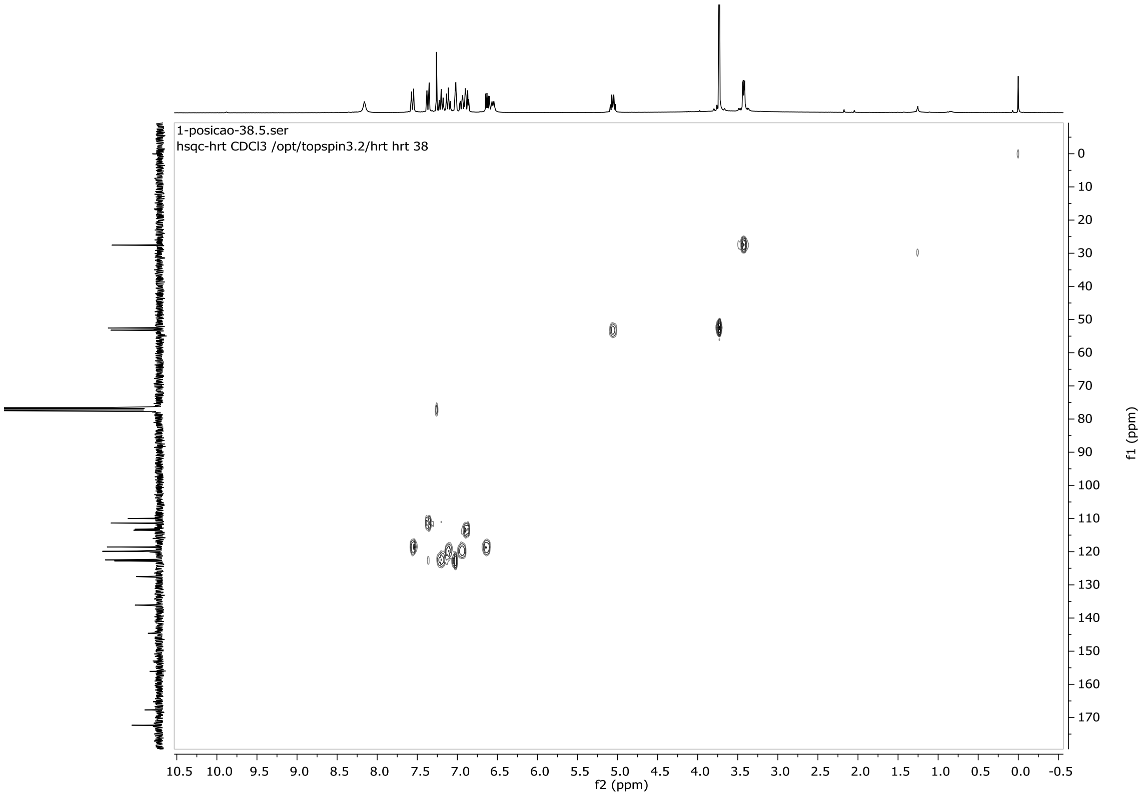

Figure S9 HSQC spectrum for methyl (2-amino-5-fluorobenzoyl)-D-tryptophanate (**8c**).

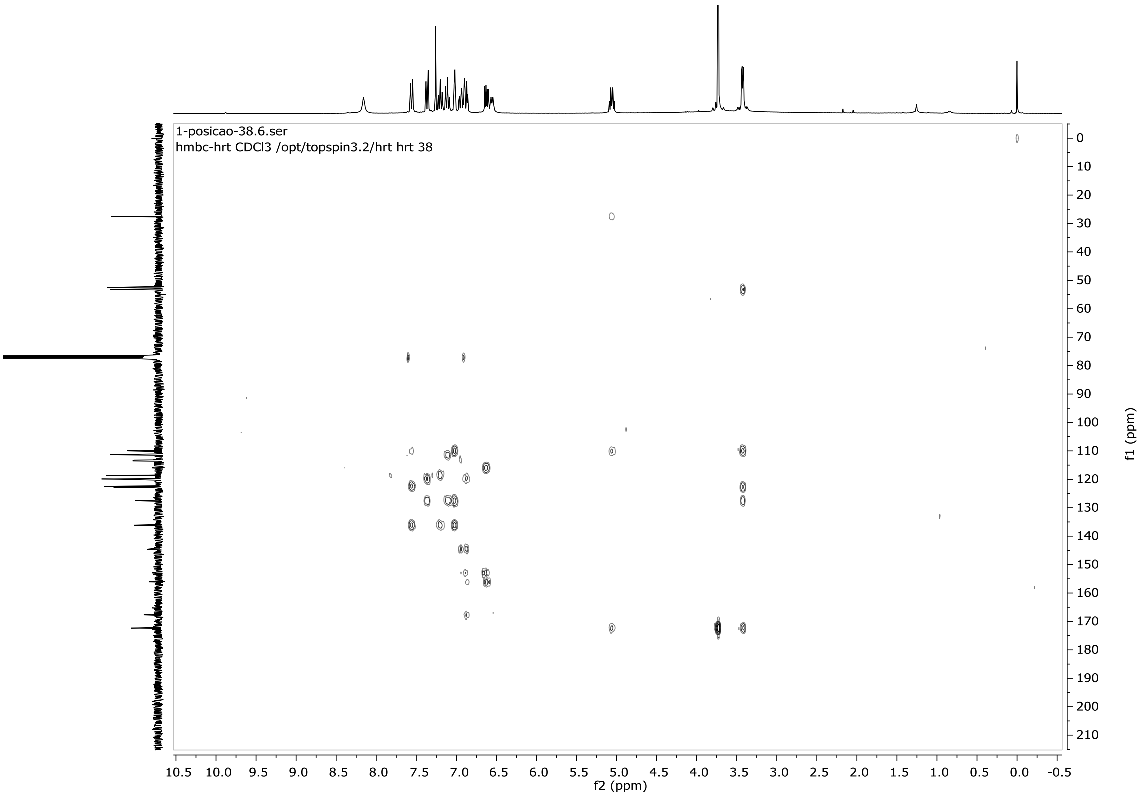

Figure S10 HMBC spectrum for methyl (2-amino-5-fluorobenzoyl)-D-tryptophanate (**8c**).

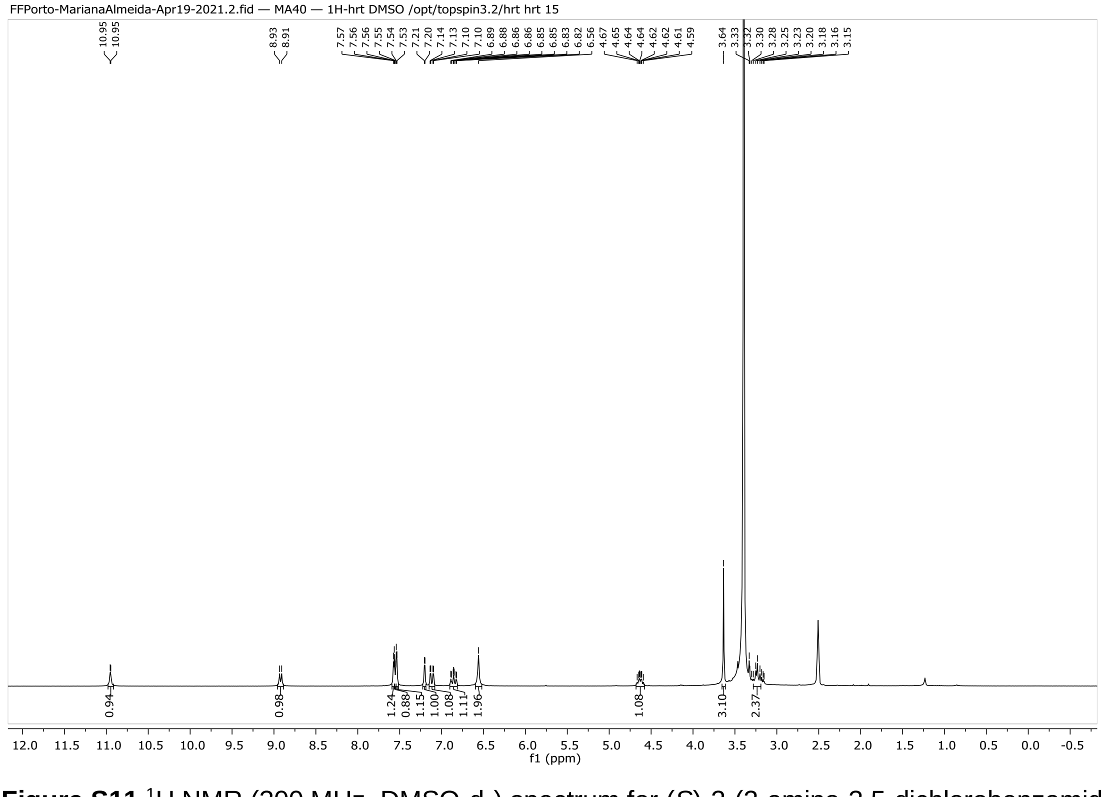

Figure S11 ^{1}H NMR (300 MHz, DMSO-d_6) spectrum for (*S*)-2-(2-amino-3,5-dichlorobenzamido)-3-(6-fluoro-1*H*-indol-3-yl)propanoate (**8d**).

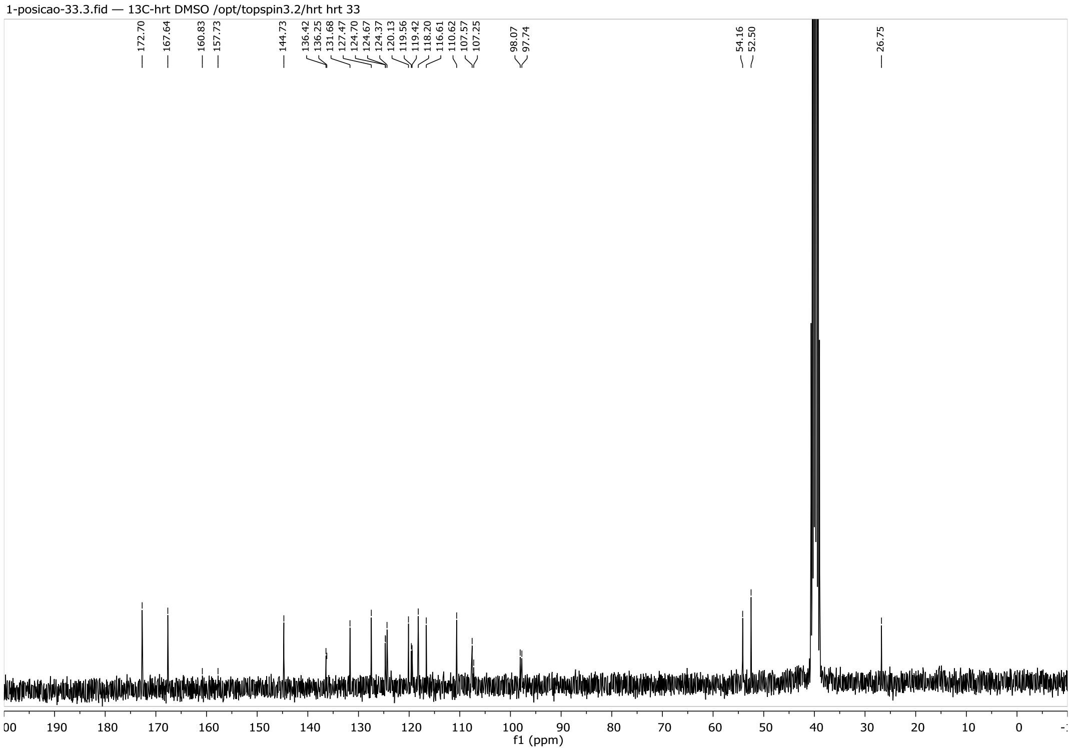

Figure S12 ^{13}C NMR (75 MHz, DMSO-d$_6$) spectrum for (*S*)-2-(2-amino-3,5-dichlorobenzamido)-3-(6-fluoro-1*H*-indol-3-yl)propanoate (**8d**).

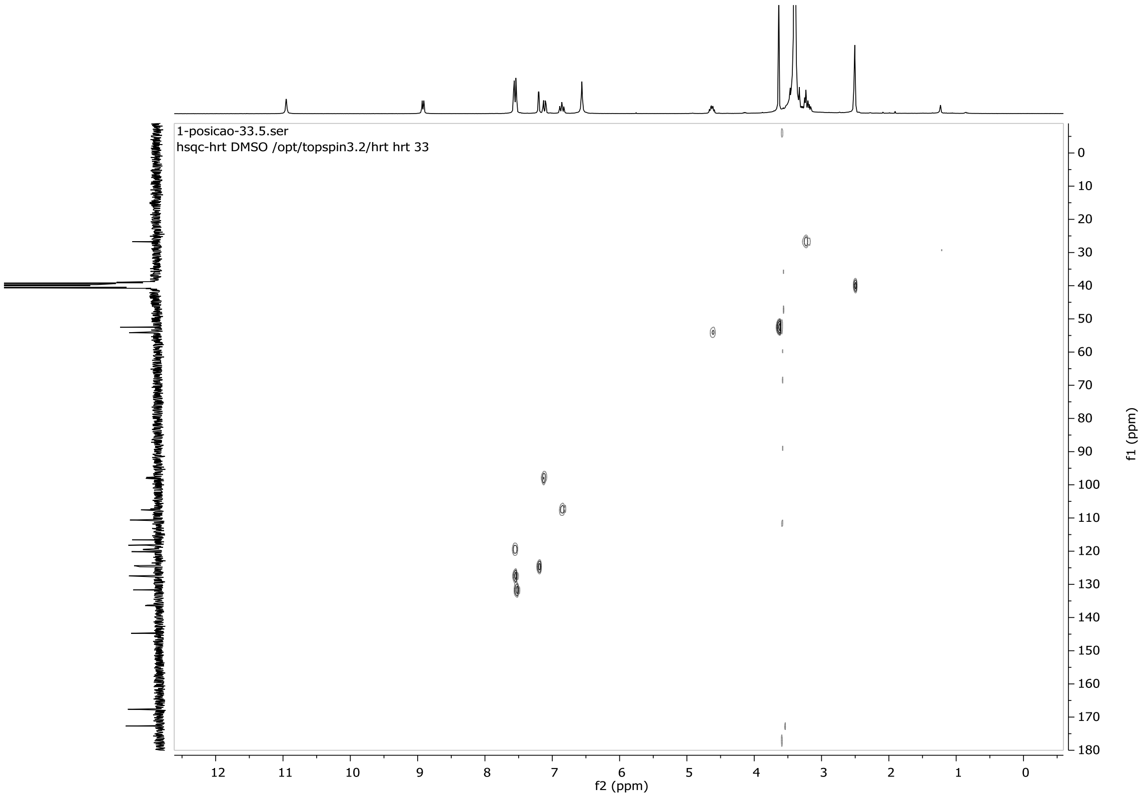

Figure S13 HSQC spectrum for (*S*)-2-(2-amino-3,5-dichlorobenzamido)-3-(6-fluoro-1*H*-indol-3-yl)propanoate (**8d**).

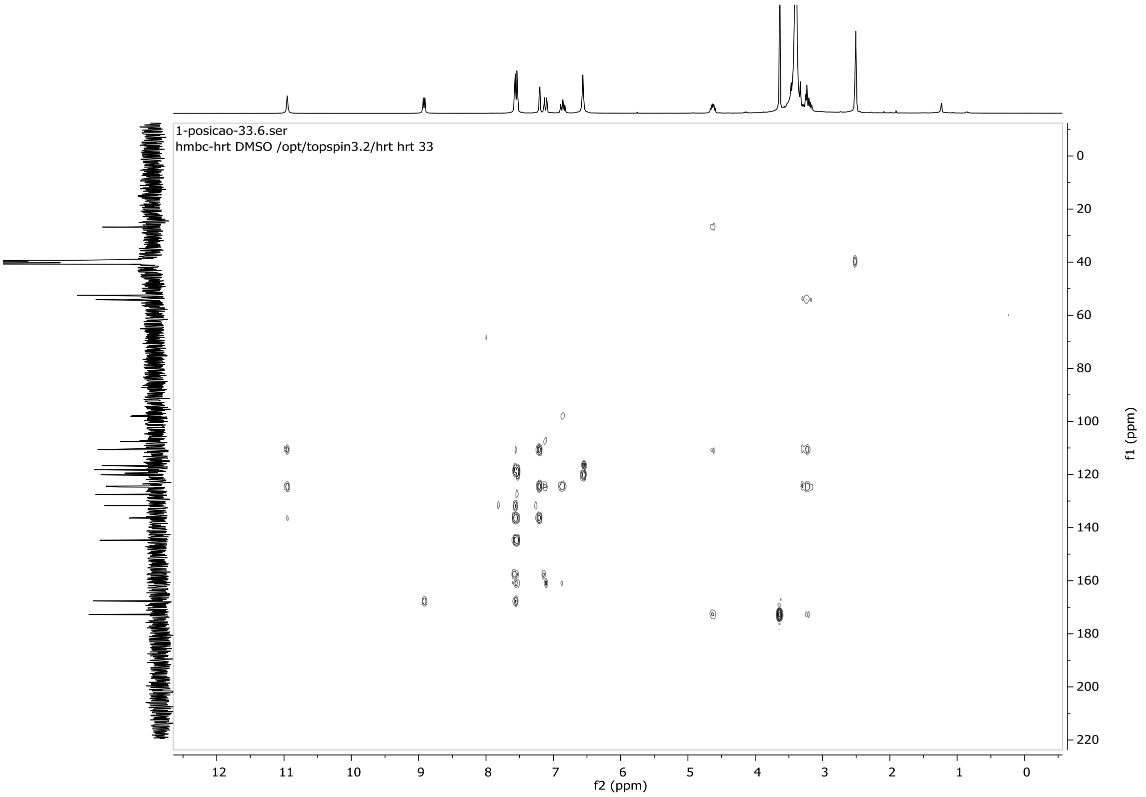

Figure S14 HMBC spectrum for (*S*)-2-(2-amino-3,5-dichlorobenzamido)-3-(6-fluoro-1*H*-indol-3-yl)propanoate (**8d**).

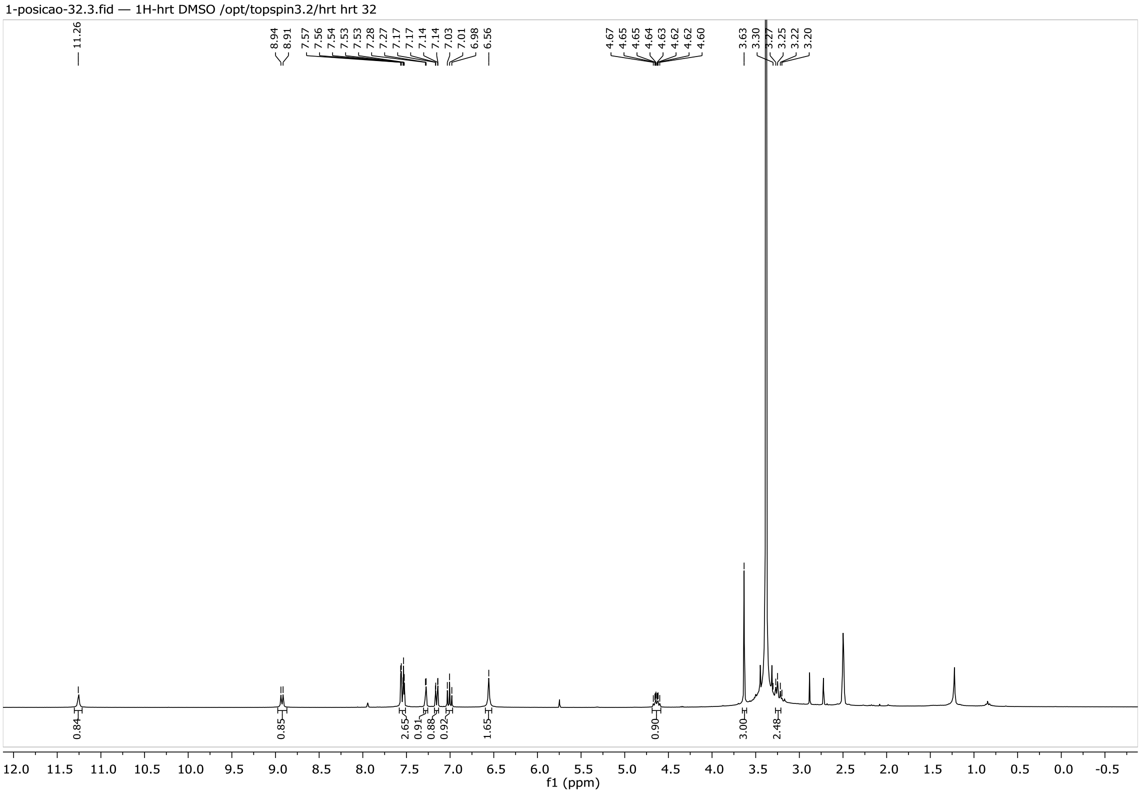

Figure S15 ^{1}H NMR (300 MHz, DMSO-d_6) spectrum for (*S*)-2-(2-amino-3,5-dichlorobenzamido)-3-(7-chloro-1*H*-indol-3-yl)propanoate (**8e**).

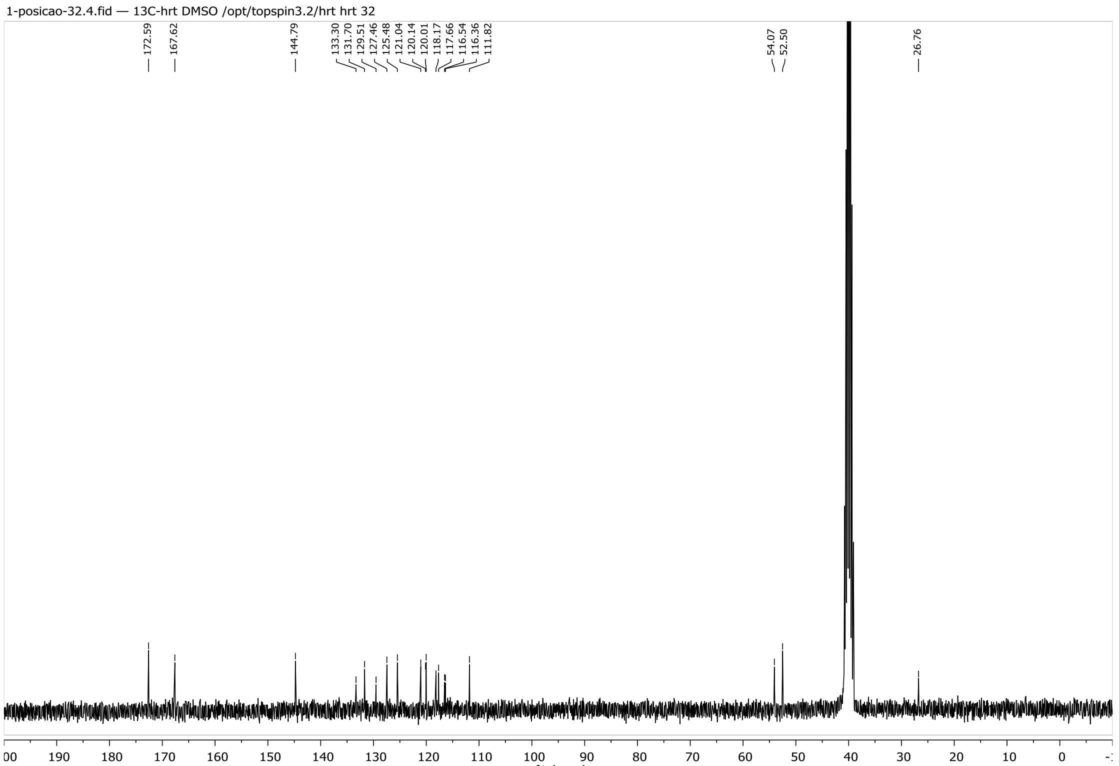

Figure S16 ^{13}C NMR (75 MHz, DMSO-d_6) spectrum for (*S*)-2-(2-amino-3,5-dichlorobenzamido)-3-(7-chloro-1*H*-indol-3-yl)propanoate (**8e**).

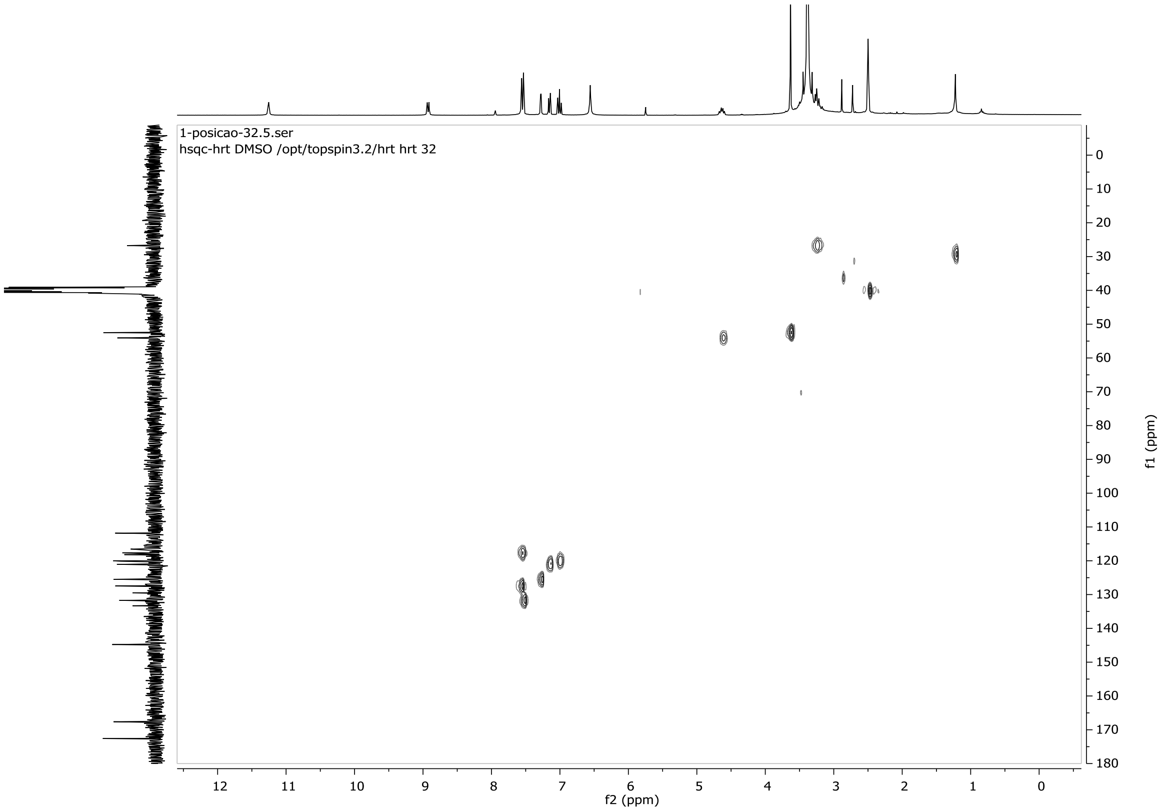

Figure S17 HSQC spectrum for (*S*)-2-(2-amino-3,5-dichlorobenzamido)-3-(7-chloro-1*H*-indol-3-yl)propanoate (**8e**).

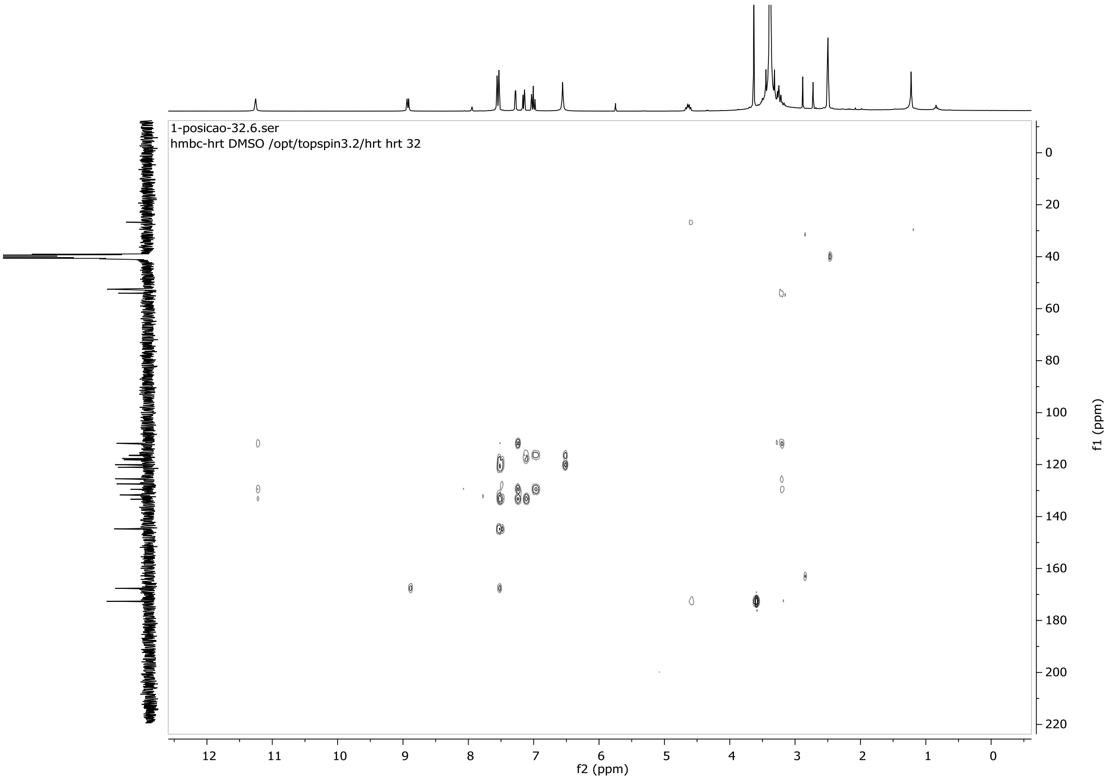

Figure S18 HMBC spectrum for (*S*)-2-(2-amino-3,5-dichlorobenzamido)-3-(7-chloro-1*H*-indol-3-yl)propanoate (**8e**).

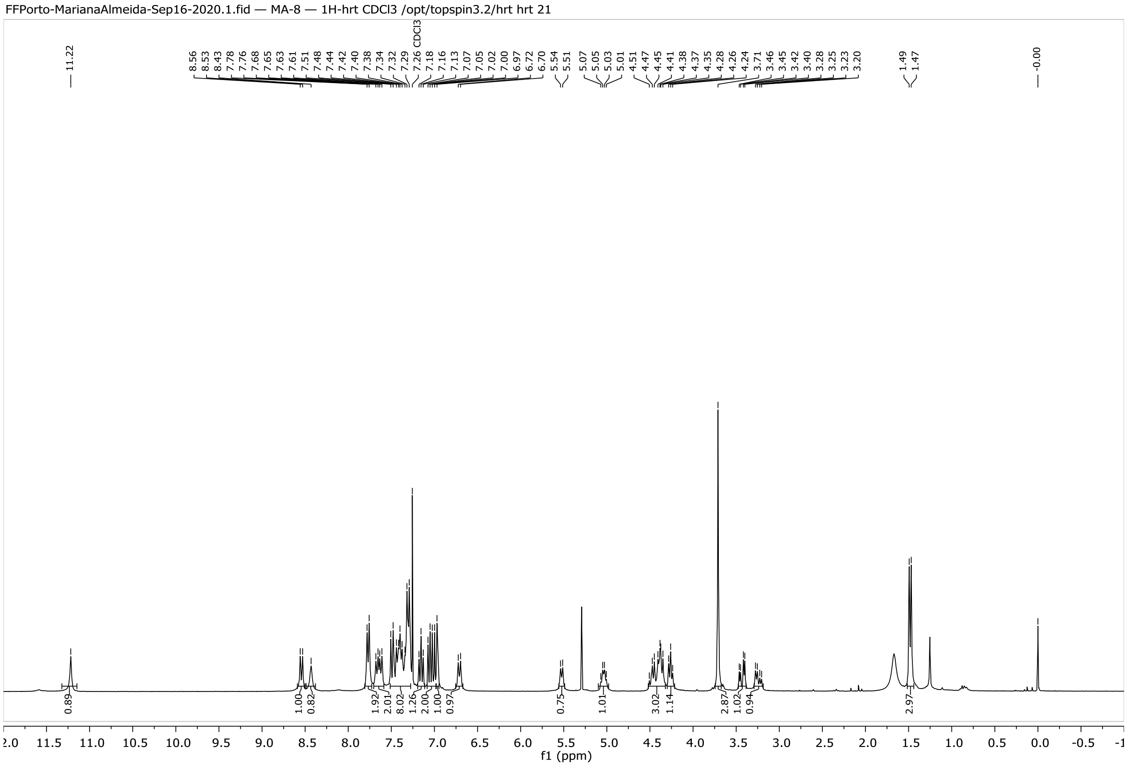

Figure S19 ¹H-NMR (300 MHz, $CDCl_3$) spectrum of methyl (2-((S)-2-((((9H-fluoren-9-yl)methoxy)carbonyl)amino)propanamido)benzoyl)-D-tryptophanate (**11b**).

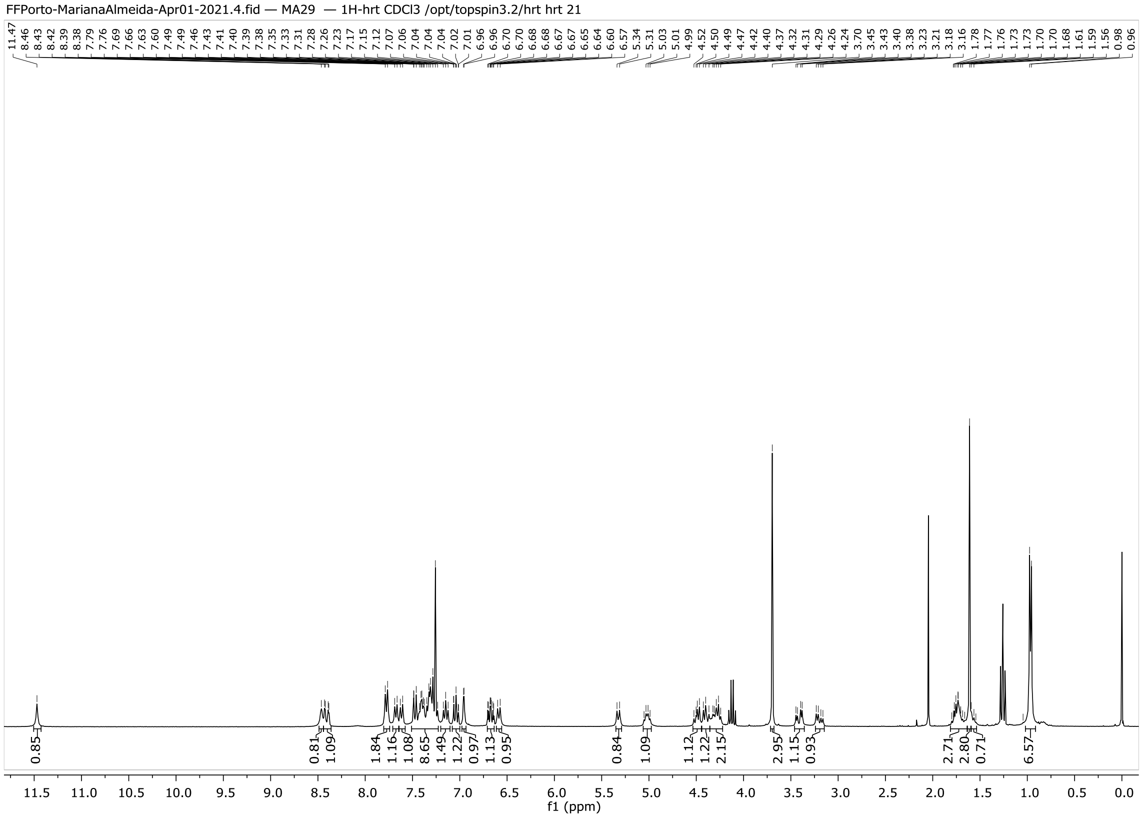

Figure S20 ^{1}H-NMR (300 MHz, $CDCl_3$) spectrum for methyl (2-((*S*)-2-((((9*H*-fluoren-9-yl)methoxy)carbonyl)amino)-4-methylpentanamido)-4-fluorobenzoyl)-D-tryptophanate (**11c**).

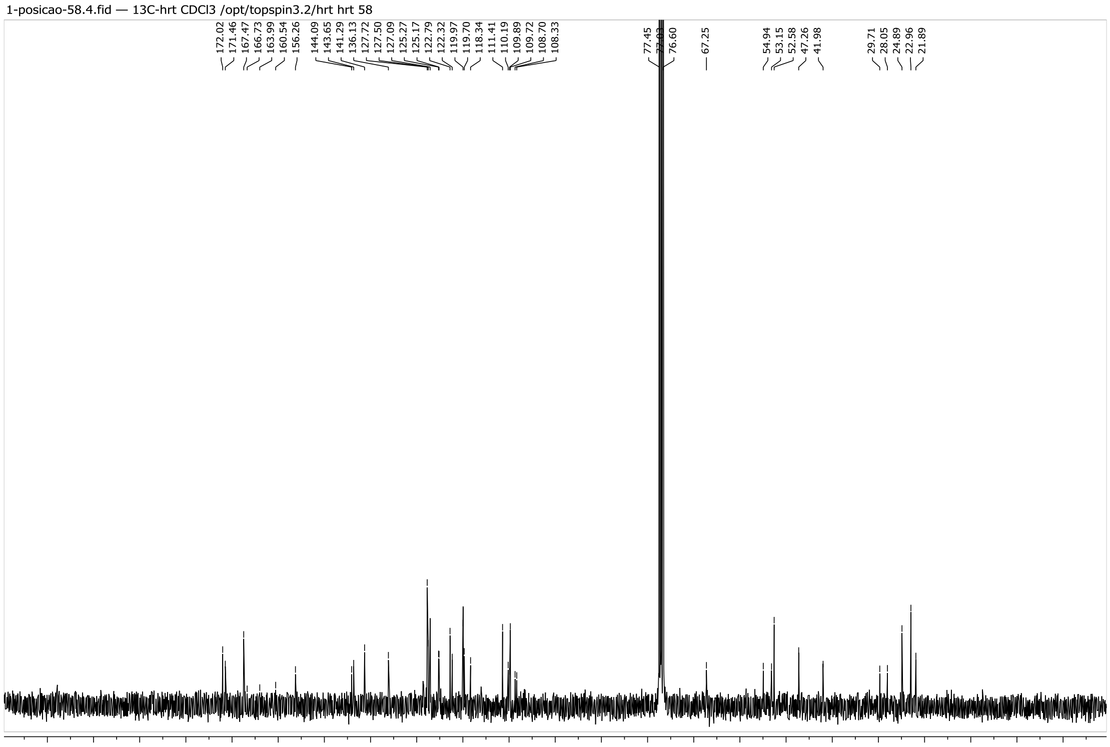

Figure S21 ^{13}C-NMR (75 MHz, $CDCl_3$) spectrum for methyl (2-((*S*)-2-((((9*H*-fluoren-9-yl)methoxy)carbonyl)amino)-4-methylpentanamido)-4-fluorobenzoyl)-D-tryptophanate (**11c**).

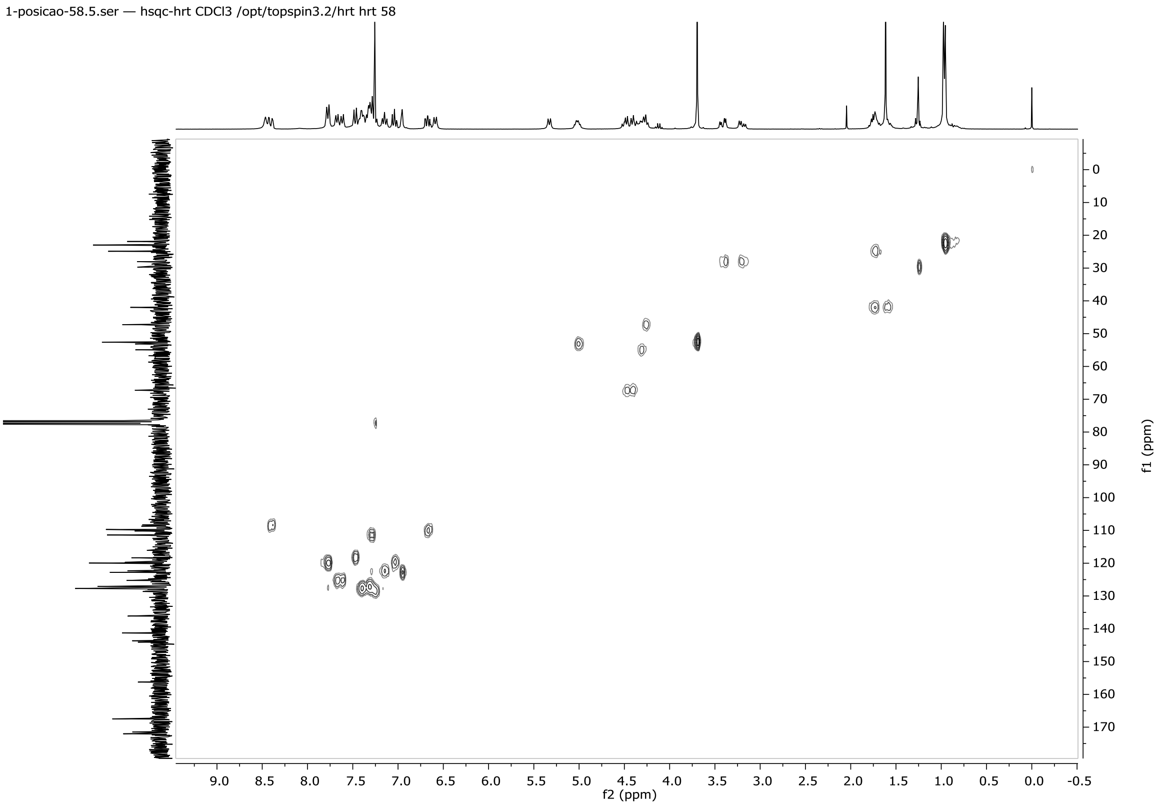

Figure S22 HSQC spectrum for methyl (2-((*S*)-2-((((9*H*-fluoren-9-yl)methoxy)carbonyl)amino)-4-methylpentanamido)-4-fluorobenzoyl)-D-tryptophanate (**11c**).

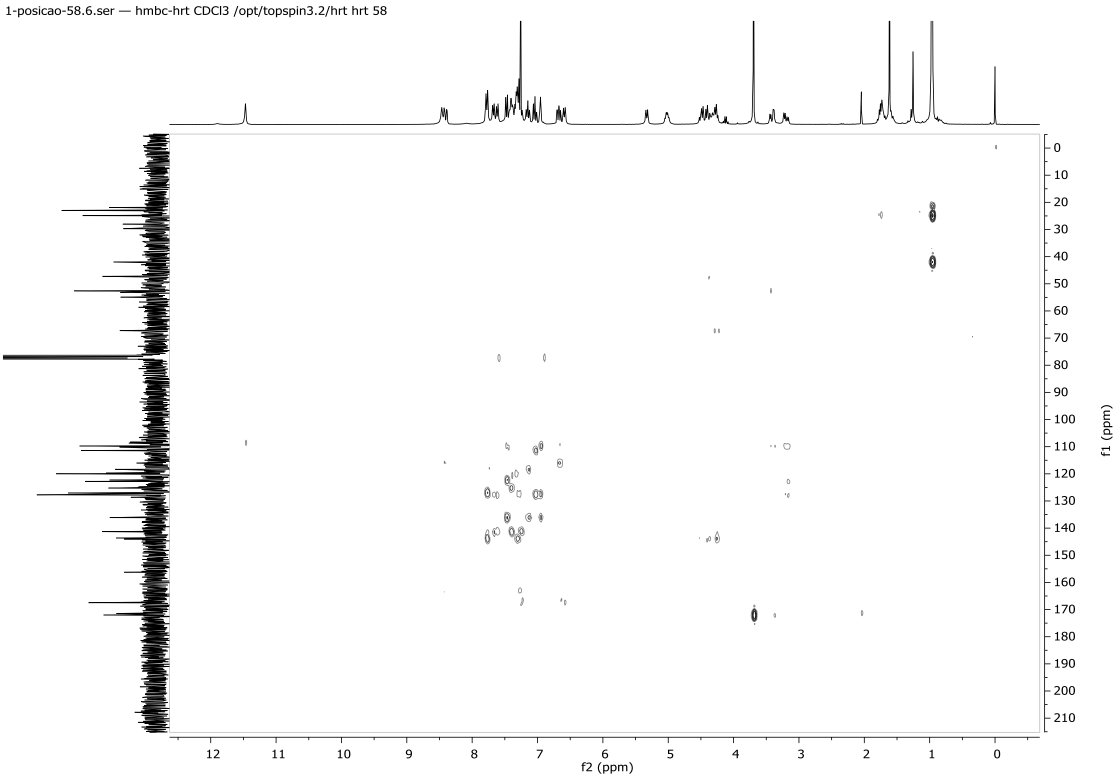

Figure S23 HMBC spectrum for methyl (2-((*S*)-2-((((9*H*-fluoren-9-yl)methoxy)carbonyl)amino)-4-methylpentanamido)-4-fluorobenzoyl)-D-tryptophanate (**11c**).

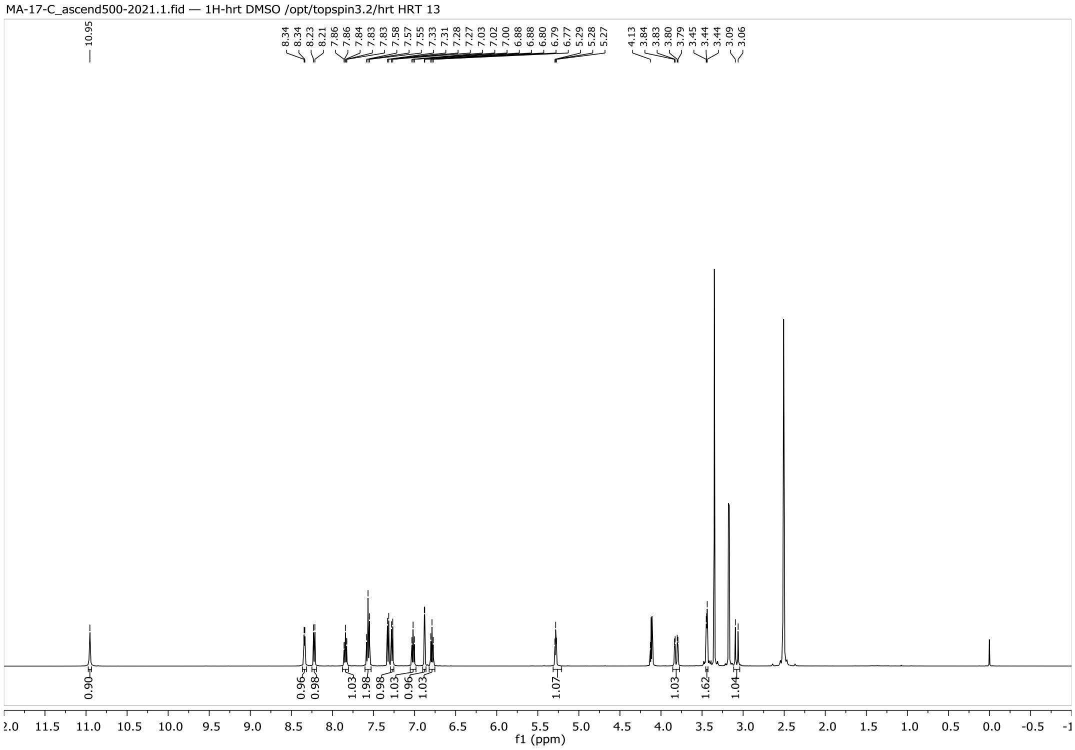

Figure S24 ^{1}H NMR (500 MHz, DMSO-d$_6$) spectrum for (*R*)-4-((1*H*-indol-3-yl)methyl)-1,2-dihydro-6*H*-pyrazino[2,1-*b*]quinazoline-3,6(4*H*)-dione (**1, Glyantrypine**).

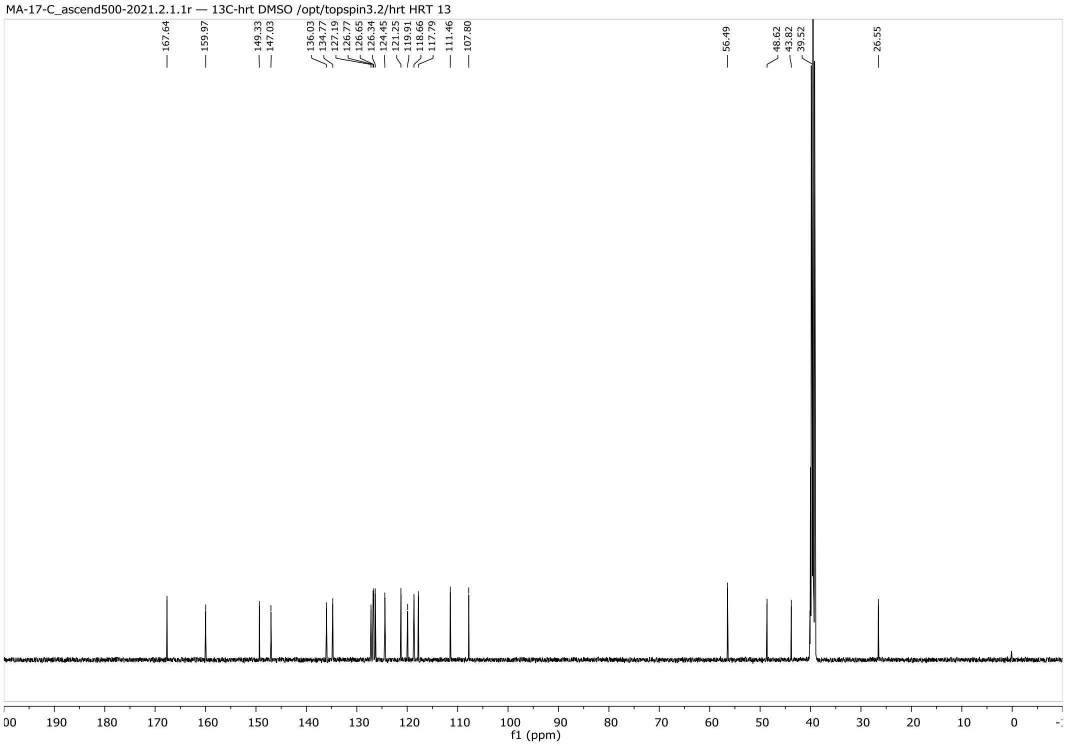

Figure S25 ^{13}C NMR (125 MHz, DMSO-d$_6$) spectrum for (*R*)-4-((1*H*-indol-3-yl)methyl)-1,2-dihydro-6*H*-pyrazino[2,1-*b*]quinazoline-3,6(4*H*)-dione (**1, Glyantrypine**).

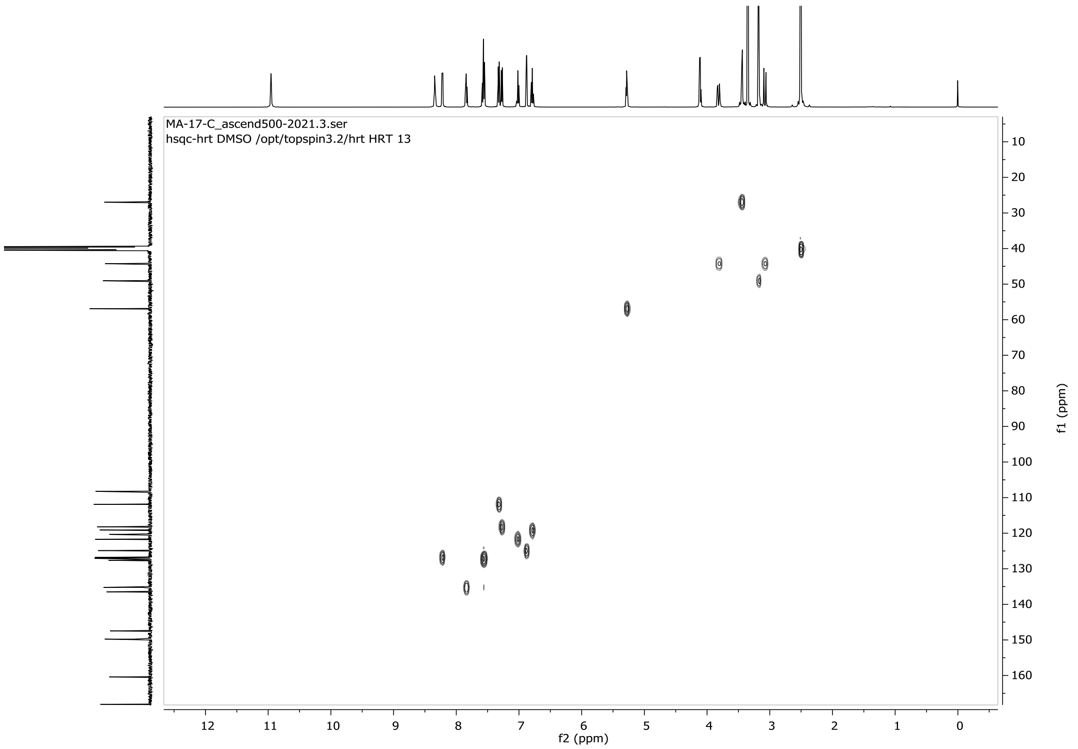

Figure S26 HSQC spectrum for (*R*)-4-((1*H*-indol-3-yl)methyl)-1,2-dihydro-6*H*-pyrazino[2,1-*b*]quinazoline-3,6(4*H*)-dione (**1, Glyantrypine**).

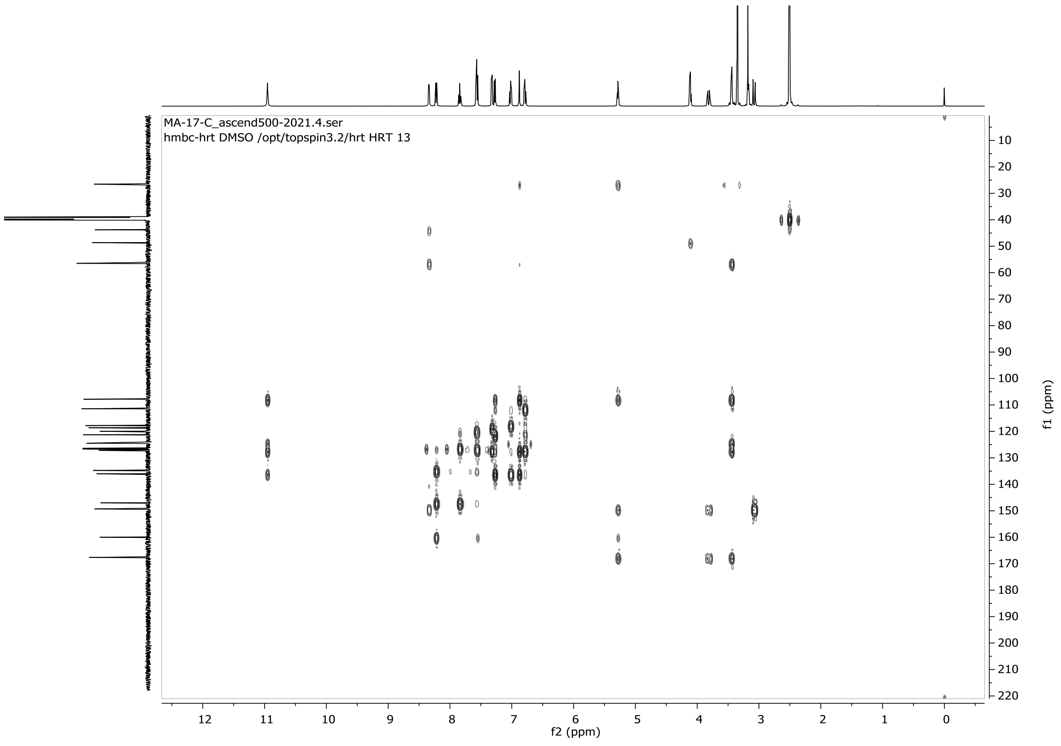

Figure S27 HMBC spectrum for (*R*)-4-((1*H*-indol-3-yl)methyl)-1,2-dihydro-6*H*-pyrazino[2,1-*b*]quinazoline-3,6(4*H*)-dione (**1, Glyantrypine**).

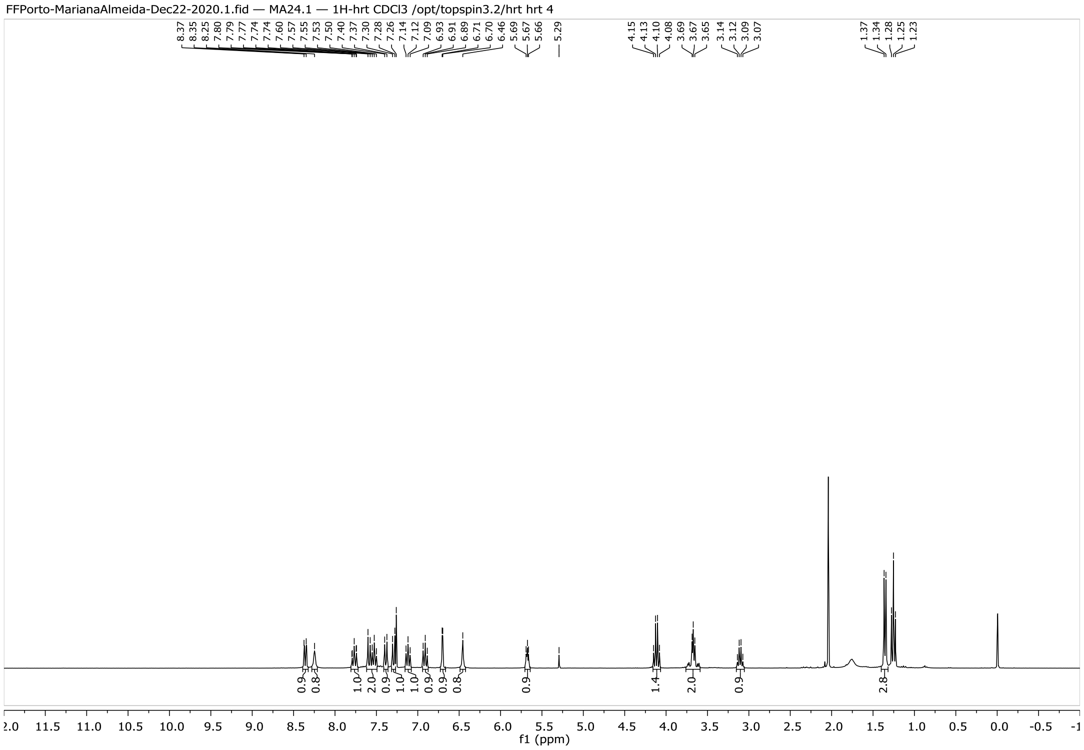

Figure S28 ^{1}H-NMR (300 MHz, $CDCl_3$) spectrum for (1*S*,4*R*)-4-((1*H*-indol-3-yl)methyl)-1-methyl-1,2-dihydro-6*H*-pyrazino[2,1-*b*]quinazoline-3,6(4*H*)-dione (**2, Fumiquinazoline F**).

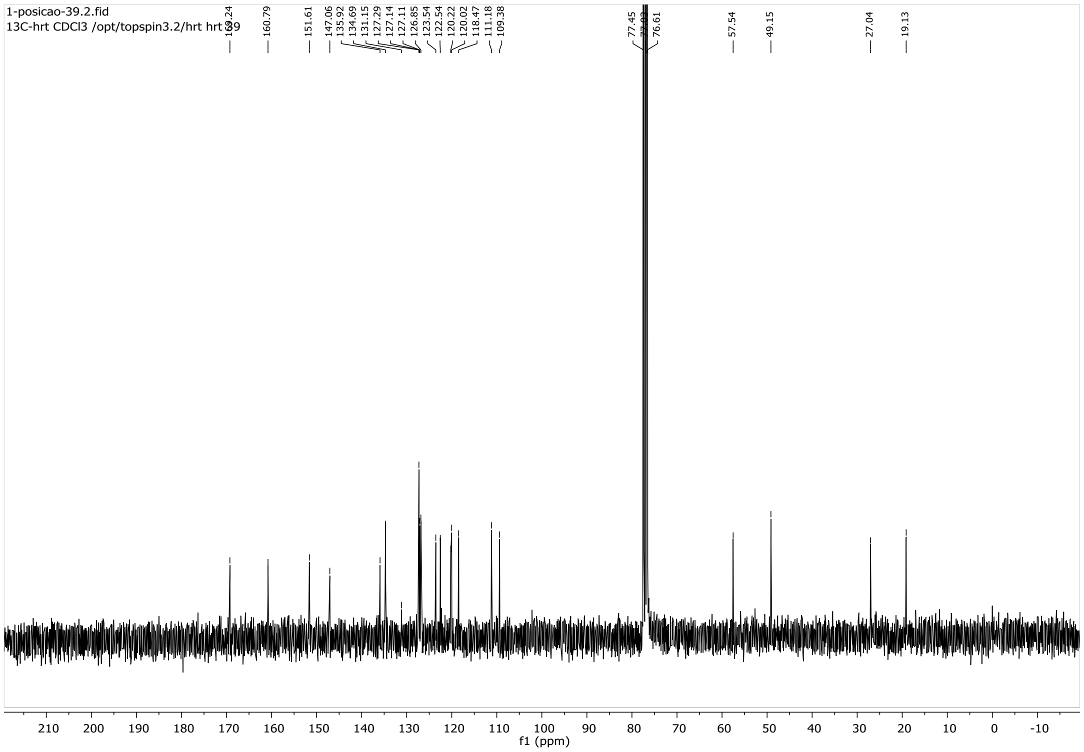

Figure S29 ^{1}C-NMR (75 MHz, $CDCl_3$) spectrum for (1*S*,4*R*)-4-((*1H*-indol-3-yl)methyl)-1-methyl-1,2-dihydro-6*H*-pyrazino[2,1-*b*]quinazoline-3,6(4*H*)-dione (**2, Fumiquinazoline F**).

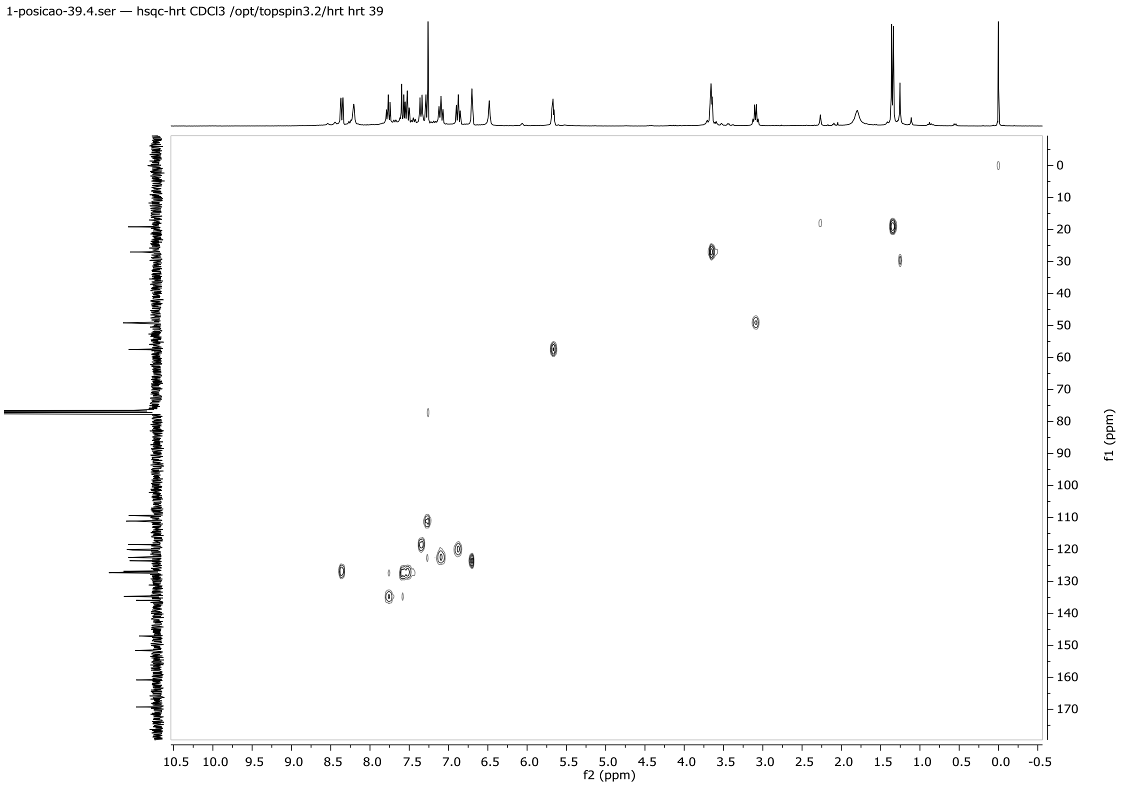

Figure S30 HSQC spectrum for (1*S*,4*R*)-4-((1*H*-indol-3-yl)methyl)-1-methyl-1,2-dihydro-6*H*-pyrazino[2,1-*b*]quinazoline-3,6(4*H*)-dione (**2, Fumiquinazoline F**).

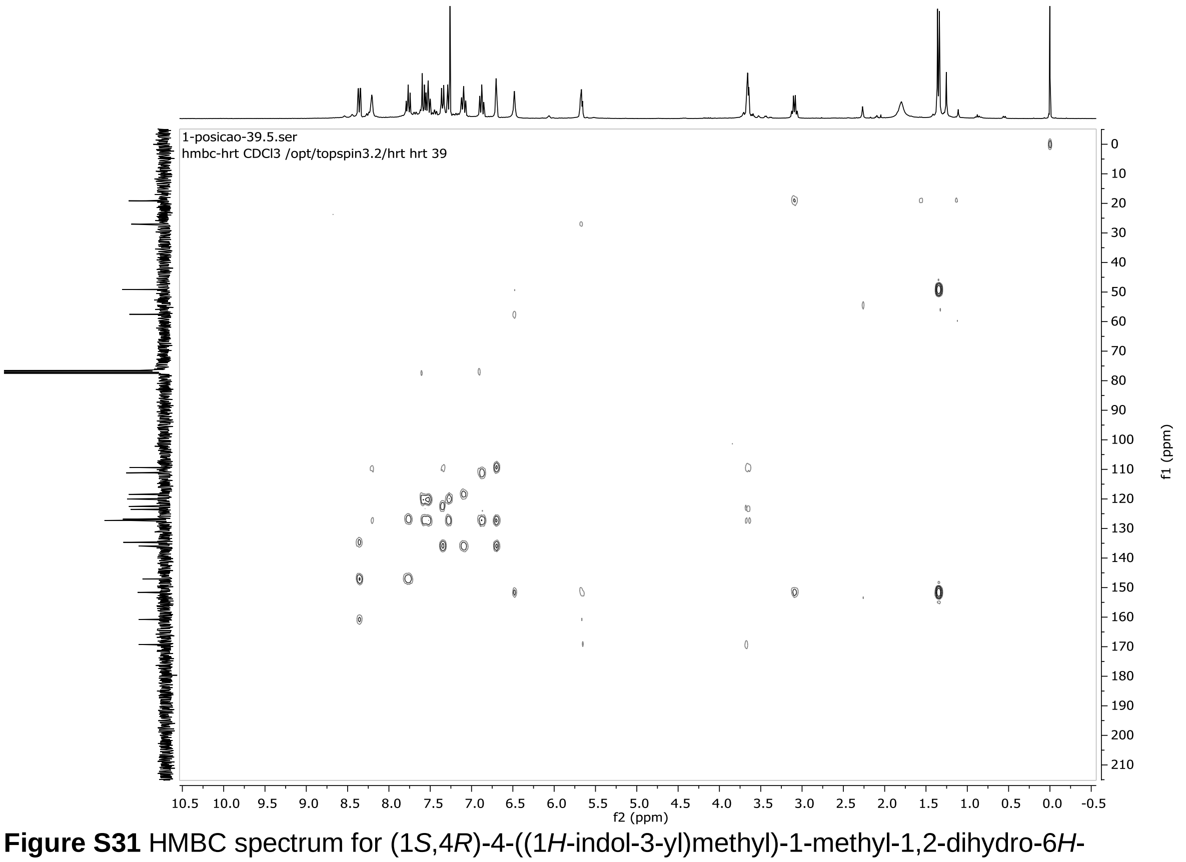

Figure S31 HMBC spectrum for (1*S*,4*R*)-4-((1*H*-indol-3-yl)methyl)-1-methyl-1,2-dihydro-6*H*-pyrazino[2,1-*b*]quinazoline-3,6(4*H*)-dione (**2, Fumiquinazoline F**).

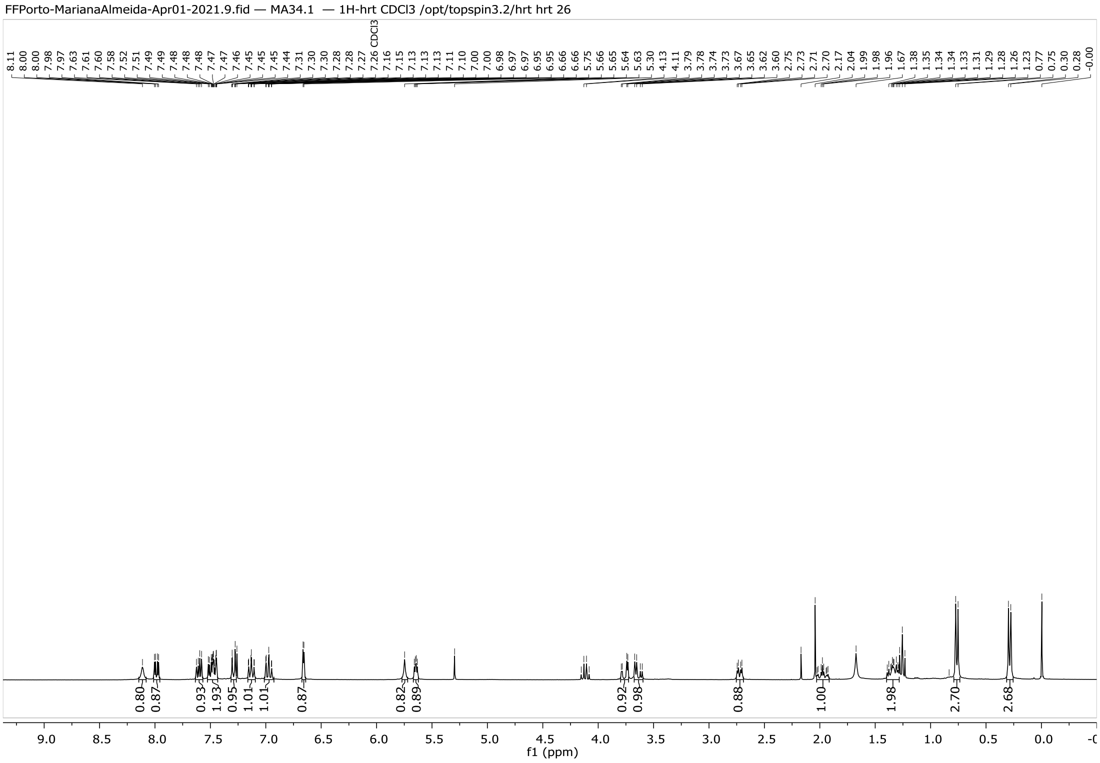

Figure S32 ^{1}H NMR (300 MHz, $CDCl_3$) spectrum for (1*S*,4*R*)-4-((1*H*-indol-3-yl)methyl)-8-fluoro-1-isobutyl-1,2-dihydro-6*H*-pyrazino[2,1-*b*]quinazoline-3,6(4*H*)-dione (**12a**).

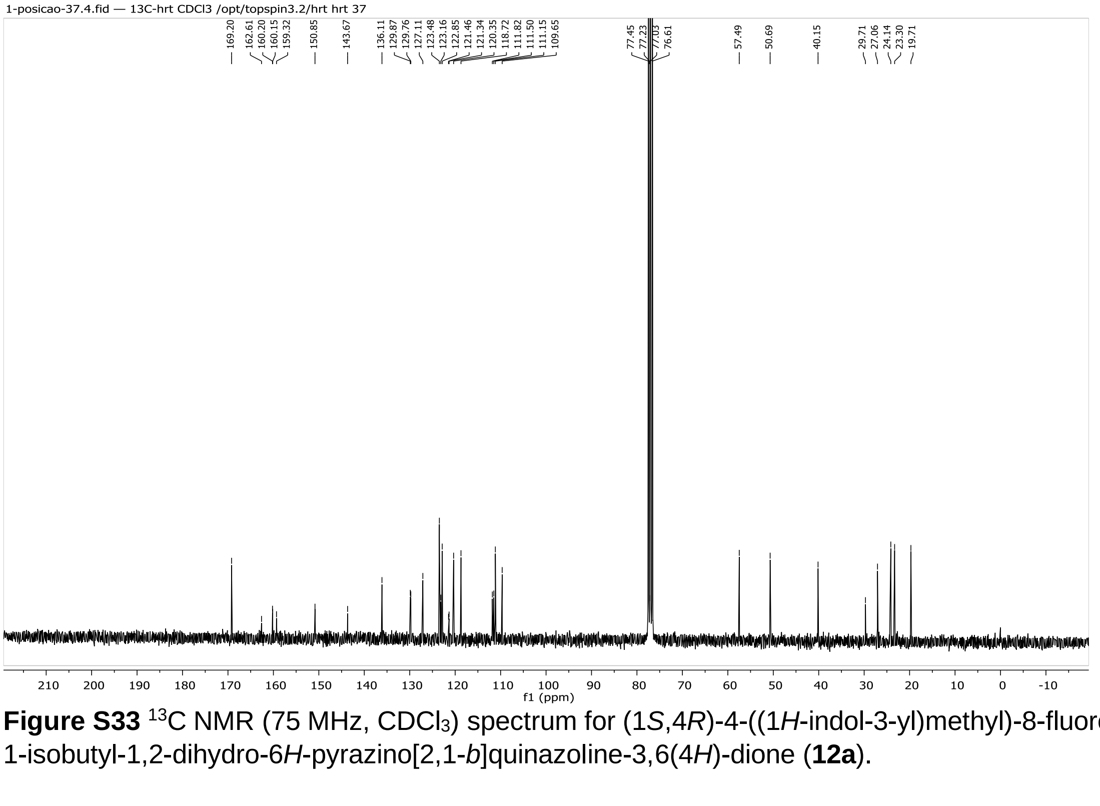

Figure S33 ^{13}C NMR (75 MHz, $CDCl_3$) spectrum for (1*S*,4*R*)-4-((1*H*-indol-3-yl)methyl)-8-fluoro-1-isobutyl-1,2-dihydro-6*H*-pyrazino[2,1-*b*]quinazoline-3,6(4*H*)-dione (**12a**).

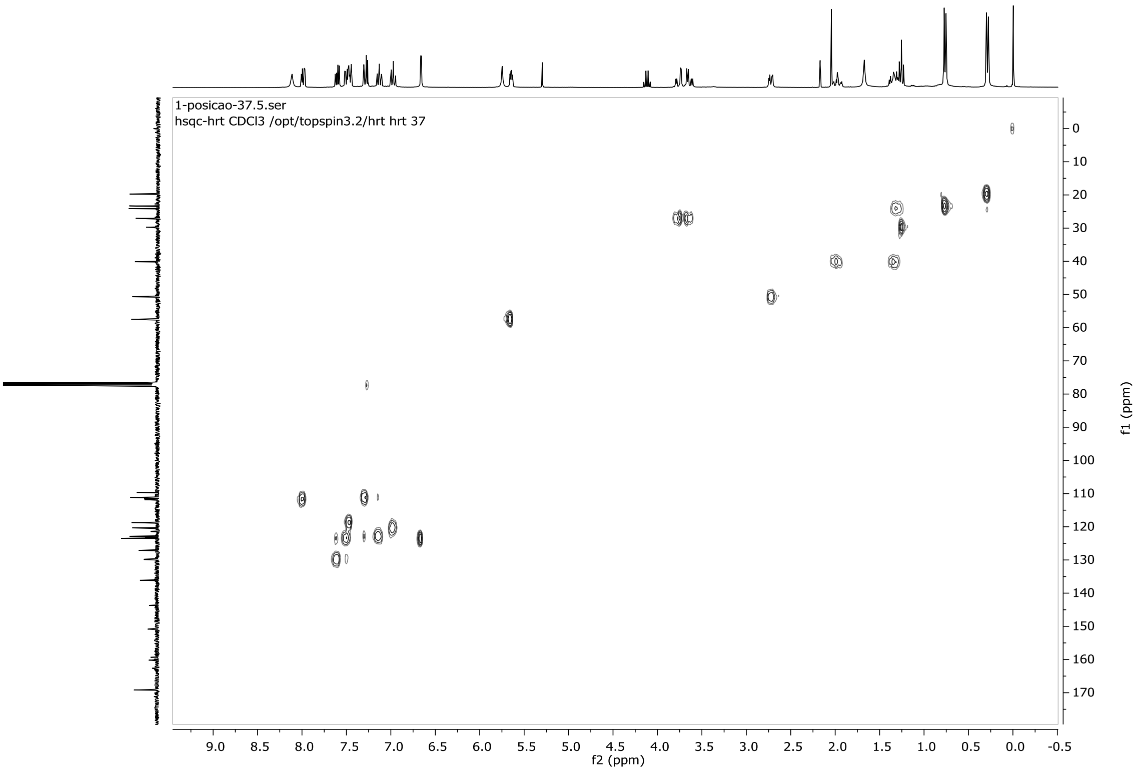

Figure S34 HSQC spectrum for (1*S*,4*R*)-4-((1*H*-indol-3-yl)methyl)-8-fluoro-1-isobutyl-1,2-dihydro-6*H*-pyrazino[2,1-*b*]quinazoline-3,6(4*H*)-dione (**12a**).

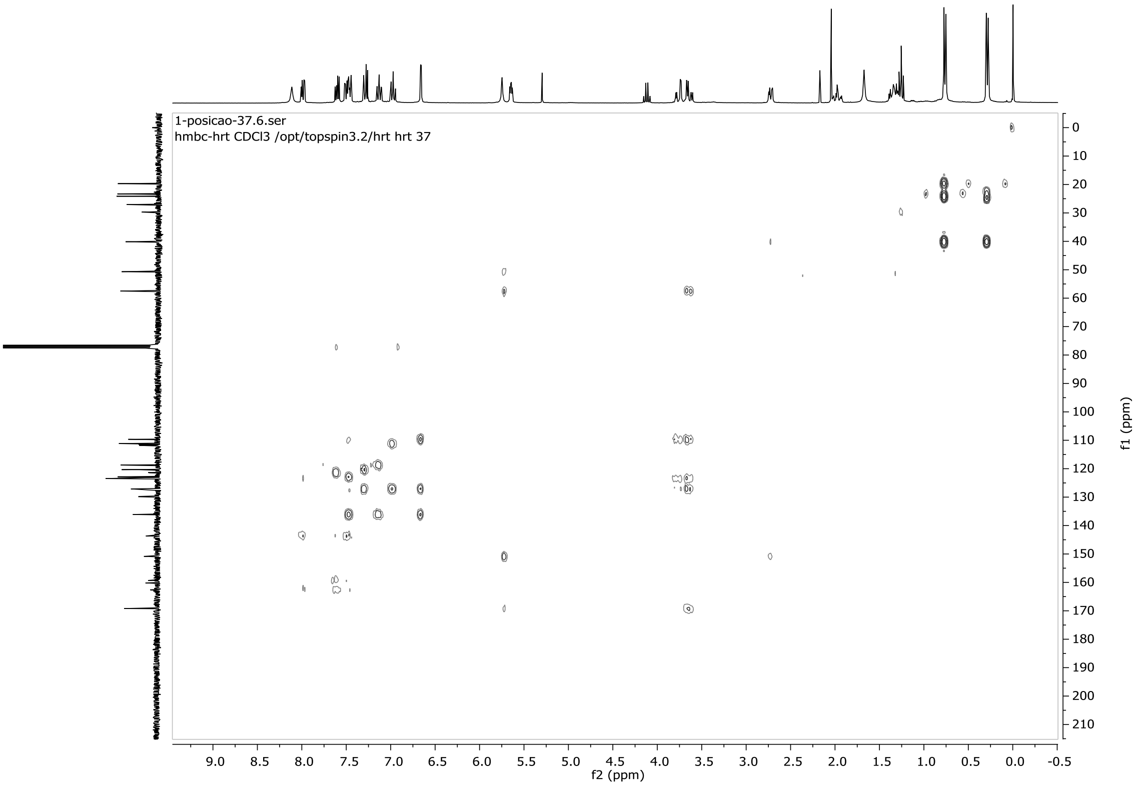

Figure S35 HMBC spectrum for (1*S*,4*R*)-4-((1*H*-indol-3-yl)methyl)-8-fluoro-1-isobutyl-1,2-dihydro-6*H*-pyrazino[2,1-*b*]quinazoline-3,6(4*H*)-dione (**12a**).

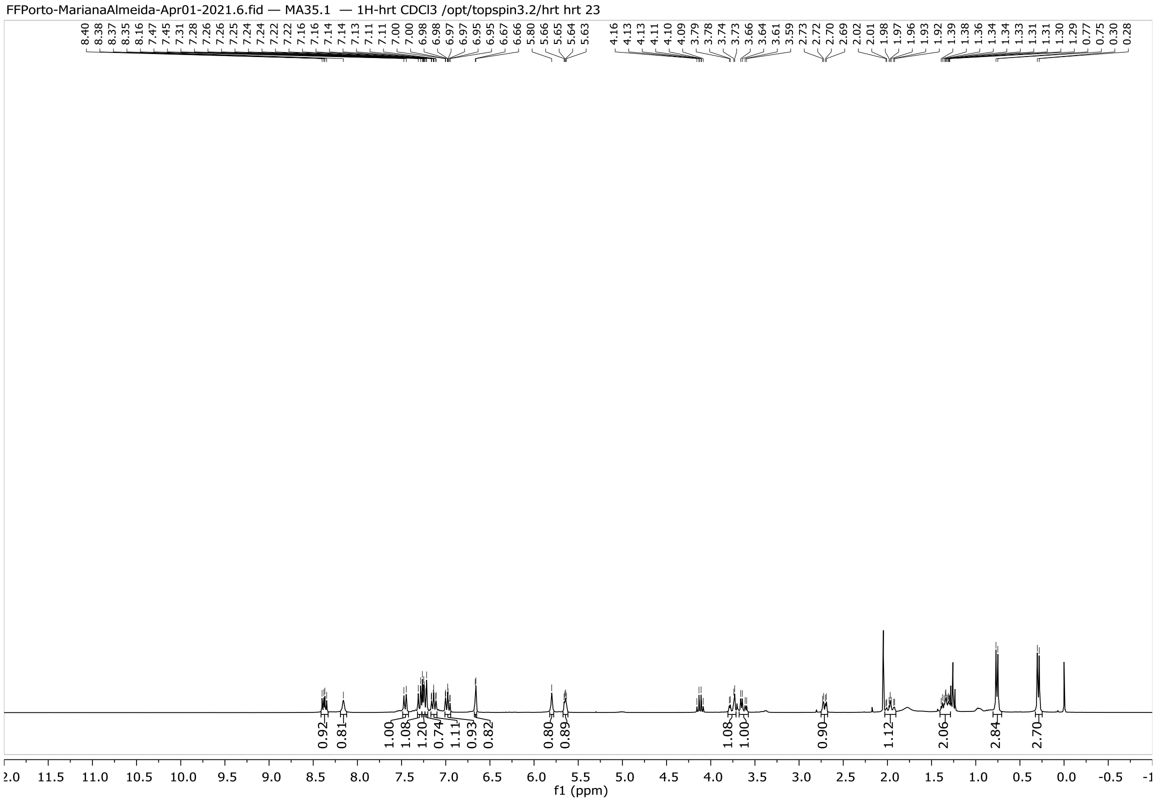

Figure S36 ^{1}H NMR (300 MHz, $CDCl_3$) spectrum for (1*S*,4*R*)-4-((1*H*-indol-3-yl)methyl)-9-fluoro-1-isobutyl-1,2-dihydro-6*H*-pyrazino[2,1-*b*]quinazoline-3,6(4*H*)-dione (**12b**).

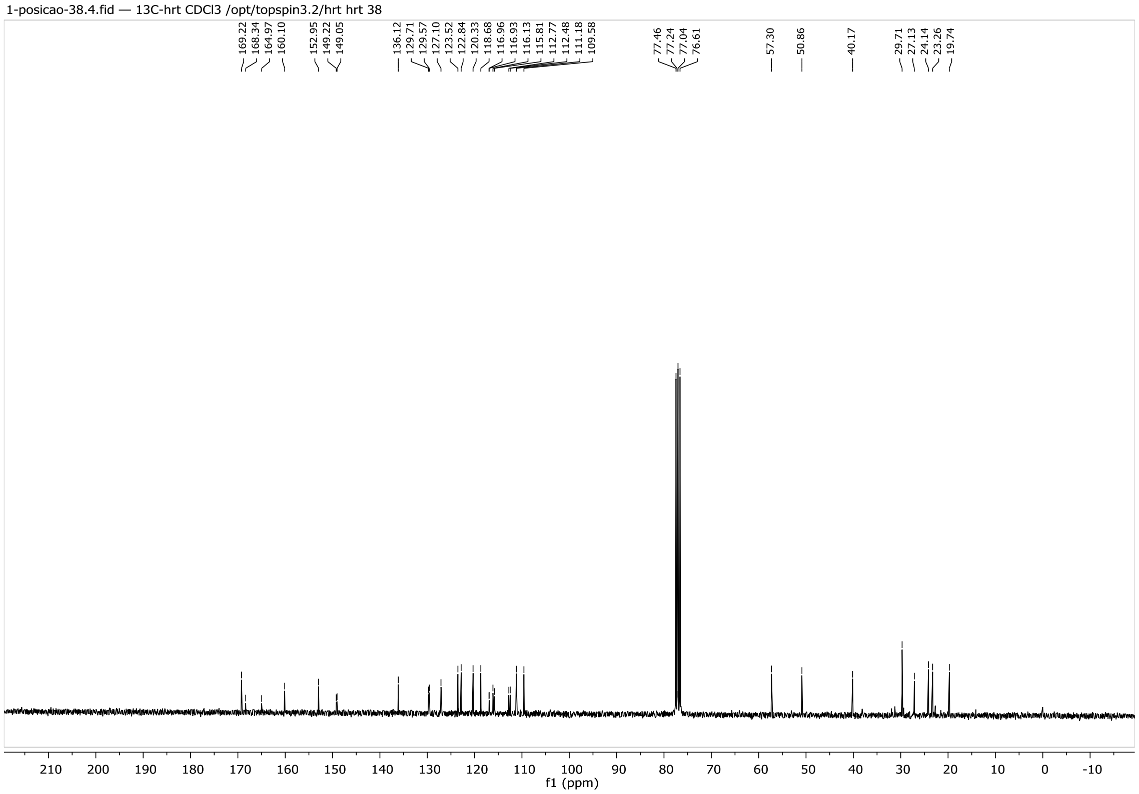

Figure S37 ^{13}C NMR (75 MHz, $CDCl_3$) spectrum for (1*S*,4*R*)-4-((1*H*-indol-3-yl)methyl)-9-fluoro-1-isobutyl-1,2-dihydro-6*H*-pyrazino[2,1-*b*]quinazoline-3,6(4*H*)-dione (**12b**).

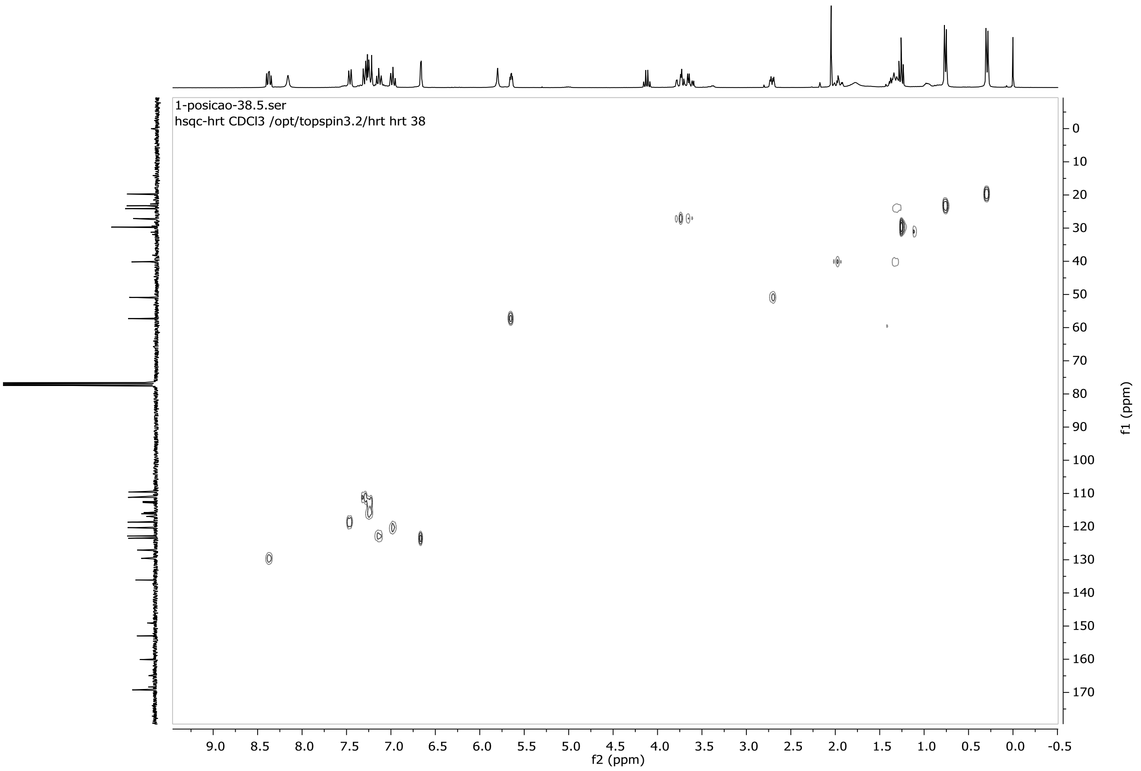

Figure S38 HSQC spectrum for (1*S*,4*R*)-4-((1*H*-indol-3-yl)methyl)-9-fluoro-1-isobutyl-1,2-dihydro-6*H*-pyrazino[2,1-*b*]quinazoline-3,6(4*H*)-dione (**12b**).

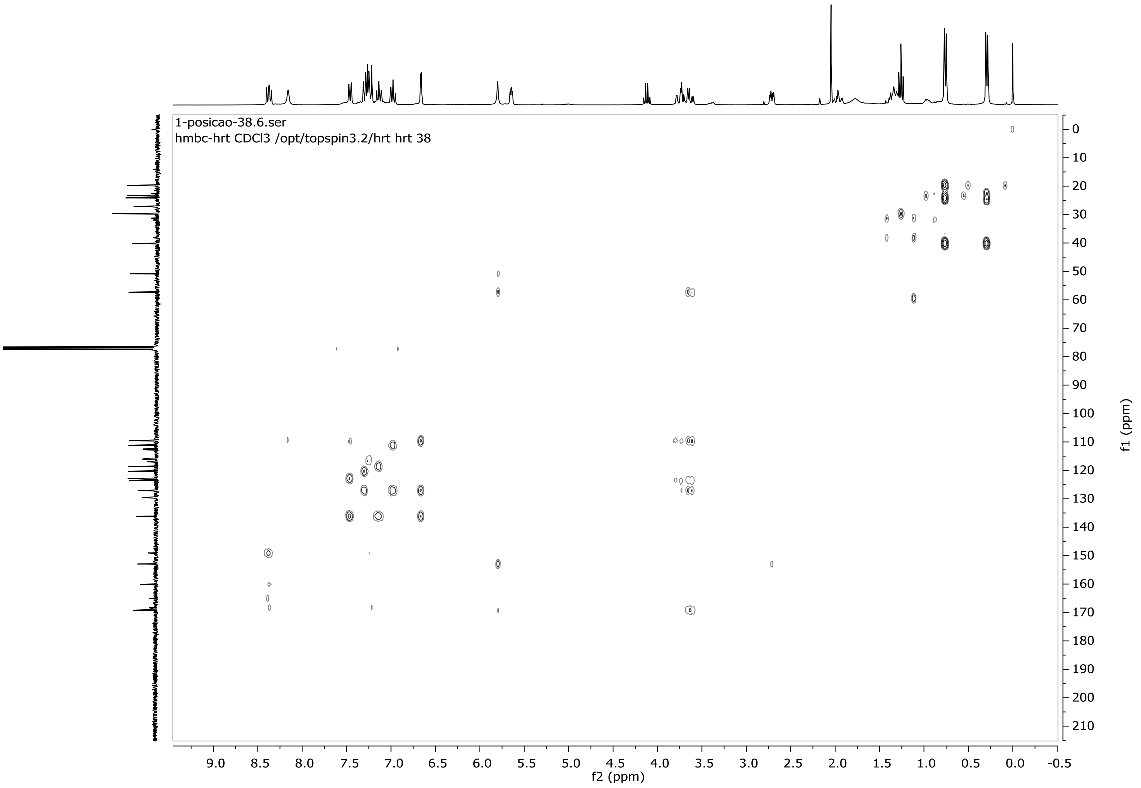

Figure S39 HMBC spectrum for (1*S*,4*R*)-4-((1*H*-indol-3-yl)methyl)-9-fluoro-1-isobutyl-1,2-dihydro-6*H*-pyrazino[2,1-*b*]quinazoline-3,6(4*H*)-dione (**12b**).

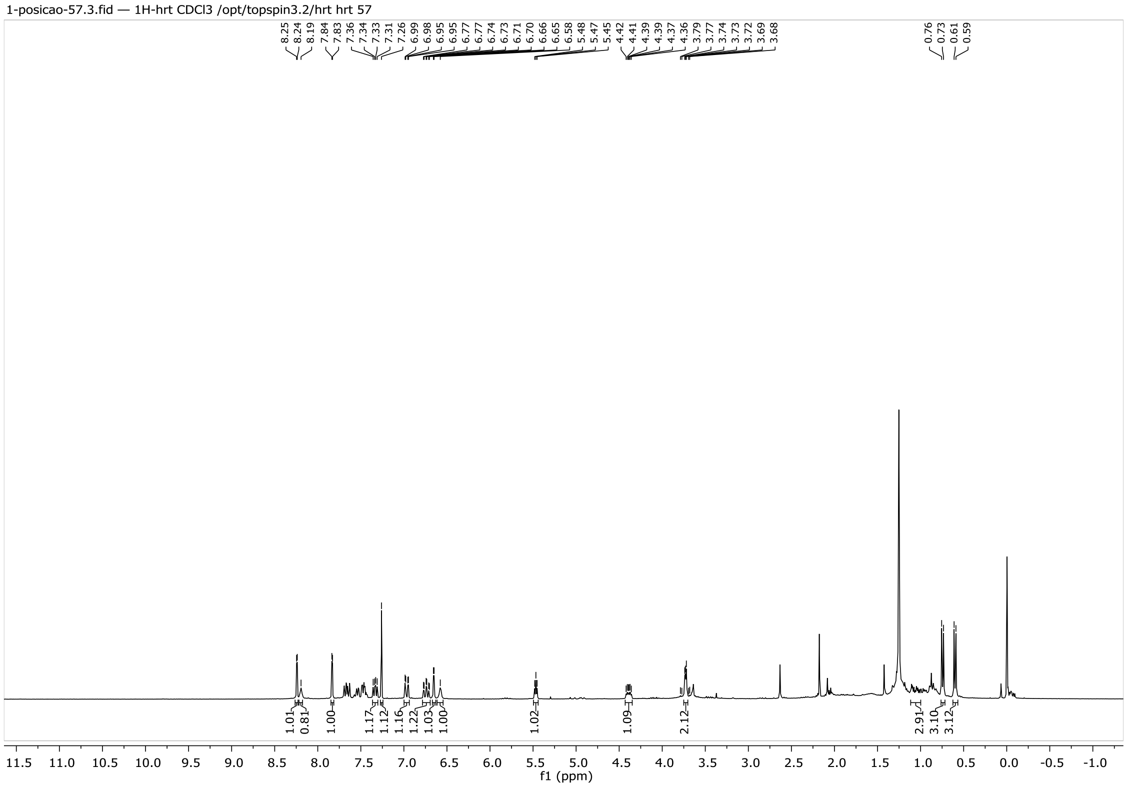

Figure S40 ^{1}H-NMR (300 MHz, $CDCl_3$) spectrum for (1*S*,4*S*)-8,10-dichloro-4-((6-fluoro-1*H*-indol-3-yl)methyl)-1-isobutyl-1,2-dihydro-6*H*-pyrazino[2,1-*b*]quinazoline-3,6(4*H*)-dione (**12c**).

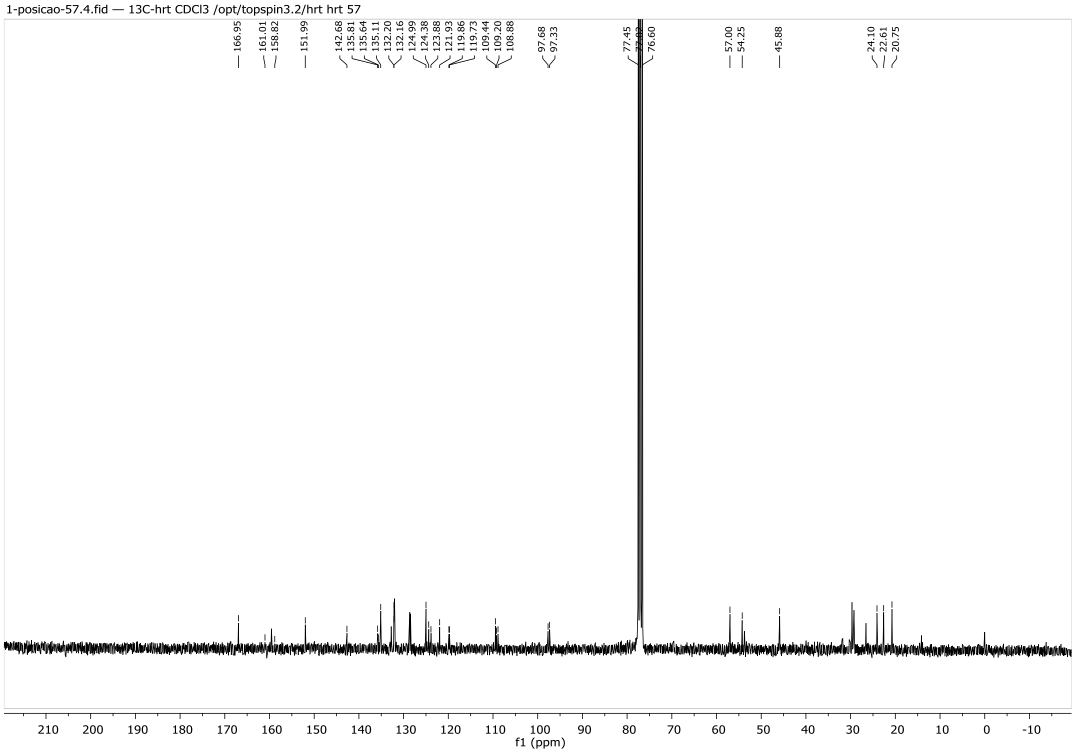

Figure S41 ^{13}C-NMR (75 MHz, $CDCl_3$) spectrum for (1*S*,4*S*)-8,10-dichloro-4-((6-fluoro-1*H*-indol-3-yl)methyl)-1-isobuty-1,2-dihydro-6*H*-pyrazino[2,1-*b*]quinazoline-3,6(4*H*)-dione (**12c**).

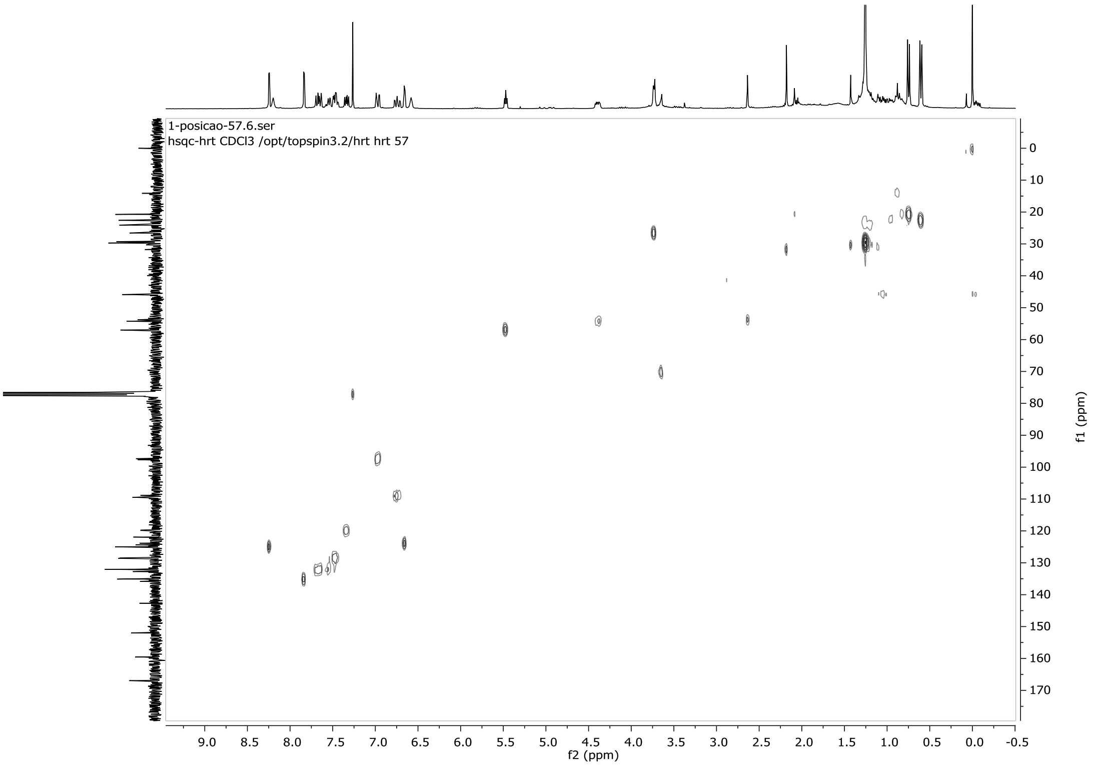

Figure S42 HSQC spectrum for (1*S*,4*S*)-8,10-dichloro-4-((6-fluoro-1*H*-indol-3-yl)methyl)-1-isobutyl-1,2-dihydro-6*H*-pyrazino[2,1-*b*]quinazoline-3,6(4*H*)-dione (**12c**).

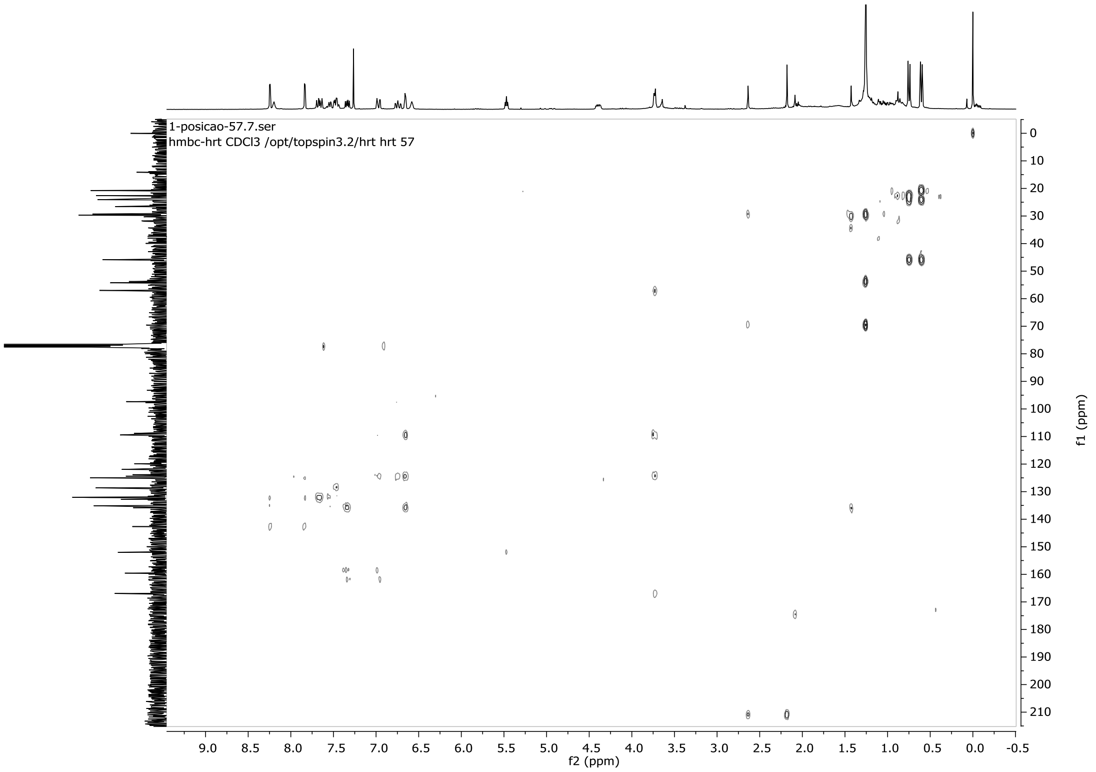

Figure S43 HMBC spectrum for (1*S*,4*S*)-8,10-dichloro-4-((6-fluoro-1*H*-indol-3-yl)methyl)-1-isobutyl-1,2-dihydro-6*H*-pyrazino[2,1-*b*]quinazoline-3,6(4*H*)-dione (**12c**).

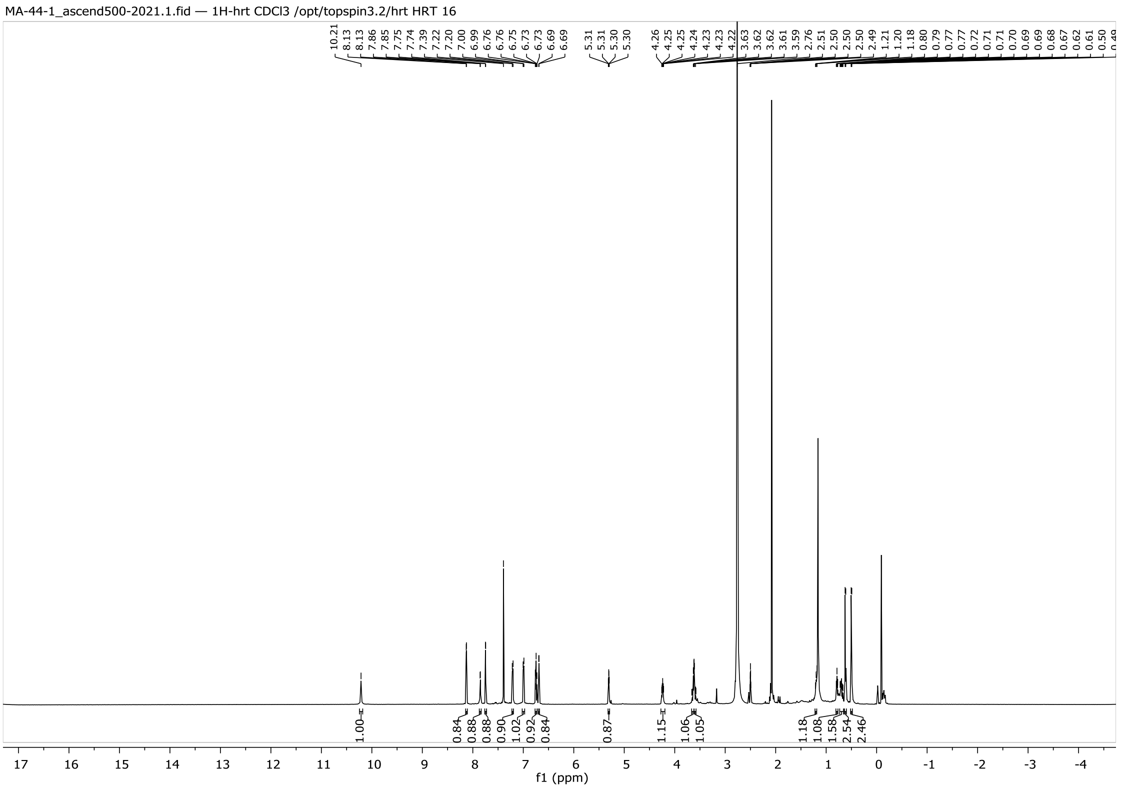

Figure S44 ^{1}H-NMR (500 MHz, $CDCl_3$) spectrum for (1*S*,4*S*)-8,10-dichloro-4-((7-chloro-1*H*-indol-3-yl)methyl)-1-isobutyl-1,2-dihydro-6*H*-pyrazino[2,1-*b*]quinazoline-3,6(4*H*)-dione (**12d**).

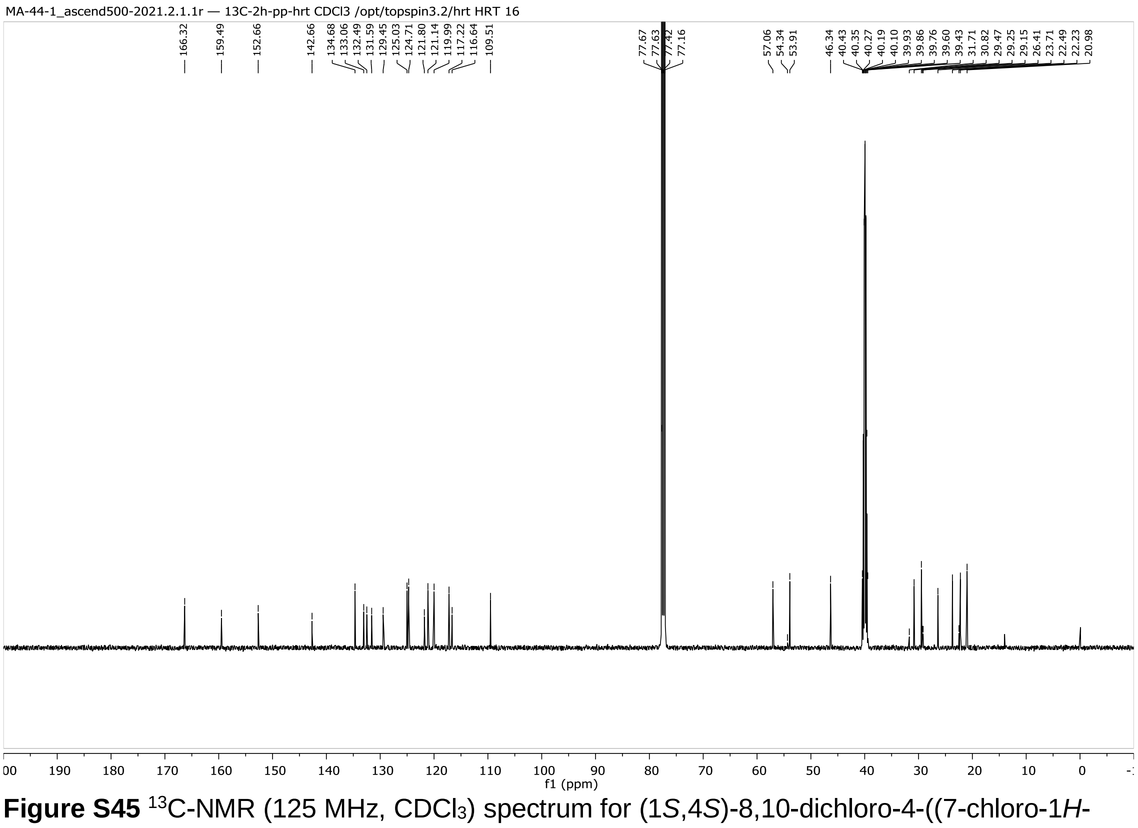

Figure S45 ^{13}C-NMR (125 MHz, $CDCl_3$) spectrum for (1*S*,4*S*)-8,10-dichloro-4-((7-chloro-1*H*-indol-3-yl)methyl)-1-isobutyl-1,2-dihydro-6*H*-pyrazino[2,1-*b*]quinazoline-3,6(4*H*)-dione (**12d**).

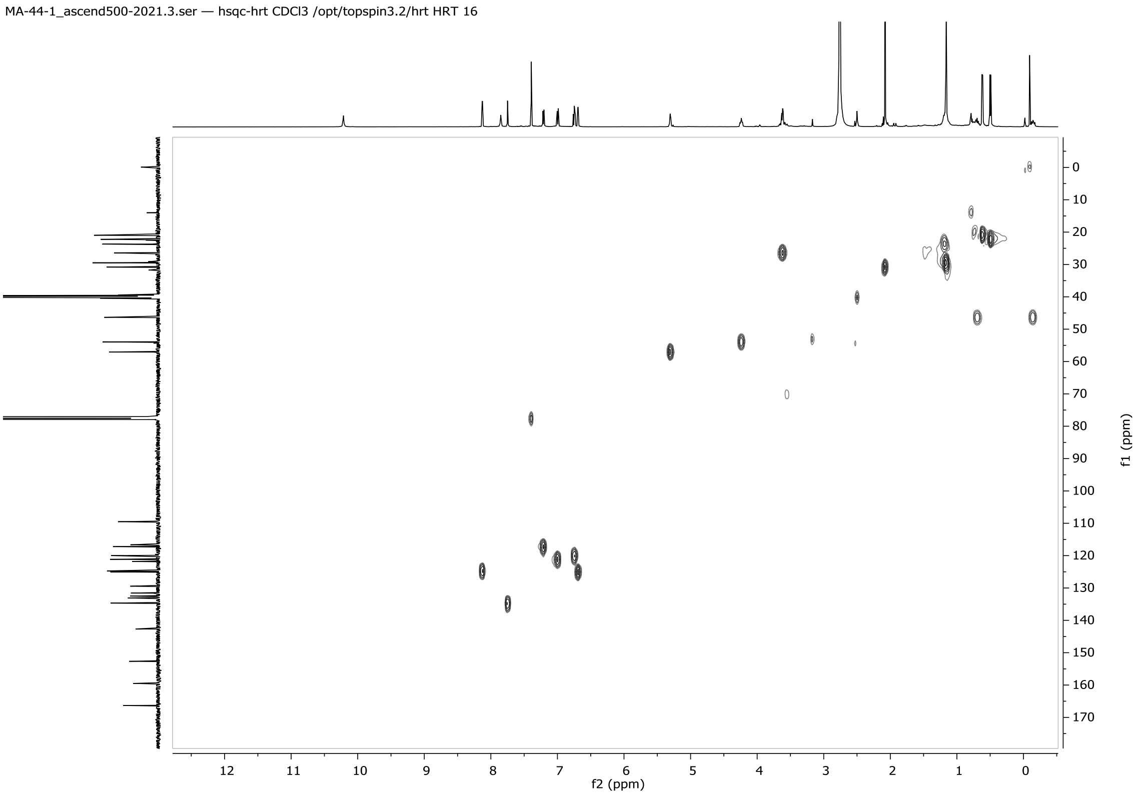

Figure S46 HSQC spectrum for (1*S*,4*S*)-8,10-dichloro-4-((7-chloro-1*H*-indol-3-yl)methyl)-1-isobutyl-1,2-dihydro-6*H*-pyrazino[2,1-*b*]quinazoline-3,6(4*H*)-dione (**12d**).

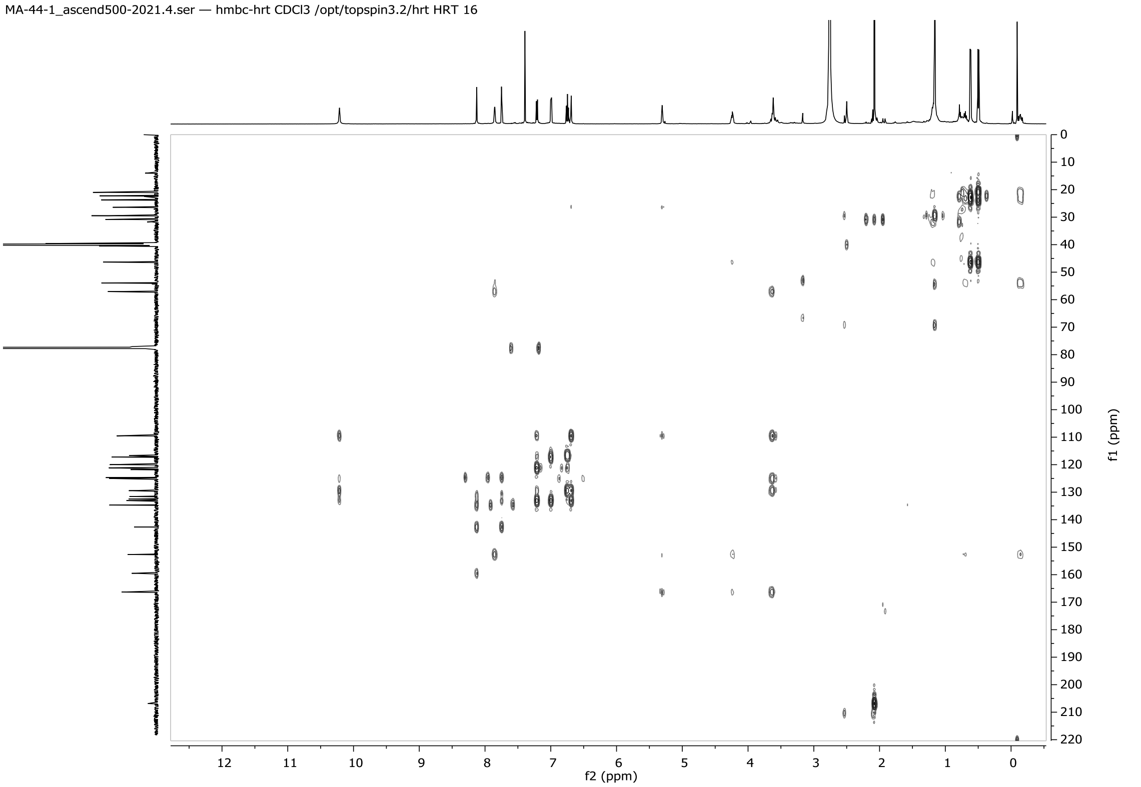

Figure S47 HMBC spectrum for (1*S*,4*S*)-8,10-dichloro-4-((7-chloro-1*H*-indol-3-yl)methyl)-1-isobutyl-1,2-dihydro-6*H*-pyrazino[2,1-*b*]quinazoline-3,6(4*H*)-dione (**12d**).

2. HRMS Data

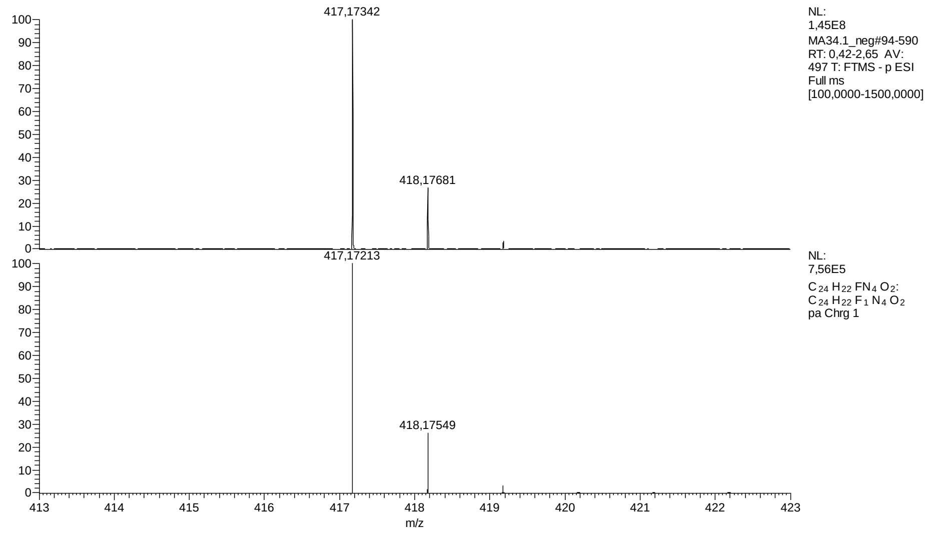

Elemental composition search on mass 417,17

m/z= 412,17-422,17

m/z	Theo. Mass	Delta (ppm)	RDB equiv.	Composition
417,17342	417,17213	3,09	15,5	$C_{24}H_{22}O_2N_4F$

Figure S48. HRMS data of (1S,4R)-4-((1H-indol-3-yl)methyl)-8-fluoro-1-isobutyl-1,2-dihydro-6H-pyrazino[2,1-b]quinazoline-3,6(4H)-dione (**12a**).

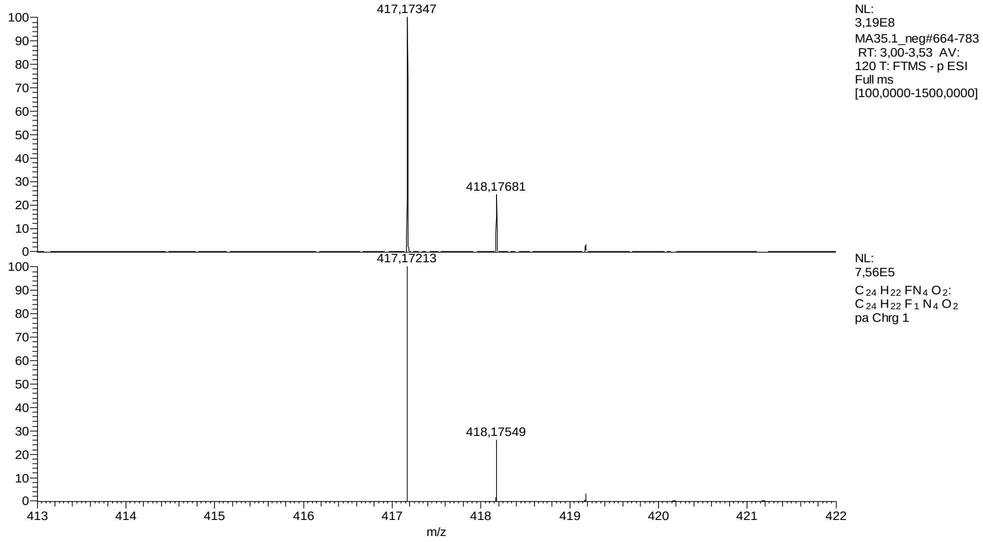

Elemental composition search on mass 417,17

m/z= 412,17-422,17

m/z	Theo. Mass	Delta (ppm)	RDB equiv.	Composition
417,17347	417,17213	3,21	15,5	$C_{24}H_{22}O_2N_4F$

Figure S49. HRMS data of (1*S*,4*R*)-4-((1*H*-indol-3-yl)methyl)-9-fluoro-1-isobutyl-1,2-dihydro-6*H*-pyrazino[2,1-b]quinazoline-3,6(4*H*)-dione (**12b**).

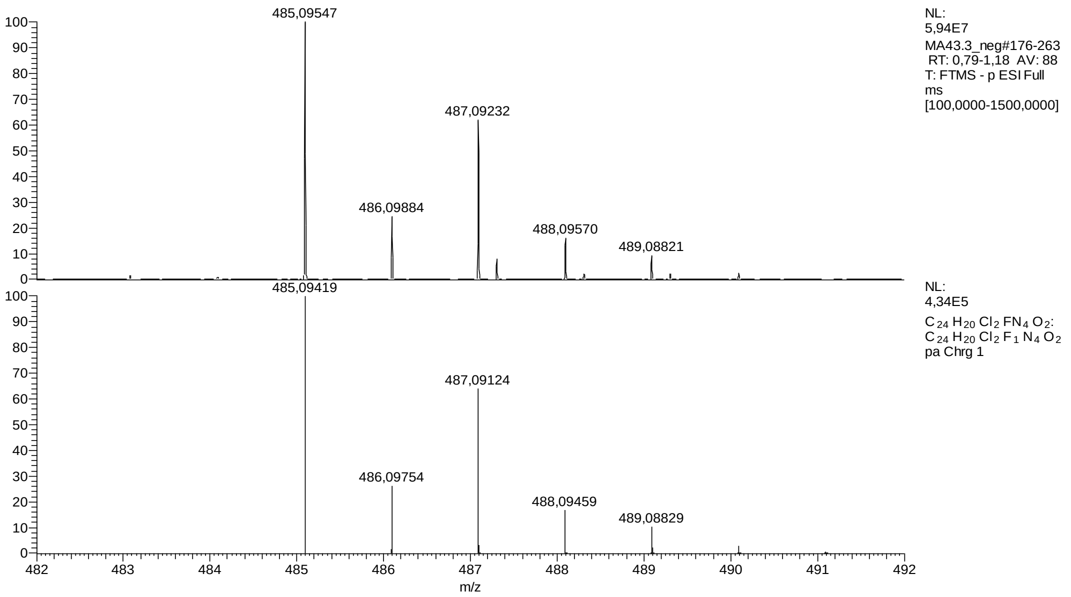

Elemental composition search on mass 485,10

m/z= 480,10-490,10

m/z	Theo. Mass	Delta (ppm)	RDB equiv.	Composition
485,09547	485,09419	2,65	15,5	$C_{24}H_{20}O_2N_4Cl_2F$

Figure S50. HRMS data of (1*S*,4*S*)-8,10-dichloro-4-((6-fluoro-1*H*-indol-3-yl)methyl)-1-isobutyl-1,2-dihydro-6*H*-pyrazino[2,1-*b*]quinazoline-3,6(4*H*)-dione (**12c**).

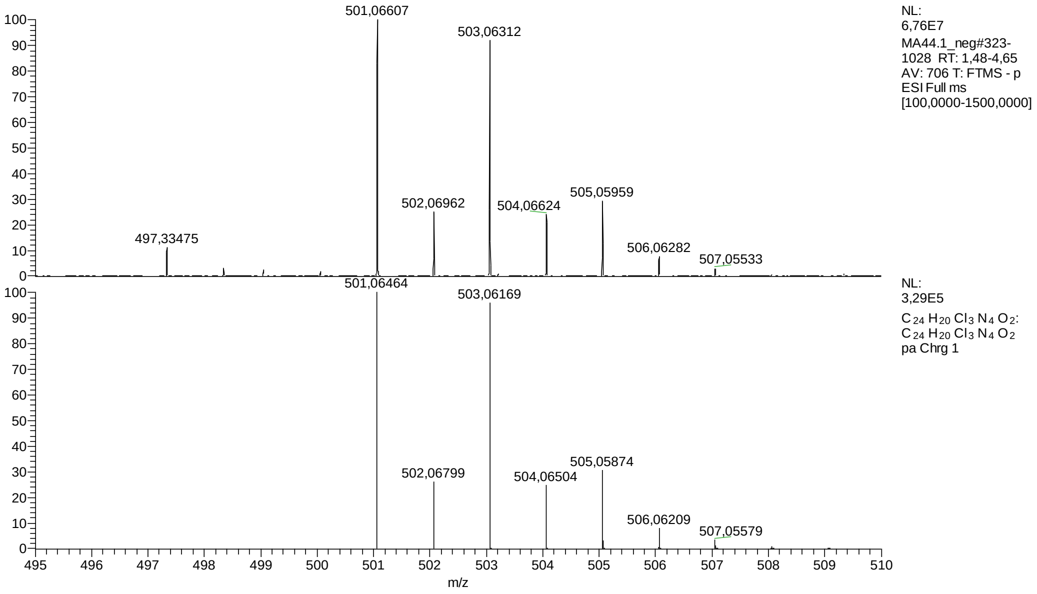

Elemental composition search on mass 501,07

m/z= 496,07-506,07

m/z	Theo. Mass	Delta (ppm)	RDB equiv.	Composition
501,06607	501,06464	2,86	15,5	$C_{24}H_{20}O_2N_4Cl_3$

Figure S51. HRMS data of (1*S*,4*S*)-8,10-dichloro-4-((7-chloro-1*H*-indol-3-yl)methyl)-1-isobutyl-1,2-dihydro-6*H*-pyrazino[2,1-*b*]quinazoline-3,6(4*H*)-dione (**12d**).

3. HPLC-DAD Purity Data

Table S1 HPLC-DAD purity data of compounds **1**, **2**, **12a-12d**.

**1**	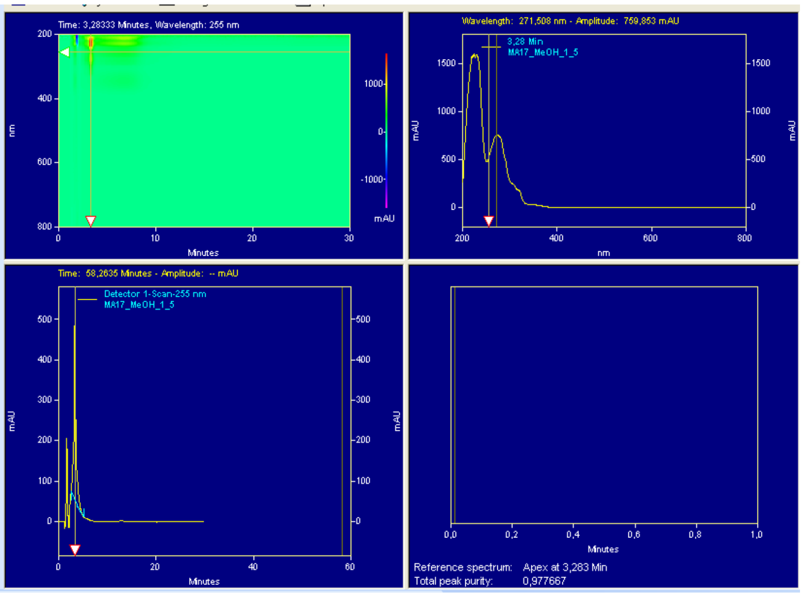
**2**	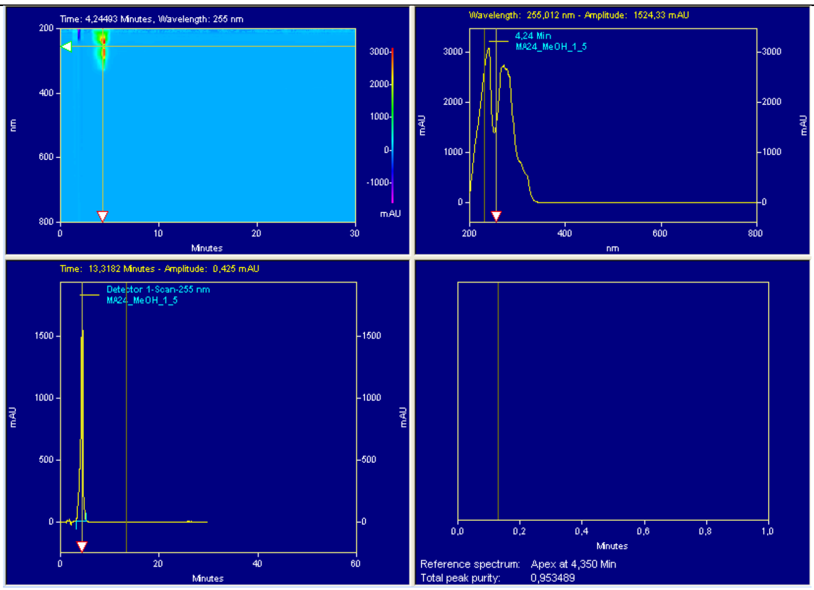

12a

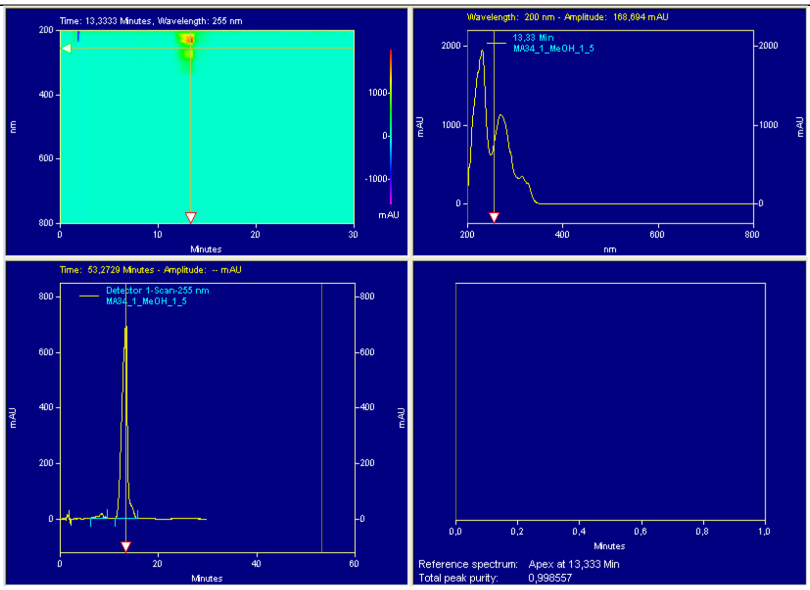

Time: 13,3333 Minutes, Wavelength: 255 nm
Wavelength: 200 nm - Amplitude: 168,694 mAU
13,33 Min
MA34_1_MeOH_1_5
Time: 53,2729 Minutes - Amplitude: -- mAU
Detector 1-Scan-255 nm
MA34_1_MeOH_1_5
Minutes
nm
mAU
Reference spectrum: Apex at 13,333 Min
Total peak purity: 0,998557

12b

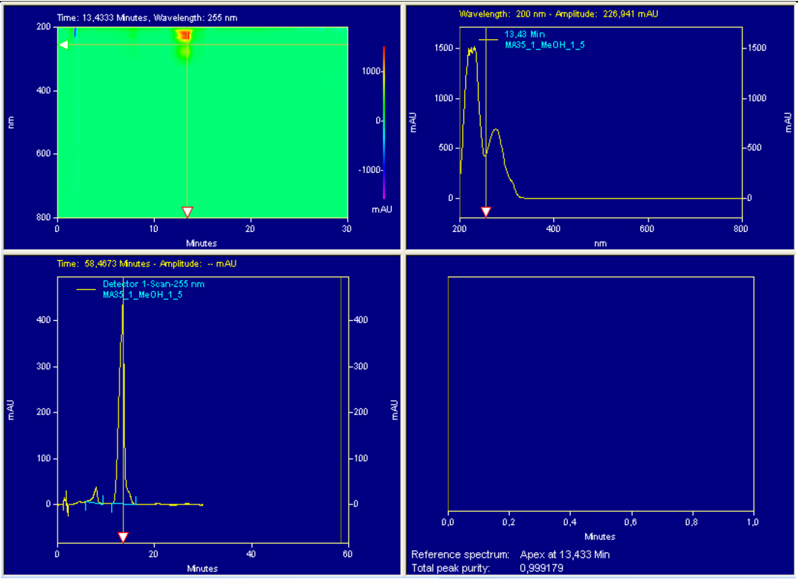

Time: 13,4333 Minutes, Wavelength: 255 nm
Wavelength: 200 nm - Amplitude: 226,941 mAU
13,43 Min
MA35_1_MeOH_1_5
Time: 58,4673 Minutes - Amplitude: -- mAU
Detector 1-Scan-255 nm
MA35_1_MeOH_1_5
Minutes
nm
mAU
Reference spectrum: Apex at 13,433 Min
Total peak purity: 0,999179

12c

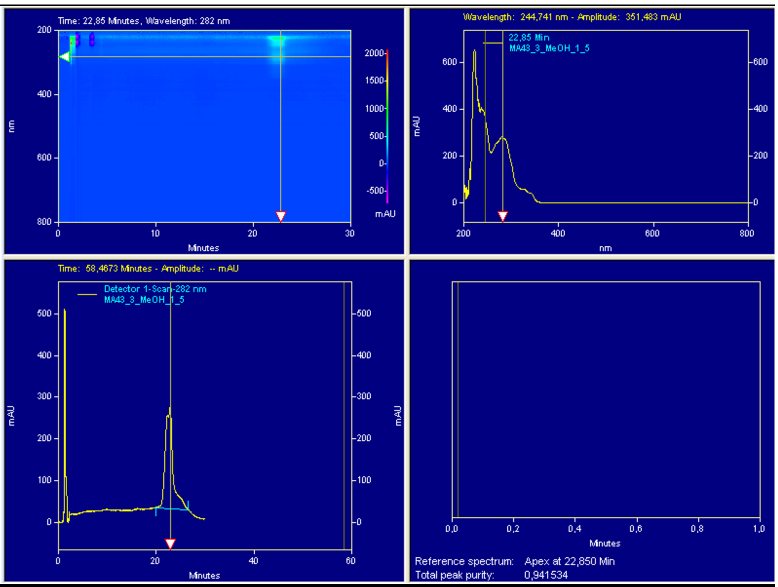

Time: 22,85 Minutes, Wavelength: 282 nm
Wavelength: 244,741 nm - Amplitude: 351,483 mAU
22,85 Min
MA43_3_MeOH_1_5
Time: 58,4673 Minutes - Amplitude: -- mAU
Detector 1-Scan-282 nm
MA43_3_MeOH_1_5
Minutes
nm
mAU
Reference spectrum: Apex at 22,850 Min
Total peak purity: 0,941534

12d

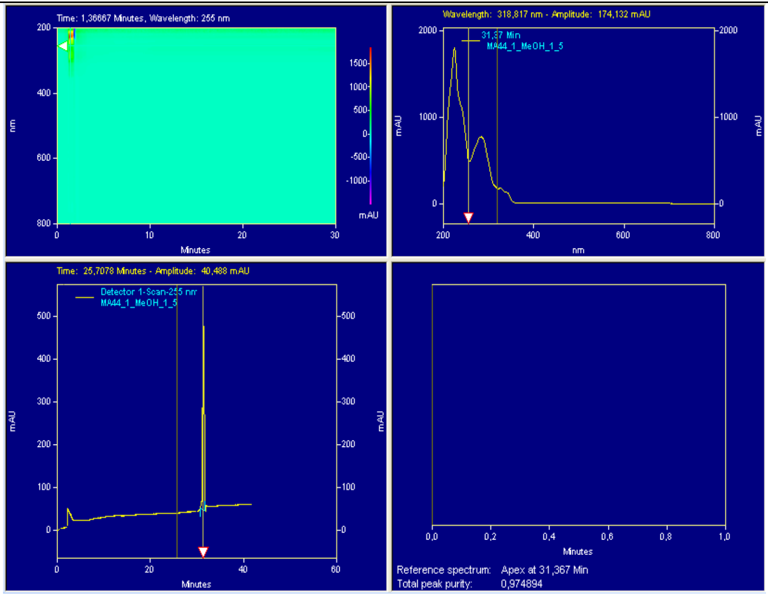
Time: 1,36667 Minutes, Wavelength: 255 nm
nm
Minutes
mAU
Wavelength: 318,817 nm - Amplitude: 174,132 mAU
31,37 Min
MA44_1_MeOH_1_5
mAU
nm
Time: 25,7078 Minutes - Amplitude: 40,488 mAU
Detector 1-Scan-255 nm
MA44_1_MeOH_1_5
mAU
Minutes
Minutes
Reference spectrum: Apex at 31,367 Min
Total peak purity: 0,974894

4. Enantiomeric Excess Data

Table S2 HPLC-DAD data from enantiomeric excess of compounds **1**, **2**, **12a**-**12d**.

Compound	Chromatogram	Retention time
**1**	254nm 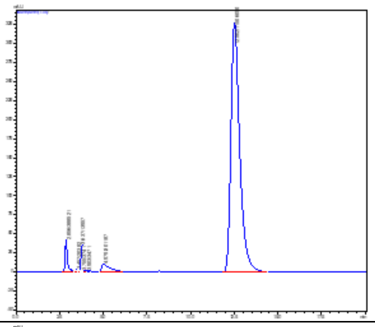	**12.50 min**
**2**	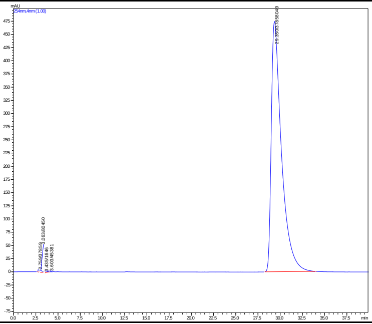	**66.16 min**
**12a**	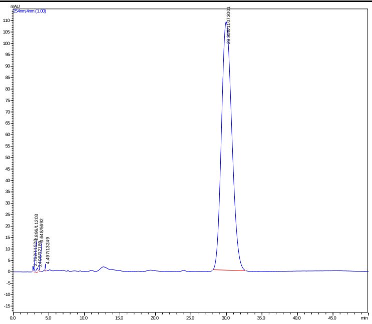	**18.48 min**
**12b**	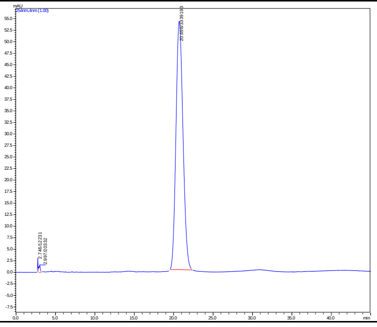	**12.27 min**

**12c**	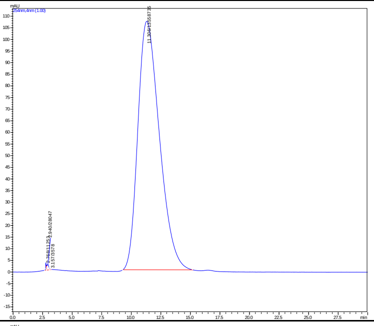	**10.66 min**
**12d**	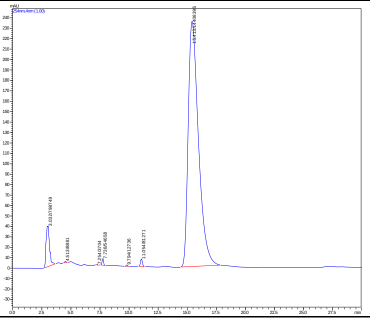	**25.41 min**

5. Docking studies

Table S3. Docking scores of the derivatives **1**, **2**, **4**, **12a**-**12d**, **13a**-**13d** and controls doxorubicin and reserpine.

**Compounds**	**Docking scores (kcal/mol)**						
	**AcrB**		**AcrA**		**TolC**	**NorA**	
	**SBS**	**HT**	**HH**	**LD**		**BCR**	**CS**
**1**	-8.9	-7.3	-6.7	-5.7	-8.4	-7.0	-6.3
**2**	-8.5	-7.4	-7.8	-4.1	-8.6	-4.5	-6.1
**4**	-9.0	-5.1	-7.4	-3.5	-8.1	-1.0	-5.4
**12a**	-8.9	-6.9	-7.3	-6.0	-8.3	-5.5	-5.7
**12b**	-8.9	-6.5	-7.4	-5.4	-8.7	-7.1	-5.5
**12c**	-9.5	-2.7	-5.8	-2.7	-7.6	-3.8	-5.1
**12d**	-8.7	-6.5	-7.2	-3.3	-7.8	-0.6	-5.6
**13a**	-8.8	-6.1	-6.3	-5.4	-7.5	-2.9	-5.4
**13b**	-8.4	-7.1	-7.4	-3.3	-7.6	-3.5	-4.9
**13c**	-8.9	-7.0	-6.7	-6.1	-7.9	-2.2	-5.5
**13d**	-10.8	-5.6	-8.5	-2.7	-8.9	-2.1	-3.9
**Doxorubicin**	-8.9	15.4	-7.2	-5.6	-7.2	-	-
**Reserpine**	-	-	-	-	-	1.0	-4.6

SBS: Substrate-binding site; **HT**: Hydrophobic trap; **HH**: Helical hairpin; **LD**: Lipoyl domain; **BCR**: Binding core region; **CS**: Cytoplasmic side; **TMD**: Transmembrane domain; **NBD**: Nucleotide binding domain.

Concerning the higher predicted affinity of the compounds to the CS of the NorA homology models, this was the site chosen for molecular visualization of the compounds effective in the inhibition of ethidium bromide efflux in the *S. aureus* 272123 model. Upon a first inspection, it can be noted that compounds **12a** (pink) and **13c** (orange) have a predicted binding site different than all the other compounds (Figure S52) and did not show any hydrogen interactions with neighboring residues. However, weaker interaction may still happen, such as van der Waals.

A closer look into the predicted interactions of compounds **4** (Figure S52B), **12b** (Figure S52C), **12c** (Figure S52D), and **13b** (Figure S52E) show a hydrogen bond with Asn-128, a polar residue. On a different note, compound **13a** (Figure S52F) is predicted to interact with the non-polar residue Gly-305. However, it must be taken into account that there is no evidence that the compounds are inhibiting NorA, and that these docking studies were performed in a homology model, which may not reflect the real residues involved.

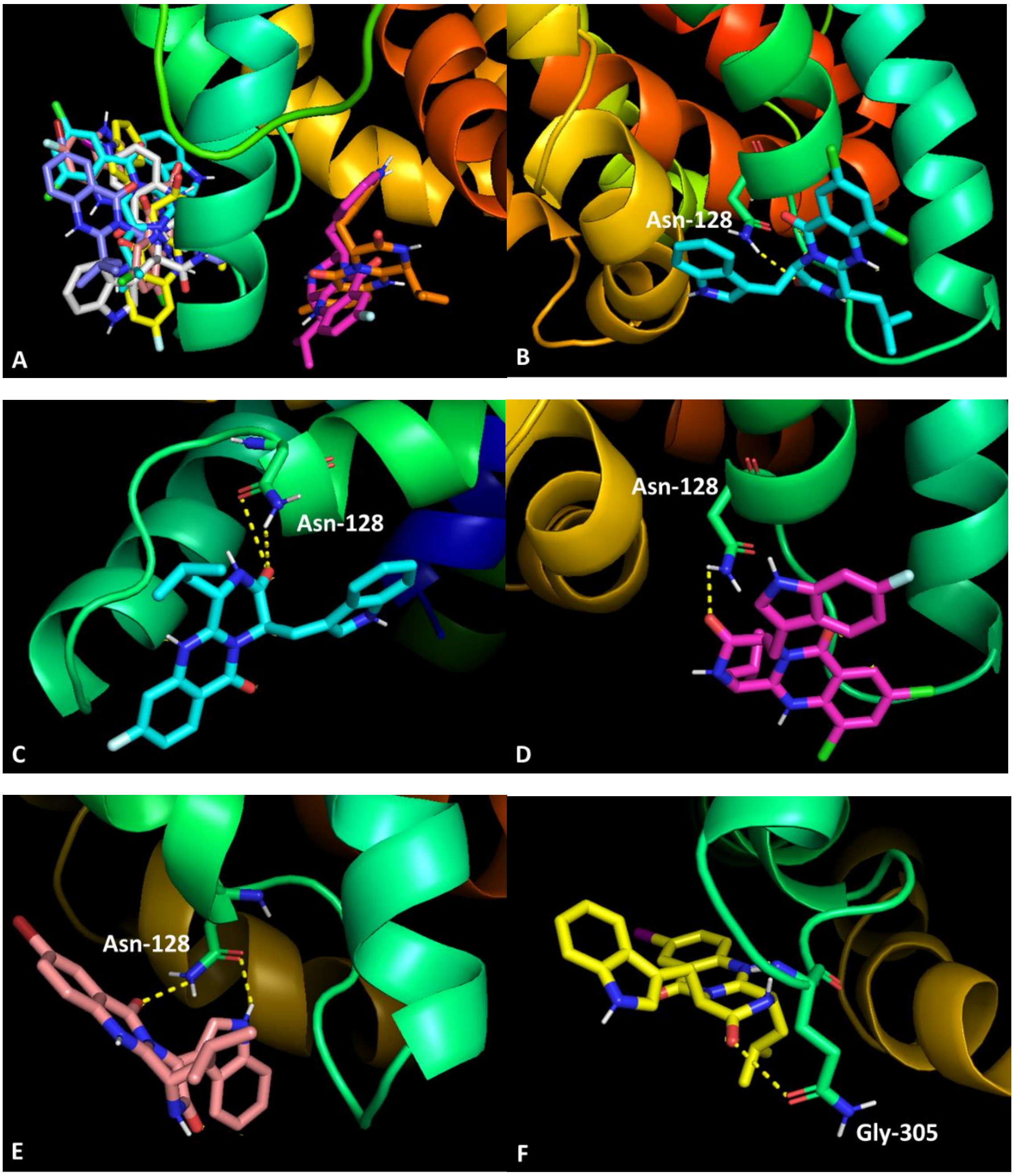

Figure S52. Molecular visualization of derivatives in the CS of NorA. (**A**) General view of the compounds in the CS; (**B**) Interaction between compound **4** and the CS; (**C**) Interaction between compound **12b** and the CS; (**D**) Interaction between compound **12c** and the CS; (**E**) Interaction between compound **13b** and the CS; (**F**) Interaction between compound **13a** and the CS.
